# EP300 inhibition alleviates acute-on-chronic liver failure through modulation of scar-associated macrophages

**DOI:** 10.1038/s41467-026-78245-x

**Published:** 2026-10-07

**Authors:** Panyu Chen, Bingyuan Huang, Wentao Chen, Xiao Lin, Changjie Yang, Chen Wang, Wenxi Su, Zhaokai Zeng, Yunyun Ren, Shijie Fan, Yun Song, Rong Fu, Yixuan Xu, Xiaosong Chen, Xiaogang Xiang, Guangming Li, Kaixian Chen, Xiong Ma, Yugang Wang, Yuanyuan Zhang, Min Shi, Cheng Luo

**Affiliations:** 1 Department of Gastroenterology, Tongren Hospital, Shanghai Jiao Tong University School of Medicine, Shanghai, China; 2 State Key Laboratory of Drug Research, Shanghai Institute of Materia Medica, Chinese Academy of Sciences, Shanghai, China; 3 Key Laboratory for Translational Research and Innovative Therapeutics of Gastrointestinal Oncology, Tongren Hospital, Shanghai Jiao Tong University School of Medicine, Shanghai, China; 4 Division of Gastroenterology and Hepatology, Renji Hospital, Shanghai Jiao Tong University School of Medicine, Shanghai, China; 5 University of Chinese Academy of Sciences, Beijing, China; 6 Department of Liver Surgery, Renji Hospital, Shanghai Jiao Tong University School of Medicine, Shanghai, China; 7 Department of Infectious Disease, Renji Hospital, Shanghai Jiao Tong University School of Medicine, Shanghai, China; 8 Hongqiao International Institute of Medicine, Tongren Hospital, Shanghai Jiao Tong University School of Medicine, Shanghai, China; 9 Department of Gastroenterology, Xinhua Hospital, Shanghai Jiao Tong University School of Medicine, Shanghai, China; 10 Zhongshan Institute for Drug Discovery, Shanghai Institute of Materia Medica, Chinese Academy of Sciences, Zhongshan, Guangdong China; 11 Department of Infectious Diseases, Ruijin Hospital, Shanghai Jiao Tong University School of Medicine, Shanghai, China; 12 Clinical Cancer Institute, Center for Translational Medicine, Naval Medical University, Shanghai, China; 13 Precision Medicine Research Institute of Guizhou, and Guizhou Provincial Key Laboratory of Digestive System Diseases, The Affiliated Hospital of Guizhou Medical University, Guiyang, China

**Keywords:** Phagocytes, Liver diseases, Pharmacodynamics

## Abstract

Acute-on-chronic liver failure is a severe syndrome with high short-term mortality, and current treatments are limited to liver transplantation and supportive care. Here we show that EP300 inhibition alleviates acute-on-chronic liver failure by targeting scar-associated macrophages, a subset that proliferates during disease progression and whose impaired antibacterial function is linked to poor prognosis. EP300 inhibition enhances the antibacterial activity of scar-associated macrophages through transcriptional repression of ACOD1. ACOD1 expression is induced in scar-associated macrophages during acute-on-chronic liver failure, and its loss mitigates hepatic destruction and prolongs survival in male mice with acute-on-chronic liver failure by enhancing the phagocytic function of scar-associated macrophages. These findings suggest that EP300 inhibition alleviates acute-on-chronic liver failure through ACOD1-dependent reprogramming of scar-associated macrophages, providing a promising therapeutic strategy for acute-on-chronic liver failure.

## Abstract (web summary)

Acute-on-chronic liver failure is a life-threatening condition with limited treatment options. Here, the authors show that EP300 inhibition improves disease outcomes by enhancing the antibacterial function of scar-associated macrophages through ACOD1-dependent reprogramming.

## Introduction

Acute-on-chronic liver failure (ACLF) is a severe clinical syndrome characterized by acute deterioration of chronic liver disease. It is marked by jaundice, coagulopathy, and a high risk of extrahepatic organ failure, leading to short-term mortality^1–3^. Patients with chronic liver disease frequently exhibit compromised immune function^4–7^, rendering them vulnerable to various triggers such as hepatitis B virus (HBV) reactivation, alcohol consumption, and bacterial and fungal infections. These factors can further deteriorate immune function and exacerbate ACLF^4,8,9^. Up to two-thirds of ACLF patients develop infections at diagnosis or during follow-up, regardless of etiology, with bacterial infection being a major prognostic determinan^4^. Although prophylactic antibiotics reduce the incidence of bacterial infections, they do not significantly improve patient outcomes, probably due to the high prevalence of multidrug-resistant bacteria^10^. Therefore, strategies aimed at restoring immune function to combat bacterial infections may provide promising therapeutic options for ACLF patients.

Previous studies have shown that monocytes in ACLF patients exhibit widespread innate immune defects, including impaired phagocytic and inflammatory functions^11,12^. TREM2^+^CD9^+^ macrophages, known as scar-associated macrophages (SAMs), were initially identified in the fibrotic niche of cirrhotic livers^13,14^. In liver cirrhosis, SAMs originate from circulating monocytes and display a pro-fibrogenic phenotype^13,14^. However, the regulation and function of SAMs in ACLF remains poorly understood. Aconitate decarboxylase 1 (ACOD1), also known as immune-responsive gene 1 (IRG1), is an immunometabolic mediator that is upregulated in mononuclear phagocytes (MPs) under stress, particularly in response to pathogen-associated molecular pattern (PAMP) molecules such as lipopolysaccharide (LPS)^15,16^. Depletion of ACOD1 in myeloid cells has been shown to protect mice against polymicrobial sepsis by reducing systemic inflammation^9^. Whether ACOD1 is induced in SAMs and contributes to ACLF progression remains unknown.

In this study, we demonstrate that SAMs accumulate along the ACLF progression and whose impaired antibacterial function is linked to poor prognosis. Inhibition of EP300 alleviates ACLF progression by reprogramming SAMs mediated by ACOD1. *ACOD1* depletion attenuates hepatic destruction and prolongs ACLF mice survival through improved phagocytic capacity of SAMs. EP300 inhibitor CCS1477 transcriptionally represses *ACOD1* expression by reducing EP300/CBP recruitment. Clinically, immunoparalysis denotes depressed antimicrobial effector functions despite ongoing inflammation. Our data position SAM immunoparalysis in ACLF as a reprogrammable state governed by the EP300-ACOD1 axis, rather than an inherent property of SAMs. Moreover, the EP300 inhibitor CCS1477 represents a potential candidate for clinical translation in ACLF treatment.

## Results

### Increased abundance of monocyte-derived SAMs correlates with poor prognosis in ACLF patients

**Fig. 1:**
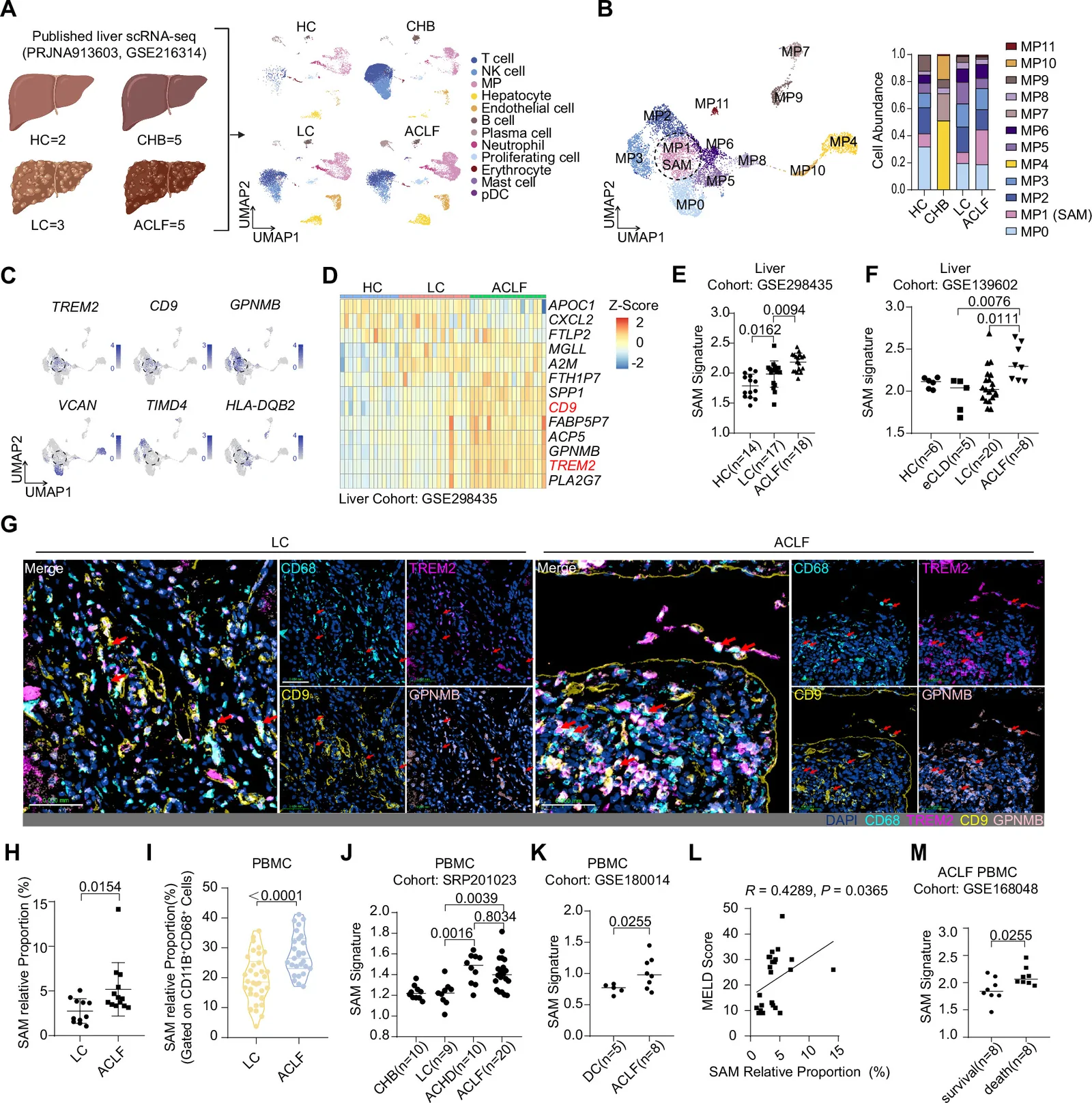
Increased abundance of monocyte-derived SAMs correlates with poor prognosis in ACLF patients. **A** Pattern diagram (left panel) and UMAP embedding of all cell types (right panel) from integrated liver datasets. Created in BioRender. Yu, P. (2026) https://BioRender.com/p43c600. **B** UMAP embedding of MP subpopulations from human livers (left panel) and their proportions at different stages of HC and HBV-related liver disease (right panel). **C** Expression of human MP marker genes shown on UMAP embedding. **D** Heatmap of the corresponding signature expression profiles in GSVA scores of SAMs in human liver bulk RNA-seq data (**E**) GSVA scores of SAMs at HC and different stages of HBV-related liver disease from human liver bulk RNA-seq data. **F** GSVA scores of SAMs at HC and different stages of non-HBV-related liver disease from human liver bulk RNA-seq data. **G** Representative mIHC images showing the positivity of CD68, TREM2, CD9, and GPNMB in the livers of LC and ACLF patients (*n* = 11 for LC and *n* = 13 for ACLF, biologically independent human samples). Scale bars, 0.05 mm. Red arrows represent the location of SAMs. **H** Quantification of (**G**), SAMs (CD68^+^TREM2^+^CD9^+^GPNMB^+^) proportion of all cells in the field of view (five fields per patient). **I** Flow cytometric analysis of SAMs (TREM2^+^CD9^+^) within the CD11B^+^CD68^+^ gated population in PBMCs of LC (*n* = 34) and ACLF patients (*n* = 27). **J** GSVA scores of SAMs at different stages of HBV-related liver disease from human PBMC bulk RNA-seq data. **K** GSVA scores of SAMs at different stages of non-HBV-related liver disease from human PBMC bulk RNA-seq data. **L** Correlation of SAMs’ proportion in mIHC with MELD score. **M** GSVA scores of SAMs from PBMC bulk RNA-seq data in ACLF patients with survival and death outcomes. In (**E**, **F**, **I**, **J**–**M**), sample sizes (*n*) represent biologically independent human samples. Bars represent mean ± SD. In (**E**, **F**, **J**), statistical significance was calculated using one-way ANOVA followed by Tukey’s multiple-comparisons test. In (**H**), statistical significance was calculated using a two-sided Mann-Whitney *U* test. In (**I**, **M**), statistical significance was calculated using a two-sided unpaired Student’s *t* test. In (**K**), statistical significance was calculated using a two-sided unpaired Welch’s *t* test. In (**L**), correlation was assessed using Spearman rank correlation. HC, healthy controls; CHB, chronic hepatitis B; LC, liver cirrhosis; ACLF, acute-on-chronic liver failure; MP, mononuclear phagocytes; SAM, scar-associated macrophages; eCLD, early chronic liver disease; ACHD, acute-on-chronic hepatic dysfunction; DC, decompensated cirrhosis. Source data are provided as a Source Data File.

To systematically investigate the hepatic immune microenvironment during ACLF progression, we analyzed single-cell RNA-sequencing (scRNA-seq) data from 15 publicly available human liver samples, including healthy controls (HC), chronic hepatitis B (CHB), liver cirrhosis (LC), and ACLF (Fig. 1A). A total of 30,687 non-parenchymal cells (NPCs) were categorized into 12 cell types based on the signatures of known lineage markers (Fig. 1A, S1A-S1C). The population of MPs was higher in ACLF than in CHB or LC (Fig. S1A). We identified 12 MP subpopulations and found that MP1 was significantly increased in ACLF (Fig. 1B). These MP1 subsets were identified as SAMs based on the known markers (TREM2^+^CD9^+^)^13,14^ (Fig. 1C, S1D). Gene Set Variation Analysis (GSVA) score, calculated through bulk RNA-Seq, further demonstrated an increased SAM signature in ACLF liver compared to LC. (Fig. 1D–F).

SAMs in cirrhotic livers are derived from circulating monocytes^13,14^. Pseudotime analysis of scRNA-seq revealed that these SAMs represented an intermediate differentiation stage, primarily originating from the MP0 subcluster (VCAN^+^FCN1^+^S100A9^+^ monocytes), and subsequently differentiated into mature macrophages, such as those in the MP3 cluster (SLC8A1^+^MSR1^+^) (Figs. S1D, S2A). In addition, we identified genes and transcription factors (TFs) that may regulate SAM differentiation (Figs. S2B, S2C).

To assess the correlation between SAMs and ACLF progression, we first evaluated SAMs in liver from both LC and ACLF patients. Compared with LC livers, ACLF livers exhibited increased infiltration of SAMs (Fig. 1G, H). Then we try to figure out SAMs’ location. In HBV-related cirrhotic patients, SAMs were primarily enriched in the portal areas surrounding collagen-positive scar regions (Figs. S3, S4). This enrichment was also observed in non-HBV cirrhosis^14^. When disease progressed to ACLF, livers showed poorly defined portal and lobular structures and broader SAM infiltration around the portal areas (Fig. S3). This spatial pattern was also confirmed in both HBV-related and alcohol-related ACLF (Figs. S3–S5). Although necrotic regions extending along the central veins are a characteristic feature of HBV-ACLF^17^, SAMs were rarely distributed around the central veins (Fig. S6). Two stages’ data indicate that SAMs’ localization is independent of the etiology of ACLF or necrotic zones but follows the pattern observed in LC, which may reflect a spatial predisposition for antibacterial immune defense during ACLF^18,19^. SAM populations were also elevated in ACLF peripheral blood mononuclear cells (PBMCs) compared to those in LC or DC (Decompensated Cirrhosis) (Fig. 1I–K) and were significantly correlated with Model for End-Stage Liver Disease (MELD) scores (Fig. 1L). Additionally, the increased SAM signature in PBMCs was associated with mortality outcomes in ACLF patients (Fig. 1M). The aforementioned results suggest that SAM accumulation is associated with poor prognosis in ACLF patients.

### EP300 is a regulator of SAMs in ACLF

**Fig. 2:**
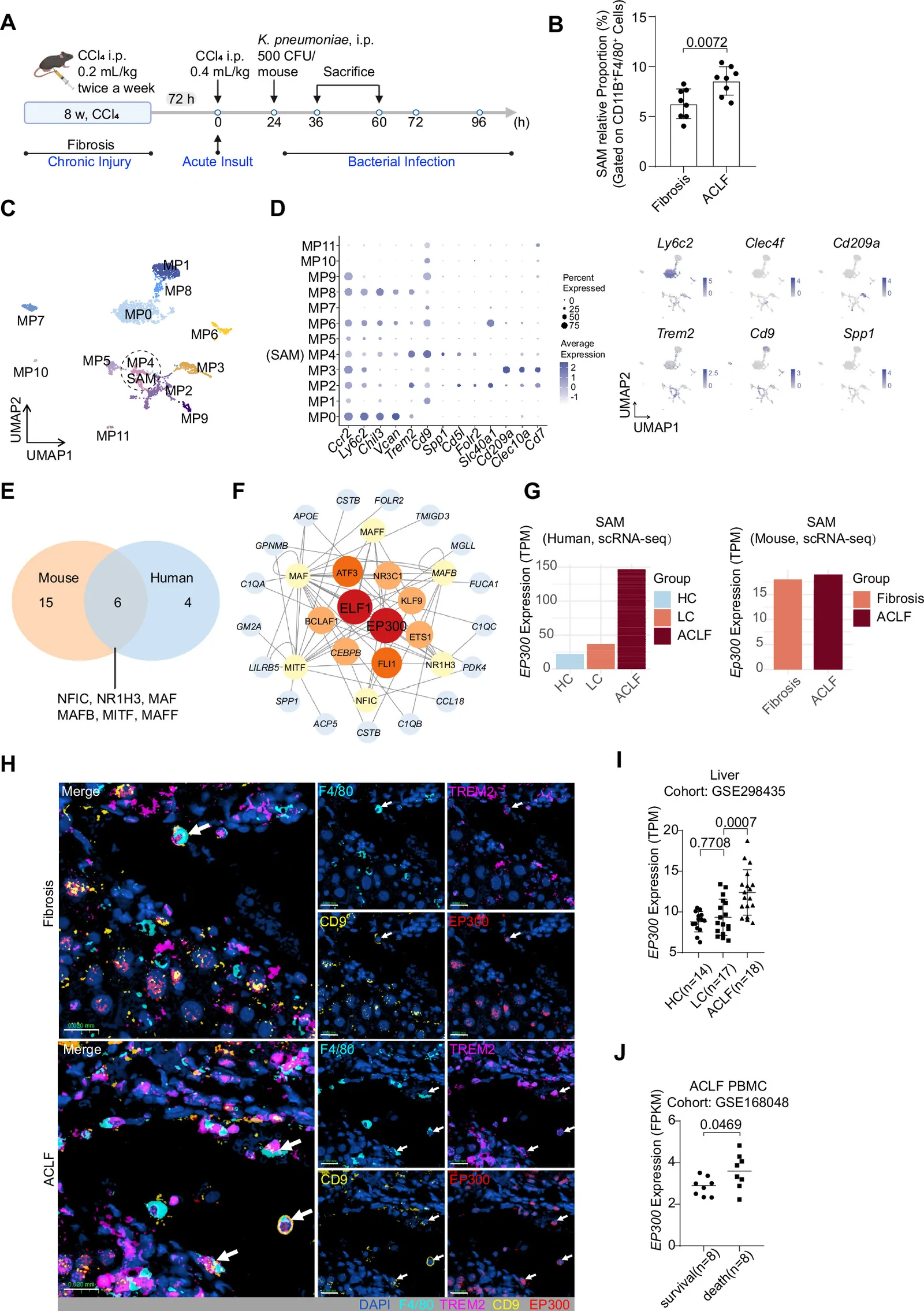
EP300 is a regulator of SAMs in ACLF. **A** Schematic timeline of the modeling procedures of liver fibrosis and ACLF. Created in BioRender. Yu, P. (2026) https://BioRender.com/a21e252. **B** Flow cytometry analysis of SAMs (TREM2^+^CD9^+^) within the CD11B^+^F4/80^+^ gated population in the livers of fibrosis and ACLF model mice (*n* = 8 biologically independent mice samples per group). **C** UMAP embedding of MP subpopulations from the livers of fibrosis and ACLF model mice. **D** Representative marker genes for identification of MPs subsets. **E** Venn diagram showing the intersection of up-regulated TFs in both human and mice data during SAM differentiation. **F** Prediction of key regulators for SAMs (outer blue circles represent genes regulated by the six light yellow circles and that continuously increasing during SAM differentiation; six light yellow circles denote the intersection TFs in (E); inner circles display the prediction results, where darker colors indicate more promising results). **G** EP300 expression in SAMs revealed by single-cell transcriptomic analyses of human and mouse livers. Y axis represents relative expression level (TPM). **H** Representative mIHC images of EP300 showed regions of colocalization with SAMs (F4/80^+^TREM2^+^CD9^+^, white arrows) in mice livers (*n* = 5 biologically independent mice samples per group). Scale bars, 0.05 mm. **I** Expression level of *EP300* at HC and different stages of HBV-related liver disease from human liver bulk RNA-seq data. Sample sizes (*n*) represent biologically independent human samples. The y-axis represents relative expression level (TPM). **J** Expression level of *EP300* from PBMC bulk RNA-seq data in ACLF patients with survival and death outcomes. Sample sizes (*n*) represent biologically independent human samples. The y-axis represents relative expression level (FPKM). Bars represent mean ± SD. In (**B**, **J**), statistical significance was calculated using a two-sided unpaired Student’s *t* test. In (**I**), statistical significance was calculated using one-way ANOVA followed by Tukey’s multiple-comparisons test. CCl_4_, carbon tetrachloride; K. *pneumoniae, Klebsiella pneumoniae*; CFU, colony-forming units; TPM, transcripts per million; FPKM, fragments per kilobase of transcript per million mapped reads. Source data are provided as a Source Data File.

Bacterial infection plays a critical role in exacerbating ACLF, with up to two-thirds of patients developing infections at diagnosis or during follow-up^4^. To model this clinically relevant process, we employed the established ACLF mouse models^20^ and induced infection by injecting *Klebsiella pneumoniae* (K. *pneumoniae*) (Fig. 2A). The mouse model successfully reproduced the extensive disruption and progressive collapse of fibrous septa in pseudo-lobules seen in ACLF patients (Fig. S3, S7A). The serum alanine aminotransferase (ALT) and total bilirubin (TBIL) peaked at 36 h after the acute carbon tetrachloride (CCl_4_) challenge and subsequently declined (Fig. S7B). Moreover, the abundance of SAMs (TREM2^+^CD9^+^) increased in the livers of ACLF mice compared to those with fibrosis (Fig. 2B). Their enrichment in the mouse hepatic portal area (Fig. S8) was also consistent with observations in patients (Figs. S3–S5). To identify potential therapeutic targets that modulate SAMs in ACLF, we isolated CD45^+^ immune cells from mouse livers and performed scRNA-seq to characterize SAMs and their upstream regulators. Using lineage markers and phenotypic genes, we clustered 32,314 immune cells into seven major types and further refined the MP population into 12 subclusters (Fig. S9A, S9B), and refined the MP population into 12 subclusters (Figs. 2C, S9C). The MP4 subpopulation exhibited high expression of *Trem2* and *Cd9*, resembling SAMs observed in ACLF patients (Fig. 2D).

We next sought to identify a regulator of SAMs for therapeutic targeting. We evaluated the developmental trajectory of SAMs and identified 6 TFs (NFIC, NR1H3, MAF, MAFB, MITF and MAFF) that were conservatively expressed in both human and mouse ACLF livers (Figs. 2E, S2C, S9D). Through SCENIC analysis, EP300 was identified as the most prominent upstream regulator mediating the expression of the majority of these TFs (Fig. 2F). Indeed, EP300 is a well-recognized chromatin remodeler that regulates the gene transcription in a context-dependent manner^21^. scRNA-seq data from human and mouse livers showed that *EP300* is expressed in SAMs and is upregulated in ACLF compared with LC or fibrosis (Fig. 2G). mIHC also demonstrated regions of colocalization between EP300 and SAMs in ACLF mouse livers (Fig. 2H). Consistently, *EP300* expression was observed to be higher in human ACLF livers compared to LC livers (Fig. 2I), and its elevated expression showed a correlation with mortality outcomes in ACLF patients (Fig. 2J). Overall, these data suggest that EP300 is a key regulator of SAMs that may participate in ACLF progression.

### Pharmacological inhibition of EP300 reduces liver damage in ACLF

**Fig. 3:**
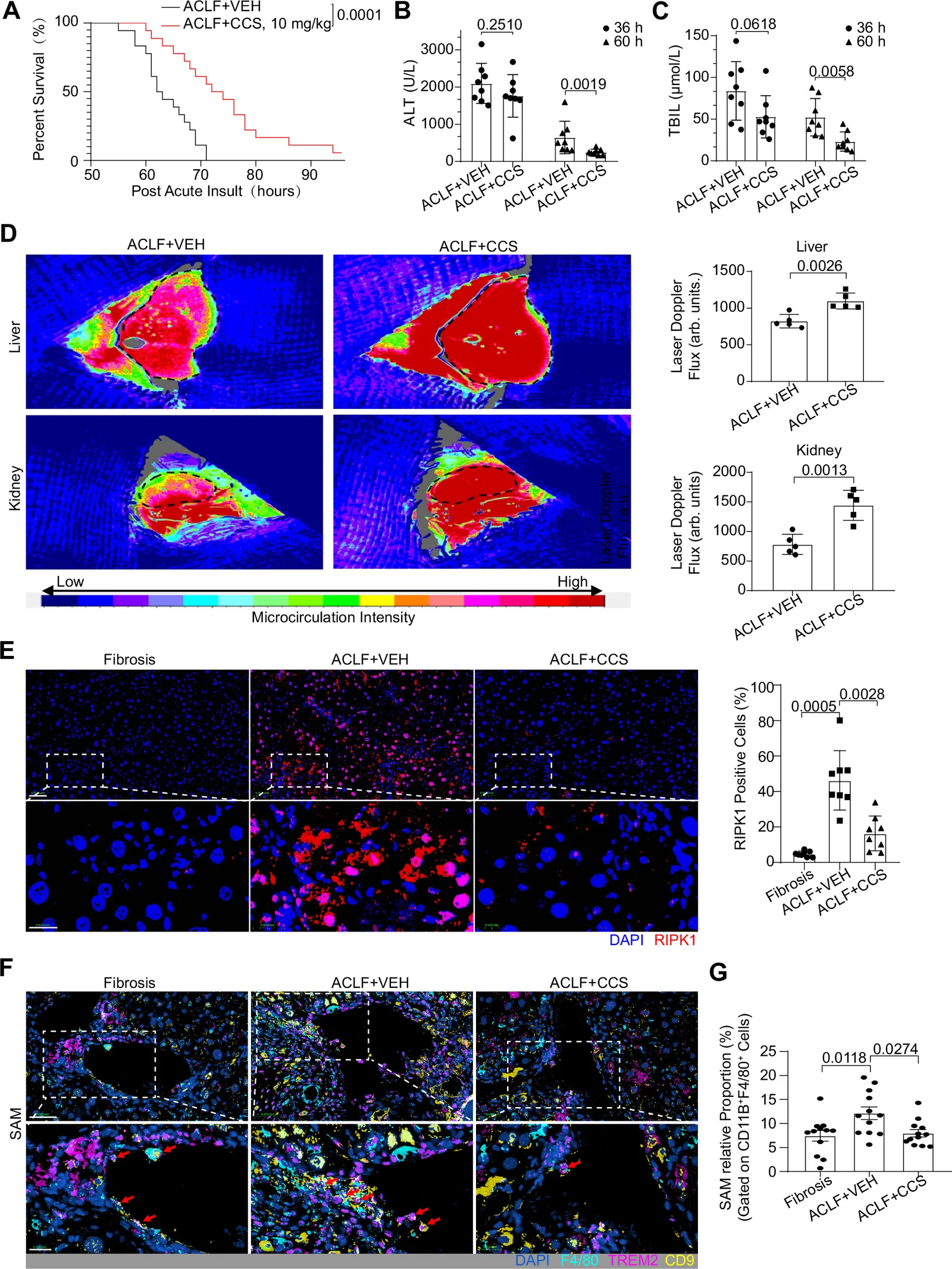
Pharmacological inhibition of EP300 reduces liver damage in ACLF. **A** Survival analysis of ACLF model mice treated with vehicle (black line) or CCS1477 (10 mg/kg, red line) (*n* = 18 biologically independent mice per group). Serum ALT (**B**) and TBIL (**C**) levels of ACLF model mice treated with vehicle or CCS1477 at 36 and 60 h post-CCl_4_ injection (*n* = 8 biologically independent mice samples per group). **D** Hepatic and left renal microcirculation were determined at 60 h post-CCl_4_ injection by Laser Doppler Flowmeter. The microcirculation intensity increases with the depth of the red color (left panel) and quantification graph (right panel) (*n* = 5 biologically independent mice per group). arb. units, arbitrary units. **E** Immunofluorescence staining of DAPI (blue) and RIPK1 (red) in mice livers with representative images (left panel) and quantification graph (right panel) (*n* = 8 biologically independent mice per group). Scale bars, 0.05 mm (upper panel) and 0.02 mm (lower panel). **F** Representative mIHC images of SAMs (F4/80^+^TREM2^+^CD9^+^, red arrows) in mice livers (*n* = 5 biologically independent mice samples per group). Scale bars, 0.05 mm (upper panel) and 0.02 mm (lower panel). **G** Flow cytometry analysis of the changes in the proportion of SAMs (TREM2^+^CD9^+^) within the CD11B^+^F4/80^+^ gated population in the liver of fibrosis and ACLF model mice with vehicle or CCS1477 (*n* = 12 biologically independent mice samples per group). Bars represent mean ± SD. In (**A**), survival curves were compared using the two-sided log-rank (Mantel–Cox) test. In (**B**), a two-sided unpaired Student’s *t* test (36 h) and a two-sided Mann-Whitney *U* test (60 h) were used. In (**C**, **D**), a two-sided unpaired Student’s *t* test was used. In (**E**), Brown-Forsythe and Welch ANOVA followed by Games-Howell’s multiple-comparisons test was used. In (**G**), one-way ANOVA followed by Tukey’s multiple-comparisons test was used. VEH, vehicle; CCS, CCS1477; arb. units, arbitrary units. Source data are provided as a Source Data File.

To examine whether EP300 inhibition can mitigate ACLF by modulating SAMs, we treated ACLF mice with CCS1477 (Fig. S10A), the most advanced p300/CBP inhibitor currently under evaluation in phase 1/2a clinical trials in hematologic malignancies^22,23^. Intraperitoneal CCS1477 significantly extended survival (Fig. 3A), comparable to that of the antibiotic imipenem (Fig. S10A, S10B). Two other EP300/CBP inhibitors, NEO2734^24^ and GNE207^25^, also prolonged survival in ACLF mice, further supporting that EP300 inhibition can alleviate ACLF (Fig. S10A, S10C). Given that CCS1477 exhibited the most robust therapeutic effect, it was chosen as the primary EP300 inhibitor in our following work for the sake of clarity and consistency.

We investigated whether CCS1477 could reduce liver damage by affecting SAMs. CCS1477 significantly lowered serum ALT and TBIL levels 60 h post-injury, indicating liver protection (Fig. 3B, C). It also enhanced liver and kidney microcirculation in ACLF mice, preventing multi-organ failure (Fig. 3D). Despite severe liver damage in ACLF, marked by necrosis and hepatocellular disintegration, CCS1477 significantly reduced this damage (Fig. S10D). RIPK1 staining showed decreased necroptosis in CCS1477-treated ACLF mice (Fig. 3E). CCS1477 did not affect liver fibrosis, as evidenced by sirius red staining (Fig. S10E). Of note, CCS1477 treatment was associated with a lower measured proportion of SAMs (Fig. 3F, G). Meanwhile, CCS1477 treatment did not alter the proportions of other macrophage subsets, including MP2 (CCR5^+^CXCR3^+^), MP6 (CXCR2^+^CD9^+^), and MP7 (CCR3^+^) (Fig. S10F). Overall, pharmacological EP300 inhibition protected the liver and improved survival, with an observed lower measured proportion of SAMs.

### Acod1-mediated SAM phagocytosis is required for the therapeutic effect of CCS1477 in ACLF

**Fig. 4:**
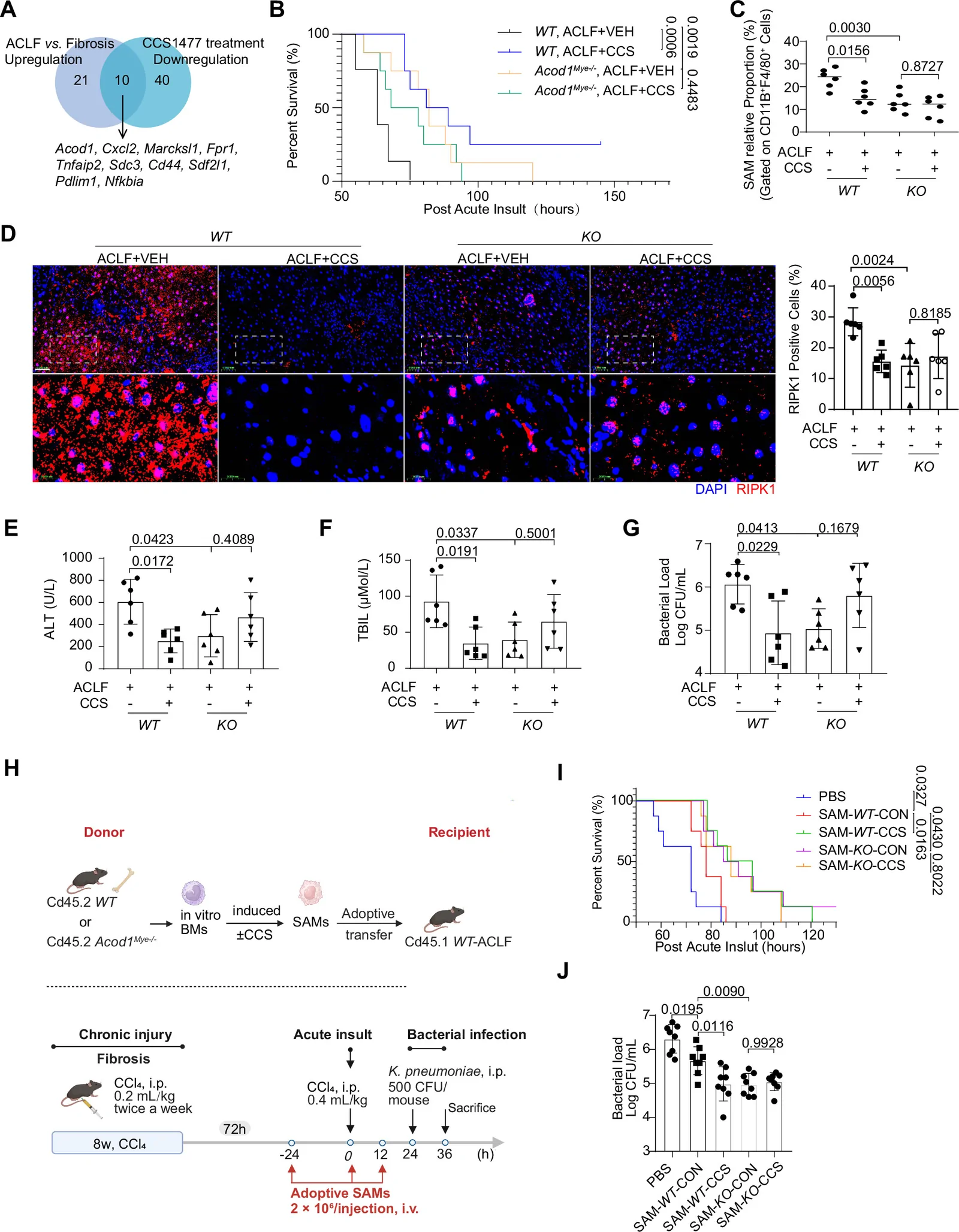
Acod1-mediated SAM phagocytosis is required for the therapeutic effect of CCS1477 in ACLF. **A** Venn diagram showing the intersection of genes up-regulated by ACLF modeling and downregulated by CCS1477 treatment in SAMs. **B** Survival analysis of WT and *Acod1*^*Mye−/−*^ ACLF model mice treated with vehicle (black line for WT and orange line for *Acod1*^*Mye−/−*^) or CCS1477 (10 mg/kg, blue line for WT and green line for *Acod1*^*Mye−/−*^) (*n* = 8 biologically independent mice per group). **C** Flow cytometry analysis of the proportion of SAMs (TREM2^+^CD9^+^) within the CD11B^+^F4/80^+^ gated population in the liver of WT and *Acod1*^*Mye−/−*^ ACLF model mice treated with vehicle or CCS1477 (*n* = 6 biologically independent mice samples per group). **D** Immunofluorescence staining of DAPI (blue) and RIPK1 (red) in the liver of WT and *Acod1*^*Mye−/−*^ ACLF model mice treated with vehicle or CCS1477. Representative images (left panel) and quantification graph (right panel) (*n* = 6 biologically independent mice samples per group). Scale bars, 0.05 mm (upper panel) and 0.02 mm (lower panel). Serum ALT (**E**), TBIL (**F**), and blood bacterial loads (**G**) levels in WT and *Acod1*^*Mye−/−*^ ACLF model mice treated with vehicle or CCS1477 60 h post-CCl_4_ injection (*n* = 6 biologically independent mice samples per group). **H** Schematic of adoptive transfer of in vitro–induced SAMs from CD45.2 mice into CD45.1 ACLF mice. Created in BioRender. Yu, P. (2026) https://BioRender.com/6ol5ip0. **I** Survival analysis of ACLF model mice after adoptive transfer of SAMs from different groups or PBS (*n* = 8 biologically independent mice per group). **J** Blood bacterial loads in ACLF model mice after adoptive transfer of SAMs from different groups or PBS (*n* = 8 biologically independent mice sampples per group). Bars represent mean ± SD. In (**B**, **I**), survival curves were compared using the two-sided log-rank (Mantel–Cox) test. In (**D**–**G**, **J**), one-way ANOVA followed by Tukey’s multiple-comparisons test was used. VEH, vehicle; CCS, CCS1477; BMs, bone marrow cells; SAM-WT-CON, in vitro-induced SAMs from WT mouse bone marrow treated with DMSO; SAM-WT-CCS, in vitro-induced SAMs from WT mouse bone marrow treated with CCS1477; SAM-KO-CON, in vitro-induced SAMs from *Acod1*^*Mye−/−*^ mouse bone marrow treated with DMSO; SAM-KO-CCS, in vitro-induced SAMs from *Acod1*^*Mye−/−*^ mouse bone marrow treated with CCS1477. Source data are provided as a Source Data File.

To figure out how CCS1477 works in ACLF, we compared genes upregulated in SAMs in ACLF versus liver fibrosis with those downregulated in ACLF after CCS1477 treatment. We found 10 genes, notably *Acod1*, which was highly expressed in SAMs (Figs. 4A, S10G). *Acod1* depletion has been shown to regulate the innate immunity of monocytes and macrophages (Mono/Macs)^9^ and has a protective role in polymicrobial sepsis^26^. To determine whether CCS1477 alleviates ACLF through Acod1-mediated SAMs, we used *Acod1*^*Mye−/−*^ mice, which had conditionally depleted *Acod1* in myeloid cells, and subjected them to ACLF models with or without CCS1477. Similar to the therapeutic effects from CCS1477, *Acod1*^*Mye−/−*^ mice displayed prolonged survival compared with littermate controls (Fig. 4B). CCS1477 could not further improve the survival of *Acod1*^*Mye−/−*^ mice (Fig. 4B). SAMs in their livers were significantly reduced, similar to the effect of CCS1477 treatment, with no further reduction upon additional CCS1477 treatment (Fig. 4C). What’s more, CCS1477 did not further decrease liver damage or necroptosis in these mice, indicating its efficacy relies on Acod1 (Fig. 4D–F).

Considering that ACLF is a multi-stage model, we also evaluated the impact of *Acod1* KO during the chronic CCl_4_-induced fibrosis phase. *Acod1*^*Mye−/−*^ mice showed higher serum ALT and TBIL (Fig. S11A), more extensive liver fibrosis on Sirius Red staining (Fig. S11B), and increased hepatic α-SMA and COL3A1 expression compared with littermate controls (Fig. S11C). Flow cytometry revealed a higher proportion of SAM in liver (Fig. S11D). These findings indicate that myeloid-specific deletion of *Acod1* promotes liver fibrosis in CCl_4_-induced mice, suggesting that the therapeutic benefit of *Acod1* knockout in ACLF may occur primarily during the acute phase rather than the chronic fibrosis phase.

Since bacterial infections markedly exacerbate ACLF and the localization of SAMs suggests their involvement in hepatic antibacterial immunity, we hypothesized that CCS1477 may improve ACLF by enhancing SAM phagocytic activity via Acod1. Plasma bacterial cultures indicated that CCS1477 and the *Acod1*^*Mye−/−*^ group both showed reduced bacterial loads in ACLF models (Fig. 4G). However, CCS1477 did not further reduce bacterial load in *Acod1*^*Mye−/−*^ mice (Fig. 4G), indicating that the therapeutic efficacy of CCS1477 in ACLF is Acod1-dependent. To determine whether the reduced bacterial load was caused by the phagocytic capacity of SAMs, we isolated and induced bone marrow cells from *Acod1*^*Mye−/−*^ and wildtype (WT) mice into SAMs^13^ (Fig. S12A, S12B). We next assessed their phagocytic ability toward *Escherichia coli (E. coli)* particles under LPS and phorbol 12-myristate 13-acetate (PMA) stimulation, mimicking the hyperinflammatory and oxidative stress conditions in ACLF, respectively. Upon LPS or PMA stimulation, SAMs showed reduced phagocytic capacity, indicating impaired antibacterial function under ACLF-like conditions (Fig. S12C–S12D). CCS1477 treatment increased phagocytosis in WT SAMs (Fig. S12C). Similarly, *Acod1*^*Mye−/−*^ SAMs exhibited enhanced phagocytosis compared with WT under stimulation (Fig. S12D), indicating functional restoration. CCS1477 rescued phagocytosis in WT SAMs but had no additional effect in *Acod1*^*Mye−/−*^ SAMs (Fig. S12D), suggesting an Acod1-dependent mechanism. Collectively, these data suggest that SAMs exhibit impaired antibacterial immunity under ACLF-like hyperinflammatory or oxidative stress conditions, and that CCS1477 alleviates ACLF by reprogramming SAMs to enhance their phagocytic capacity mediated by Acod1.

To further confirm that EP300 inhibition ameliorates ACLF primarily by enhancing the antibacterial function of SAMs, we performed SAM adoptive-transfer experiments in ACLF mice (Fig. 4H). Flow cytometry showed that the hepatic abundance of transferred CD45.2^+^ cells was comparable across groups (Fig. S12E). Mice receiving CCS1477-treated SAMs from WT mice or untreated SAMs from *Acod1*^*Mye−/−*^ mice showed a reduced proportion of CD45.2^+^ SAMs, whereas CCS1477 treatment of *Acod1*^*Mye−/−*^ donor cells did not further decrease this proportion (Fig. S12E). ACLF mice receiving in vitro-induced SAMs from WT mice exhibited prolonged survival and reduced blood bacterial load compared with mice receiving PBS (Fig. 4I, 4J), indicating that in vitro–induced SAMs possess antibacterial activity, consistent with our previous in vitro phagocytosis assays (Fig. S12C). ACLF mice receiving CCS1477-treated SAMs from WT mice exhibited further prolonged survival and reduced bacterial load compared with mice receiving CCS1477-untreated SAMs (Fig. 4I, 4J). Similarly, ACLF mice receiving untreated SAMs from *Acod1*^*Mye−/−*^ mice showed comparable survival and bacterial load, and CCS1477 treatment did not further improve these outcomes (Fig. 4I, 4J). These results indicate that CCS1477 alleviates ACLF by enhancing the antibacterial function of SAMs, and its efficacy depends on Acod1.

We then explored whether SAM-derived cytokines participate in ACLF progression and therapeutic treatment. Single-cell transcriptomic analysis of human samples showed that SAMs expressed *TNF*, *IL1B*, *IL6*, *IL10,* and *TGFB1* without notable differences between LC and ACLF patients (Fig. S13A, S13B). Similar results were seen in mice when comparing SAMs from fibrosis and ACLF models (Fig. S13C, S13D). Flow cytometry also confirmed that SAM secretion of TNF-α, IL-6, and IL-10 was comparable between fibrosis and ACLF mice (Fig. S13E). These findings indicate that cytokine production by SAMs is not a major contributing factor in ACLF progression.

### CCS1477 transcriptionally represses Acod1 expression

**Fig. 5:**
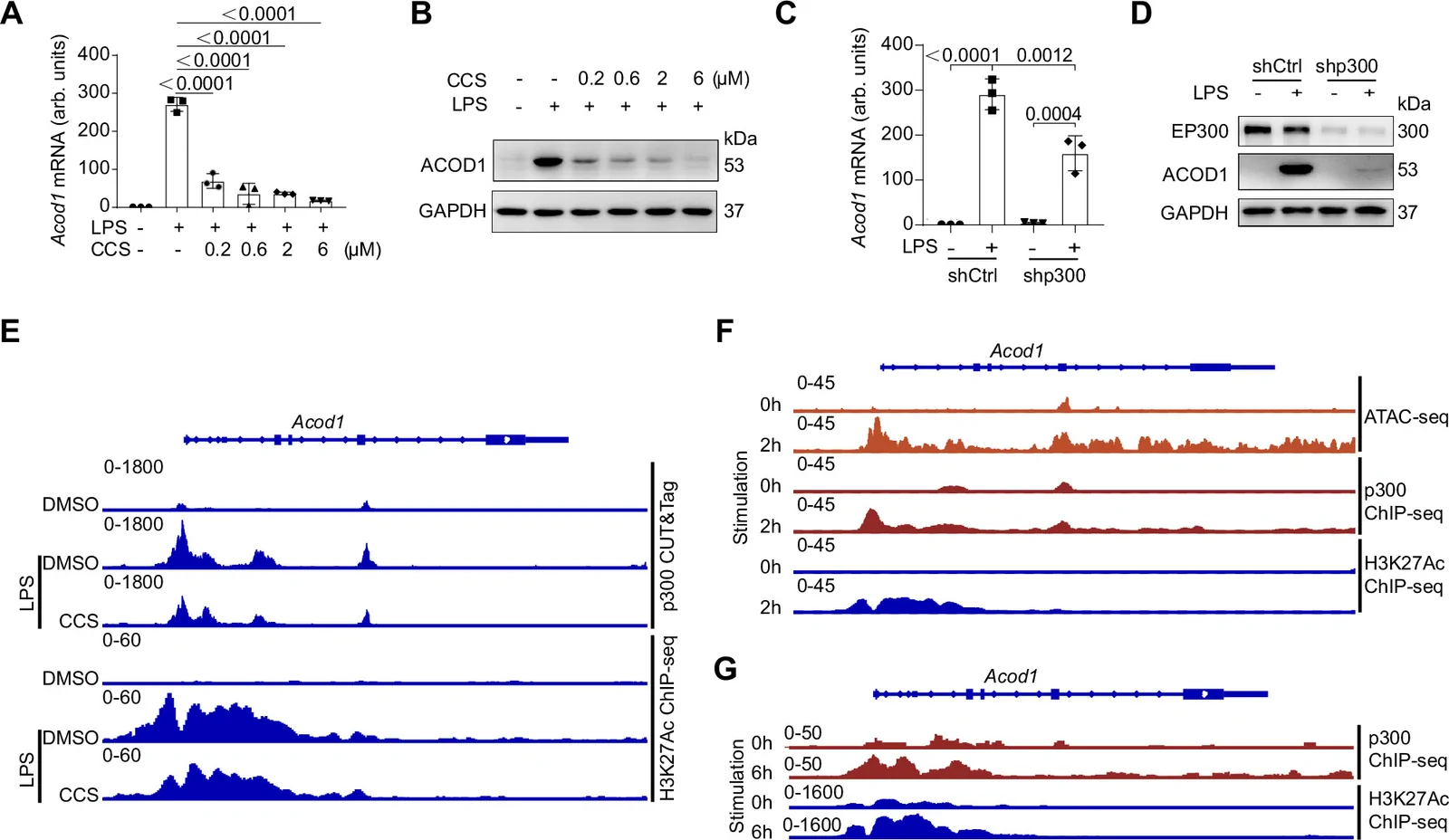
CCS1477 transcriptionally represses Acod1 expression. **A** RT-qPCR shows mRNA expression levels of *Acod1* before and after LPS stimulation with or without CCS1477 treatment in RAW264.7 cells (*n* = 3 biologically independent experiments). Comparisons were made between the second group and the other treatment groups. **B** Western blot analysis reveals protein levels of ACOD1 before and after LPS stimulation with or without CCS1477 treatment in RAW264.7 cells (*n* = 3 biologically independent experiments). **C** RT-qPCR shows mRNA expression levels of *Acod1* before and after *Ep300* knockdown with or without LPS stimulation in RAW264.7 cells (*n* = 3 biologically independent experiments). **D** Western blot analysis reveals protein levels of ACOD1 before and after *Ep300* knockdown with or without LPS stimulation in RAW264.7 cells (*n* = 3 biologically independent experiments). **E** Genome browser views of p300 (upper panel, using CUT &Tag) and H3K27ac (lower panel, using ChIP-seq) at *Acod1* before and after LPS stimulation, with and without CCS1477 treatment in RAW264.7 cells. Genome browser views of chromatin accessibilities (**F** upper panel, using ATAC-seq), p300 (**F** middle panel and **G** upper panel, using ChIP-seq), and H3K27ac (**F** lower panel and **G** lower panel, using ChIP-seq) at *Acod1* after stimulation at 0 h, 2 h and 6 h in mice BMDMs. Bars represent mean ± SD. In (**A**, **C**), one-way ANOVA followed by Tukey’s multiple-comparisons test was used. CCS, CCS1477; arb. units, arbitrary units. Source data are provided as a Source Data File.

CCS1477 selectively targets the bromodomain of p300/CBP, thereby modulating histone acetylation and transcriptional activity by reducing p300 and CBP recruitment to target genes^22,23^. To determine whether p300/CBP regulates Acod1 expression in response to bacterial infections, we stimulated mouse macrophage RAW264.7 cells with LPS^27^. CCS1477 dose-dependently lowered *Acod1* mRNA *and ACOD1 protein* levels (Fig. 5A, 5B). Similarly, *Ep300* knockdown reduced Acod1 mRNA and ACOD1 protein expression in LPS-activated macrophages (Fig. 5C, 5D).

H3K27Ac is an epigenetic modification associated with transcriptional activation, predominantly regulated by p300/CBP^28–30^. To investigate whether the decreased expression of *Acod1* by CCS1477 treatment was caused by reduced recruitment of p300/CBP, we conducted p300 Cleavage Under Targets and Tagmentation (CUT&Tag) and H3K27Ac Chromatin Immunoprecipitation Sequencing (ChIP-seq) in LPS-stimulated macrophages, with or without CCS1477 treatment. Upon LPS stimulation, we observed a substantial increase in p300 occupancy and increased surrounding H3K27Ac at the *Acod1* promoter region, whereas CCS1477 treatment demonstrated significant reductions (Fig. 5E). Similarly, analysis of the publicly available datasets from PAMP-stimulated mouse bone marrow-derived macrophages (BMDMs) revealed increased Ep300 binding, elevated H3K27Ac marks, and an open chromatin state at the promoter of *Acod1* (Fig. 5F, 5G). Overall, these findings suggest that CCS1477 transcriptionally represses the expression of *Acod1* at its promoter region.

### ACOD1 expression is upregulated in SAMs from ACLF patients and correlates with poor prognosis

**Fig. 6:**
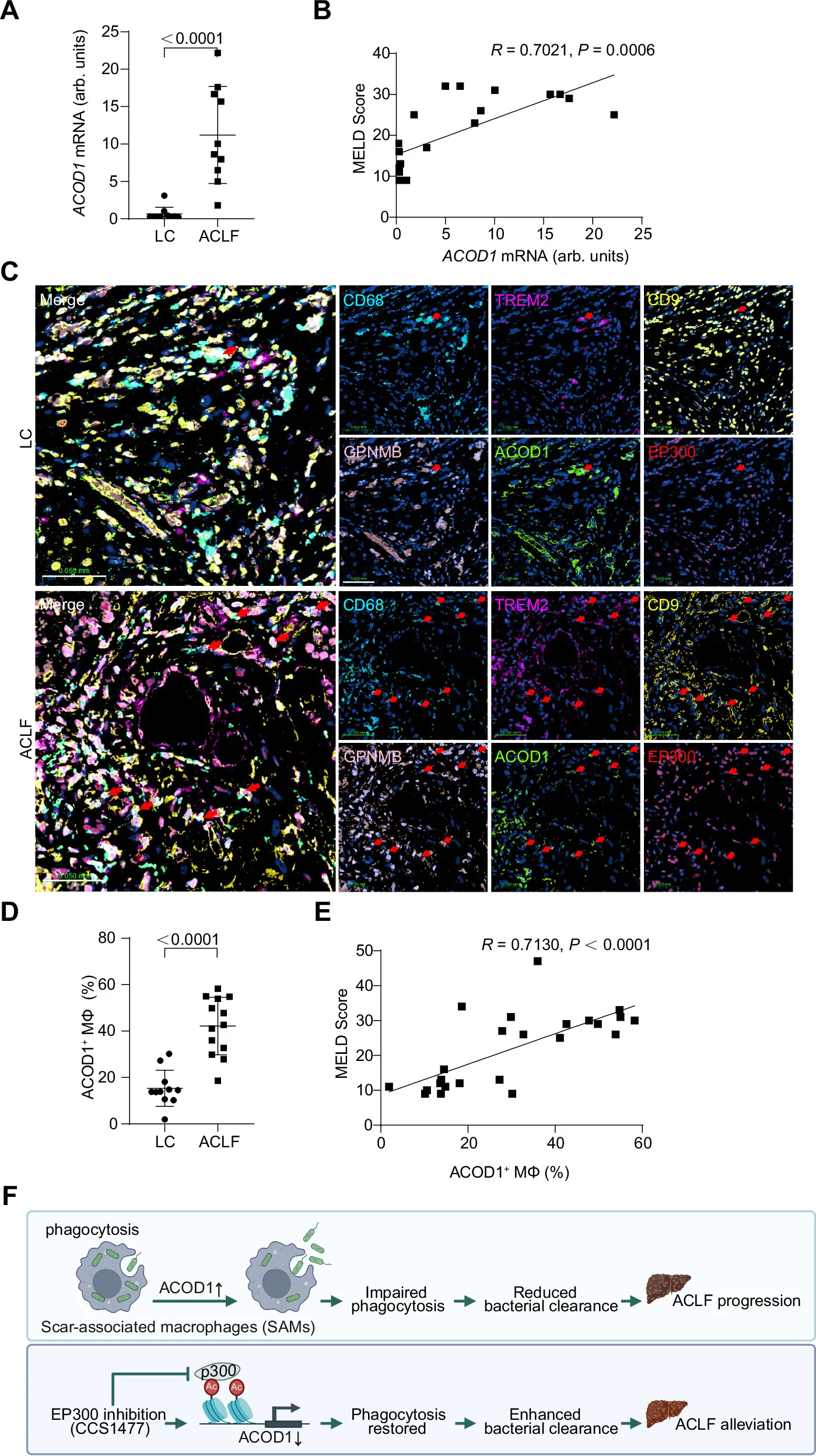
ACOD1 expression in macrophages is associated with the severity of ACLF in patients. RT-qPCR shows mRNA expression levels of *ACOD1* in the LMCs of LC and ACLF patients (*n* = 10 biologically independent human samples per group) (**A**) and its correlation with MELD score (**B**). **C** Representative mIHC images of SAMs (CD68^+^TREM2^+^CD9^+^GPNMB^+^, red arrows) in the livers of LC and ACLF patients and co-localized with ACOD1 and EP300 (*n* = 11 for LC and *n* = 13 for ACLF, biologically independent human samples). Scale bars, 0.05 mm. Quantification of ACOD1^+^ macrophages (CD68^+^ACOD1^+^) proportion among all macrophages (CD68^+^) in the field of view (five fields per patient) (*n* = 11 for LC and *n* = 13 for ACLF,  biologically independent human samples) (**D**) and its correlation with MELD score (**E**). **F** EP300-ACOD1 regulation of SAM phagocytosis in ACLF. Created in BioRender. Yu, P. (2026) https://BioRender.com/ucqqgk6. Bars represent mean ± SD. In (**A**), a two-sided Mann-Whitney U test was used. In (**B**, **E**), correlation was assessed using Spearman rank correlation. In (**D**), a two-sided unpaired Student’s *t* test was used. MΦ, macrophages; arb. units, arbitrary units. Source data are provided as a Source Data File.

To assess whether ACOD1 is specifically induced in ACLF SAMs, we first used quantitative real-time PCR (RT-qPCR) to measure *ACOD1* transcription in liver mononuclear cells (LMCs) from LC and ACLF patients. *ACOD1* expression was significantly higher in ACLF LMCs and linked to poor prognosis (Fig. 6A, 6B). In addition, mIHC showed that ACOD1 colocalized with EP300 and SAMs in ACLF livers, increasing compared to LC, further suggesting that ACOD1 is regulated by EP300 in ACLF SAMs (Fig. 6C). Moreover, compared to cirrhotic livers, the number of ACOD1^+^ macrophages in the liver of ACLF patients was significantly increased and positively correlated with poor prognosis (Fig. 6D, 6E). Together, these data indicate that the expression of ACOD1 is specifically induced in ACLF SAMs and is associated with poor prognosis in ACLF patients.

## Discussion

Recent studies have identified that SAMs share markers such as TREM2 and CD9 with lipid-associated macrophages and non-alcoholic steatohepatitis (NASH)-associated macrophages, suggesting they likely belong to the same cell group^13,14^. SAMs are linked to the severity of various liver diseases^13,14,31,32^, highlighting the need for targeted treatments. However, the immunobiology and function of SAMs in ACLF remain unclear. This study shows that SAMs increase in both the liver and PBMCs during HBV-related ACLF, indicating their potential as diagnostic biomarkers. This increase, with consistent periportal/scar localization, occurs in both HBV- and alcohol-related ACLF, suggesting it’s intrinsic to ACLF regardless of cause. SAMs infiltrate portal areas and show reduced bacterial clearance ability, remaining concentrated around portal/scar regions despite different necrosis patterns. This expansion likely compensates for Kupffer cell loss^33–35^, aiming to maintain antibacterial defense.

Single-cell transcriptomic sequencing aids in revealing transcriptional regulation in specific cell subtypes. We identified that six TFs commonly regulate SAMs in both humans and mice, but the key TF among them is still unknown. EP300 and ELF1 are promising candidates for regulating these TFs. Notably, ELF1 has limited research and no known inhibitors. EP300, a key co-transcriptional regulator with epigenetic functions, significantly alleviated ACLF symptoms in our study. Despite common side effects, epigenetic drugs’ potent efficacy allows for single-dose treatments, crucial for high short-term mortality diseases like ACLF. We observed that CCS1477 did not materially change CCR5^+^CXCR3^+^, CXCR2^+^CD9^+^, or CCR3^+^ macrophage proportions, arguing against a broad redistribution across MPs, identifying SAMs as the key effector of EP300 inhibition in ACLF. Nevertheless, effects on parenchymal or barrier tissues cannot be excluded; prior work indicates that EP300/CBP modulation can influence hepatocytes and intestinal epithelial cells^36^. Whether EP300 inhibition affects gut epithelial cells and thereby modulates bacterial translocation to the liver represents an interesting research direction that warrants further investigation in the future. Crucially, our in vitro phagocytosis assays demonstrated that CCS1477 restores the impaired bacterial clearance of SAMs. Moreover, adoptive-transfer experiments show that CCS1477-conditioned SAMs restore bacterial clearance and improve outcomes in recipient mice, indicating that enhancement of SAM antibacterial function is a principal mediator of benefit in ACLF, even if ancillary EP300-dependent effects in other tissues also may contribute. In addition, by comparing cytokine production by SAMs between ACLF and LC/fibrosis, we confirmed that cytokine secretion by SAMs is not a key factor driving ACLF progression.

ACOD1 is highly induced in macrophages during inflammation, playing an essential role in immunometabolism and exerting context-dependent effects^15,16,27,37^. Prior work in sterile inflammation like ischemia-reperfusion injury, ACOD1 generally exerts protective effects by suppressing excessive inflammation^38^, while in LPS-tolerized monocytes and endotoxemia contexts emphasized ACOD1-driven itaconate in enforcing hyporesponsiveness, which can be reversed by β-glucan–mediated ACOD1 reduction and restoration of SDH activity^27^. However, bacteria infection-aggravated ACLF constitutes a distinct inflammatory and metabolic milieu, warranting a phase- and context-aware interpretation. We chose a *K. pneumoniae*-aggravated ACLF mouse model and an in vitro SAMs phagocytosis model using *E. coli*, in which LPS and PMA were applied to simulate the high-inflammatory or oxidative stress conditions observed in ACLF patients. In this setting, excess ACOD1 in SAMs compromised bacterial clearance, whereas ACOD1 attenuation partially restored phagocytosis, reduced bacterial burden, and alleviated disease severity. In the chronic CCl_4_-induced fibrosis stage, increased SAMs appear primarily reactive rather than driven by myeloid *Acod1*, and *Acod1* deletion exacerbates fibrosis. Thus, the therapeutic benefit of ACOD1 attenuation or EP300 inhibition manifests predominantly during the acute infectious phase, when ACOD1 overexpression drives SAM immunoparalysis. Together, these observations indicate that neither ACOD1 nor SAMs are intrinsically disease-promoting; rather, context and magnitude determine their net effect—anti-inflammatory/pro-repair features can prevail in sterile injury, while overexpression during infection imposes immunoparalysis that blunts SAM antibacterial programs. However, whether ACOD1’s contribution in ACLF is strictly itaconate-dependent remains to be determined.

Our study has certain limitations. Future research should employ time- and cell-specific SAM depletion models in ACLF progression. We lack direct evidence that infiltrating monocytes are the main SAM precursors influenced by ACOD1, and future fate-tracing and xenograft experiments could clarify this. In addition, the spatial distribution of SAMs and their interactions with other cell types cannot be precisely resolved by our current mIHC data, and further validation using orthogonal spatial approaches such as spatial transcriptomics or multiplexed imaging is needed. Our animal models and conclusions primarily simulate infection-aggravated ACLF patients, and further studies are required to determine whether these findings apply to ACLF without infection, such as alcohol- or drug-induced ACLF. Additionally, SAM’s other functions are also awaiting broader research. Finally, incomplete clinical metadata in external datasets, including unavailable variables such as sample sex, may introduce bias in the transcriptomic analyses.

In this work, we found that more immune-suppressive SAMs in ACLF patients are associated with disease progression due to reduced bacterial clearance. Increased SAM abundance correlates with worse patient outcomes. Elevated ACOD1 impairs SAMs’ bacterial clearance, while EP300 or ACOD1 inhibition mitigates hepatic phenotypes and restores bacterial clearance (Fig.6F and Fig.S14). These results highlight the role of immune suppression in ACLF and suggest that strategies aimed at restoring immune function, including EP300 or ACOD1 inhibition, may provide promising therapeutic options. Our study further supports the development of small-molecule inhibitors targeting ACOD1 or epigenetic modulators that can reprogram immune cell function.

## Methods

### Patients and study samples

All participants in this study were recruited from Shanghai Renji Hospital, and written informed consent was obtained from each participant. The study adhered to the principles of the Declaration of Helsinki and was approved by the Renji Hospital Ethics Committee, Shanghai Jiao Tong University School of Medicine (approval numbers KY2020-055 and KY2021-063-B). Participants were recruited according to the predefined inclusion and exclusion criteria. No self-selection bias was identified. No participant compensation was provided. All human tissue samples used in this study were fully processed and consumed during the analyses.

Sex was recorded from the participants’ medical records and was not considered as a biological variable in the study design or statistical analyses. Therefore, the cohort included both male and female patients. Detailed demographic and clinical characteristics, including age and sex, are provided in Supplementary Tables S1–S3. Exclusion criteria for the study included the presence of hepatocellular carcinoma and/or other tumors, immune system-related diseases such as autoimmune hepatitis (AIH) and rheumatism, hepatitis caused by alcohol, drugs, or other viruses, and the use of glucocorticoids or immunosuppressive drugs.

Human PBMCs were isolated from freshly obtained blood by gradient centrifugation on Ficoll Hypaque Plus (GE Healthcare), then frozen in fetal bovine serum (FBS, Gibco, 10091-148) with 10% DMSO. PBMCs from patients with LC (*n* = 34) and ACLF (*n* = 27) were isolated to examine the percentage of TREM2^+^CD9^+^ subset among CD11B^+^CD14^+^CD68^+^ cells using flow cytometry. Detailed demographic and clinical features of these subjects are listed in Table S1.

Liver samples were obtained from individuals aged over 18 who underwent liver biopsy or liver transplantation at Renji Hospital, Shanghai Jiao Tong University School of Medicine. Participants were assigned to groups based on their clinical diagnosis. A total of 34 human liver tissues were used in this study. The characteristics of HBV infection were determined by measuring the patient’s HBV DNA titer and conducting tests for HBV antigens and antibodies. Liver tissues used in this study consisted of residual biopsy specimens collected during liver transplantation procedures. The diagnosis of LC (*n* = 16) was based on previous liver biopsy results and clinical evidence. The ACLF patients (*n* = 18) met the diagnostic criteria of both the European Association for the Study of the Liver-Chronic Liver Failure Consortium (EASL-CLIF) and the Chinese Group on the Study of Severe Hepatitis B-ACLF (COSSH-ACLF) definitions. Demographic and clinical features of these subjects are detailed in Tables S2–S3.

### Human LMCs isolation

Liver resection tissues were obtained from patients with LC or ACLF who underwent liver transplantation and healthy liver donors. About 2–3 × 10^5^ cells/g were isolated from the healthy liver. Due to severe liver dysfunction and failure, about 0.5-1 × 10^5^ cells/g were isolated from the LC or ACLF liver. Briefly, excised tissues were immediately placed in Roswell Park Memorial Institute Medium 1640 (RPMI 1640, Gibco, Carlsbad, CA, USA, C22400500) on ice and transported to the laboratory within 30 min. Tissues were minced with scissors on ice and digested with 0.01% collagenase IV (Sigma-Aldrich, St. Louis, CA, USA, C4-22-1G) at 37 °C for 30 min with agitation (220 RPM), then strained through a 70 µm cell strainer along with RPMI 1640 medium. Then cell suspensions were gently centrifuged at 50 g for 5 min; the cell pellet was removed, and the supernatant was centrifuged at 500 × *g* for 10 min. Thereafter, the cell pellet was resuspended in 35% Percoll (Cytiva, Uppsala, Sweden, 17089109) and centrifuged at 900 × *g* for 30 min without brake. Red blood cell lysis was performed, followed by removing the top fragment. The lysis procedure was stopped with RPMI 1640 medium containing 10% FBS and centrifuged at 500 g for 5 min. After removing the supernatant, the cell pellet was resuspended and strained through a 30 µm cell strainer (Miltenyi Biotec, Bergisch Gladbach, Germany, 130-098-458) to remove remaining debris. Then, LMCs from patients were separated and stored in FBS with 10% DMSO. LMCs were isolated from liver explants of LC (*n* = 10) and ACLF (*n* = 10) patients for mRNA extraction. Demographic and clinical features of these subjects are provided in Table S3.

### ACLF models, treatments, and study samples

C57BL/6 J WT mice were obtained from Beijing HFK Bio-Technology Co., Ltd. *Lyz2*-Cre and C57BL/6J-*Acod1*^*em1Cflox*^ mice were obtained from Cyagen Biosciences (Suzhou) Inc., used to create conditional knockout mice for *Acod1* in myeloid cells. All animal experiments were approved by the Institutional Animal Care and Use Committee (IACUC) of Shanghai Institute of Materia Medica, Chinese Academy of Sciences (approval No. 2022-06-LC-29, 2023-06-LC-35, 2024-06-LC-42, 2024-03-LC-39, 2025-08-LC1-59 and 2025-08-LC1-60) and were performed in accordance with institutional guidelines. Briefly, the mice were housed in a specific pathogen-free facility under controlled environmental conditions: temperature 22–24 °C, relative humidity 50-60%, and a 12-h light/dark cycle. At the end of the experiments, mice were humanely sacrificed by carbon dioxide inhalation followed by cervical dislocation.

Eight-week-old male mice (C57BL/6 J) were used to establish the liver fibrosis and ACLF model as described previously^20^. Briefly, liver fibrosis was induced by intraperitoneal injection of carbon tetrachloride (CCl_4_) diluted in olive oil (0.2 mL/kg, twice weekly) for 8 weeks. After establishment of the liver fibrosis model, mice were stratified according to body weight to ensure comparable mean body weight among groups and were then randomly assigned to experimental groups using a random number table. ACLF was subsequently induced in fibrotic mice by an additional acute intraperitoneal double dose of CCl_4_ (0.4 mL/kg), followed by intraperitoneal administration of *Klebsiella pneumoniae* strain 43816 (ATCC). Mice in the treatment group received CCS1477 by intraperitoneal injection 2 h before bacterial challenge, whereas control mice received the corresponding vehicle. Mice were euthanized 36 h after *Klebsiella pneumoniae* administration, and blood and liver tissues were collected for subsequent analyses. Fibrosis control mice received chronic CCl4 treatment alone and were euthanized at the corresponding experimental time point. Blood and liver tissues were collected for subsequent analyses.Mice were also intraperitoneally treated with CCS1477 (10 mg/kg, MCE #HY-111784), NEO2734 (5 mg/kg, MCE #HY-136938), GNE207 (10 mg/kg, MCE #HY-120028), imipenem (25 mg/kg, MCE #HY-B1369A) or specific vehicle solvent. The solvent for imipenem is phosphate-buffered saline (PBS), while the solvents for other drugs are 10% DMSO, 30% polyethylene glycol 300 (PEG300), and 60% PBS.

### Mouse liver sample preparation and scRNA-seq analysis

Livers were retrograde perfused for 3 min at a rate of 5-7 ml/min through the inferior vena cava using HBSS without Ca^2+^ or Mg^2+^ supplemented with 0.5 mM EGTA, 0.5 mM EDTA, and 20 mM HEPES. Perfusions were then switched to 40 ml of a digestion buffer, held at 37 °C, consisting of HBSS with Ca^2+^ and Mg^2+^ supplemented with 0.033 mg/ml of Liberase TM (Roche), 20 µg/ml DNaseI (Sigma), and 20 mM HEPES. Livers were then excised, minced, and digested in vitro for an additional 20 min at 37 °C with gentle rotation in 20 ml of fresh digestion buffer. The perfusion and digestion steps were performed in the presence of 1 µM flavopiridol to offset transcriptional changes associated with digestion. After tissue digestion, cells were passed through a 70-micron cell strainer, and hepatocytes removed by two low-speed centrifugation steps at 50 g for 2 min. NPCs in the supernatant were further separated from debris by pelleting for 15 min at 900 g in 50 ml of 35% Percoll (GE, Pharmacia) at room temperature. Cells were then washed from Percoll-containing buffer and subjected to isotonic erythrocyte lysis. Enriched NPCs were washed, suspended in PBS, and stained for 15 min with BD Horizon™ Fixable Viability Stain 780 to label dead cells. Cells were then immunolabeled with specific antibodies of interest, washed, and sorted using a Beckman Coulter MoFlo Astrios EQ. Liver NPCs were sorted using Fluorescence-Activated Cell Sorting (FACS) based on CD45^+^ and viability (negative for the live/dead dye Fixable Viability Stain 780). The resulting purified cell preparations were washed twice with PBS, suspended in 1 ml of RPMI 1640 medium containing 2% FBS, and filtered through a 30 μm cell strainer. The concentration of the cell suspension was adjusted to 600-1200 cells/μl. The single-cell suspension was then kept on ice before loading into a BD Rhapsody cartridge for single-cell capture.

### Single-cell capturation, library preparation, and sequencing

Cells were loaded into a BD Rhapsody micro-well cartridge, followed by capture beads loading excessively. Then, redundant beads were removed. After that, lysis buffer with DTT was added for cell lysis. 2 min after adding lysis buffer, Cell capture beads were retrieved and washed prior to performing reverse transcription. Single-cell transcriptome with cell labels and unique molecular identifiers (UMI) was generated into a cDNA library using the BD Rhapsody WTA Amplification Kit (BD Biosciences, Cat. No. 633801) according to the manufacturer’s protocol.

Sequencing was performed on Illumina NovaSeq 6000 with PE150 read length at Chi-Biotech Co., Ltd.

### Acquisition of scRNA-seq data

For scRNA-seq data of humans, 15 human liver samples from two studies were applied in this work. Specifically, the processed matrix file and its corresponding metadata of HBV patients were downloaded from the GEO database of NCBI (GEO Series: GSE216314). The remaining scRNA-seq data were obtained from https://www.ncbi.nlm.nih.gov/sra/PRJNA913603 via “prefetch” command in Linux. The downloaded SRA files were then converted to FASTQ format using the “fasterq-dump” command. Unlike the 10x Genomics platform, the two FASTQ files obtained from each SRA file were transformed into corresponding expression matrix files using the celescope command.

For scRNA-seq data of mouse, the quality of the raw reads was assessed using fastp. Raw sequencing reads of the cDNA library were processed through the BD Rhapsody^TM^ Sequence Analysis Pipeline (v2.0), which included filtering by read quality, annotating reads, annotating molecules, determining putative cells, and generating a single-cell expression matrix. Among all the output files, the matrix of UMI counts for each gene per cell was used for downstream analysis.

### Preprocessing of scRNA-seq data

High-quality cells were preserved by applying the following exclusion criteria: (1) total UMI counts of no more than 1,000; (2) gene numbers no higher than 500 or no less than 10,000; (3) mitochondrial gene percentage greater than 20%. The expression levels of each gene in each cell were normalized using the “NormalizeData” function with the default parameters, while those genes which was expressed less than 3 cells was excluded before. Each sample underwent the following steps: “FindVariableFeatures” with 2,000 genes, “ScaleData”, “RunPCA”, “FindNeighbors” with the first 20 PCs, and “FindClusters” with a resolution of 1. All samples were merged into a single object using the “merge” function of Seurat v4.3.0.1 in R v4.3.0, and batch effects were corrected using “RunHarmony” from the harmony v0.1.1 R package.

### Annotation of scRNA-seq data

Each cluster was renamed manually based on the marker genes, which were defined by utilizing the “FindAllMarkers” function (LogFC > 0.5, min.pct > 0.25). We extracted MP for further clustering using the “FindNeighbors” function with the first 15 PCs and “FindClusters” function at a resolution of 0.5 (the resolution of mouse data was 0.3).

### Pseudotime and trajectory visualization

For pseudotime and lineage trajectories of MPs, we firstly applied the “as.cell_data_set” function of SeuratWrappers v0.3.1 R package to convert the pre-processed Seurat object into a “cds” object. Then “cluster_cells”, “learn_graph” and “order_cells” function of monocle3 v1.3.4 R package were utilized to infer the lineage trajectories. “CytoTRACE” function of CytoTRACE v0.3.3 R package was also applied to validate the trajectory. To firmly concern the source of the MPs, we employed the “velocyto” command in Linux to process the barcode and BAM files generated during the celescope process. Additionally, the reference files for velocyto included “hg38_rmsk.gtf” and “genes.gtf”; the former was downloaded from the UCSC Table Browser, and the latter was from 10x Genomics official website.

### Regulon construction

SCENIC v1.3.1 R package was utilized to infer main regulons for each cluster of MPs. We constructed a gene expression matrix from 1500 randomly selected cells both due to the limitations of computational memory and recommendations of SCENIC official (MP0-3, 5-6, 8 were extracted from human data due to their lineage relationships, while all MPs from mouse data were used). The “geneFiltering” function was applied to select genes for downstream analysis with the following parameter settings: minCountsPerGene = 3 ∗ 0.01 ∗ ncol (exprMat), minSamples = ncol (exprMat) ∗ 0.01). The downstream pipeline for constructing gene regulatory networks was based on “runCorrelation”, “runGenie3” with setting “nParts = 20”,”runSCENIC_1_coexNetwork2modules”, and”runSCENIC_2_createRegulons”. Following this, the entire gene matrix of MPs was used for “runSCENIC_3_scoreCells” and “runSCENIC_4_aucell_binarize”.

### Detection of major regulon in ACLF disease

“BEAM” function from the Monocle v2.28.0 R package was applied for branch analysis of MP0, 1, 5 in human scRNA-seq data, and genes whose *p* value lower than 0.05 were reserved. Then, those TFs in SCENIC results whose regulon activity was higher than 0 in at least one cluster of MP0, 1, 5 were made intersection with above reserved genes. As we didn’t find a similar cluster of MP5 in mouse data, we chose TFs in mouse SCENIC results whose regulon activity was higher than 0 in at least one cluster of MP0, 4 (corresponds to MP0, 1 in human data) and described their relationship to the trajectory using “plot_pseudotime_heatmap” function. Next, we made an intersection of TFs that were specifically upregulated with trajectories from MP0 to MP1 (MP0 to MP4 in mouse data) in human and mouse data, and obtained 6 TFs (NFIC, NR1H3, MAF, MAFB, MITF, MAFF). We predicted TFs upstream of these 6 TFs using mouse SCENIC data and plotted them using Cytoscape v3.10.1 software.

### Bulk RNA-seq data processing

Previously published human liver bulk RNA-seq data from healthy controls (HC, *n* = 14), liver cirrhosis (LC, *n* = 17), and acute-on-chronic liver failure (ACLF, *n* = 18) were reanalyzed in the present study^39^. Briefly, the liver tissues were previously collected and stored frozen at −80 °C. After cutting 50 mg of each sample in liquid nitrogen, the tissue RNA was extracted using grinding beads and the FastPure® Cell/Tissue Total RNA Isolation Kit V2 (Vazyme, China). After the extraction of RNA, its purity was assessed using a NanoDrop by measuring OD_260/280_ and OD_260/230_ ratios, and its integrity was evaluated using Agilent 2100/4150. Once the RNA quality was checked and confirmed, total RNA from human liver tissues was used to prepare cDNA libraries, which were subsequently sequenced on a NovaSeq6000 platform (Chi-Biotech Co., Ltd.) using paired-end methods. The sequencing reads were mapped to Genome Reference Consortium Human Build 38 (GRCh38) using STAR v2.7.10b, and featureCounts software was used to quantify gene expression.

### GSVA analysis

We constructed a SAM gene set comprising 13 genes (FABP5P7, GPNMB, APOC1, ACP5, TREM2, SPP1, PLA2G7, FTLP2, MGLL, CD9, A2M, CXCL2, FTH1P7) based on the specificity criteria of SAMs in human scRNA-seq data (avg_log2FC > 1, pct.1 > 0.5, pct.2 < 0.3). The “gsva” function from the GSVA v1.48.3 R package was then used to examine the increase of SAMs in publicly available bulk RNA-seq data of ACLF (SRA Series: SRP201023; GEO Series: GSE168048, GSE180014, GSE139602).

### Mouse serum assay and liver function analyses

ALT and TBIL levels were measured in animals to evaluate liver injury on a Hitachi 7020 automatic analyzer (Hitachi, Tokyo, Japan) according to the manufacturer’s instructions.

### Peripheral blood bacterial counts

EDTA anti-coagulated peripheral blood was collected at the assigned time points after infection, serially diluted in sterile PBS, and grown overnight at 37 °C on Mueller-Hinton agar plates (Difco Laboratories). The number of viable bacterial colonies was determined by counting colony-forming units (CFU) and expressed as CFU/ml of blood.

### Measurement of liver and kidney microcirculation

The laser speckle contrast method was applied to measure the microcirculation of the liver and kidney as described previously^40^. Briefly, mice were anesthetized by inhalation of isoflurane via a nosecone. Body temperature was measured and maintained at 37 °C on a heating pad. The liver and kidney were exposed and scanned using a MoorFLPI-2 laser speckle contrast imager (Moor Instruments, Wilmington, DE). Microcirculation was assessed with the software provided by Moor Instruments.

### Histological analyses

Liver sections were embedded in paraffin and then stained with H&E and Masson trichrome to visualize the pattern of liver injury and fibrosis formation (Shanghai Runnerbio Technology). The histological features of the tissues were observed under a light microscope (Olympus, Tokyo, Japan) and imaged.

### Multiplex immunohistochemistry analysis

The detailed clinicopathological characteristics of the HBV-related patient cohort are summarized in Supplementary Table S2. Antibody information is provided in Supplementary Table S4.

### Cell Culture

Cell lines RAW264.7 were cultured in Dulbecco’s Modified Eagle Medium (DMEM) with 10% heat-inactivated fetal bovine serum. For stimulation, cells were pre-treatment with CCS1477 for 2 h, followed by treatment with LPS with or without CCS1477 for 6 h for subsequent RT-qPCR, Western blot, CUT & Tag, and ChIP-seq analysis. 293T cells were cultured in DMEM media with 10% FBS. All cells were cultured at 37 °C, 5% CO_2_, with complete media, and only exponentially growing cultures were used for assays. All cell lines were authenticated by short tandem repeat profiling and confirmed to be free of mycoplasma contamination using a PCR-based detection kit.

### shRNA construct and transfection

Ep300 knockdown was performed as previously described^41^. Briefly, Ep300 shRNA sequences and a control shRNA were integrated into the lentiviral vector pLent-U6-GFP-Puro (Vigenebio, China), and the constructed shRNA- pLent-U6-GFP-Puro plasmid was transfected into 293 T cells. The supernatant was collected and filtered at 48 and 72 h after transfection. RAW264.7 cells were infected with shRNA lentiviral particles in the presence of 10 μg/ml polybrene. After two days of puromycin selection, RAW264.7 cells were collected for subsequent experiments. Primer sequences for *Ep300* knockdown are presented in Table S4.

### RNA extraction and quantitative real-time polymerase chain reaction (RT-qPCR)

FastPure®Cell/Tissue Total RNA Isolation Kit V2 (Vazyme, China) was used to isolate the total RNA from cells, and RNA was converted into cDNA with a special cDNA synthesis kit (Vazyme, China) according to the manufacturer’s protocol. Gene expression was measured using quantitative RT-qPCR on a QuantStudio 6 Flex Real-Time PCR system (ABI). Expression of target genes was normalized with *GAPDH* and calculated using ∆∆Ct method. The primer sequences are listed in Table S4.

### Western blot analysis

Cell and tissue lysates were subjected to electrophoresis through SDS-PAGE and transferred to Nitrocellulose (NC) or polyvinylidene difluoride (PVDF) membranes, and then blotted with antibodies. Target protein bands were visualized using the enhanced chemiluminescence method in a ChemiScope3400 imaging system using ECL substrate (Clinx). Primary antibodies used are listed in the antibodies section of the supplementary materials. Uncropped and unprocessed scans of all immunoblots are provided in the Source Data file. Antibody information is provided in Supplementary Table S4.

### ChIP-seq and data analysis

ChIP was conducted following the manufacturer’s protocol (9005, Absin). Briefly, cells were cross-linked with 1% formaldehyde at room temperature for 10 min, which was terminated by Glycine Solution. After centrifugation, the acquired pellet was lysed by the indicated lysis buffer with a protease inhibitor cocktail. Then the extracted genomic DNA was digested enzymatically to achieve DNA fragmentation using micrococcal nuclease. Transfer 5 μl of the supernatant containing the digested chromatin to a 1.5 ml tube and store at −20 °C as the 5% total input sample from one ChIP. Then the samples were subjected to immunoprecipitation at 4 °C overnight with anti-H3K27ac. Add 20 μl ChIP Grade Protein A/G Plus Agarose to each IP and incubate for 1 h at 4 °C on a rocking platform. After resin incubation, the precipitated protein-DNA complexes were eluted, reversal of crosslinking, DNA was cleaned up, and prepared for subsequent sequencing (DIATRE Biotechnology). The resulting clean reads were aligned to the reference genome sequences (mm10_gencode) using Burrows-Wheeler Aligner (BWA) software, and genome browser views of specific gene loci were visualized using Integrative Genomics Viewer (IGV) software. For the cited datasets, GSE235281 was aligned to the mm9_gencode reference genome, while GSE234694 and GSE163463 were aligned to the mm10_gencode reference genome. Antibody information is provided in Supplementary Table S4.

### CUT&Tag sequencing and data analysis

CUT&Tag was performed according to a previously published protocol^42^. Briefly, cells were resuspended in 50 μl of antibody buffer containing the primary antibody (anti-Ep300.etc.) and incubated at 4 °C overnight. After this, the samples were exposed to a secondary antibody (anti-rabbit IgG antibody, goat monoclonal) at room temperature for 1 h. Then, the samples were coincubated with the pA-Tn5 adapter complex at room temperature for 1 h to disturb DNA fragmentation. Purified DNA obtained from the samples was subjected to library preparation and sequenced using the Illumina NovaSeq 6000 sequencer (Jiayin Biotechnology Ltd.). The Trimmomatic software was used to remove adapter sequences and low-quality reads. The resulting clean reads were aligned to the reference genome sequences (mm10_gencode) using the BWA software; genome browser views of specific gene loci were visualized using the IGV software. Antibody information is provided in Supplementary Table S4.

### SAM in vitro induction

Bone marrow cells were isolated from the tibia and femur of female C57BL/6 J mice and were plated at 1×10^6^ cells/well in flat bottom, tissue culture-treated 12-well plates in RPMI 1640 medium supplemented with 10% FBS. Bone marrow cells were stimulated with GM-CSF (20 ng/ml, PeproTech) alone for the first four days, followed by combined stimulation with GM-CSF (20 ng/ml) and TGF-β1 (20 ng/ml, PeproTech) for the subsequent three days. Both suspended and adherent cells were collected together, washed with PBS, and used for subsequent experimental evaluations. The administration time of CCS1477 is consistent with that of TGF-β1, with a concentration of 0.6 μM.

### Adoptive transfer of SAMs

SAMs were induced in vitro from bone marrow cells (CD45.2 background, donor cells) and adoptively transferred into ACLF recipient mice (CD45.1 background) via tail vein injection. Each mouse received 2 × 10^6^ cells per injection at three time points: 24 h before, immediately after (0 h), and 12 h after acute CCl_4_ challenge.

### Phagocytosis assay

The phagocytic capacity of SAMs in vitro was assessed with pH-rodoRed-labeled *E. coli* bacteria (Invitrogen) and an oxidative burst quantitative assay kit (Absin Bioscience). Briefly, the harvested induced SAMs were plated at 3 × 10^5^ cells/well in a 96-well plate. For LPS stimulation mimicking PAMPs, SAMs were stimulated with LPS alone or in combination with CCS1477 (2 μM) for 24 h. For PMA stimulation to induce oxidative burst, SAMs were stimulated with PMA alone or in combination with CCS1477 (2 μM) for 1.5 h; the treatment group required pre-stimulation with CCS1477 for 1.5 h prior to PMA stimulation. Then, cells were incubated for 1.5 h at 37 °C, previously opsonised with *E. coli* BioParticles opsonizing reagent (Invitrogen). This was followed by incubation (20 min, 37 °C) and staining for monocyte markers. The cells harvested subsequently were used for immunofluorescence imaging or flow cytometry experiments.

### Flow cytometry

Frozen PBMCs and LMCs were thawed, washed, and stained with fluorochrome-conjugated antibodies against specific surface markers for 30 min at 4 °C. Cells for the phagocytosis experiment were fixed with 1% paraformaldehyde before being mounted for analysis. All stained cells were analyzed with LSR Fortessa (BD Biosciences), LSR Fortessa X-20 analyzers (BD Biosciences), FACSymphony^TM^ A5 SE Cell Analyzer (BD Biosciences), or CytoFLEX LX (Beckman Coulter). Known positive and negative samples were used throughout. Representative flow cytometry gating strategies are provided in Supplementary Fig S15-S17. Antibody information is provided in Supplementary Table S4.

### Statistical analysis

Numerical data are presented as mean ± SD from at least three biological replicates obtained from independent experiments, unless otherwise indicated. Survival curves were compared using the two-sided log-rank (Mantel–Cox) test. Correlation analyses were performed using Pearson correlation or Spearman rank correlation. Group comparisons were performed using two-sided unpaired *t*-tests (Student’s or Welch’s, as appropriate), Mann-Whitney *U* tests, one-way ANOVA followed by Tukey’s multiple-comparisons test, Brown-Forsythe and Welch ANOVA followed by Games-Howell’s multiple-comparisons test, or Kruskal-Wallis tests followed by Dunn’s multiple-comparisons test, as appropriate. Differences between groups were analyzed in GraphPad Prism 8.0.1. Statistical significance was denoted by asterisks in the figures: *P < 0.05, **P < 0.01, ***P < 0.001, ****P < 0.0001.

### Reporting summary

Further information on research design is available in the Nature Portfolio Reporting Summary linked to this article.

## Supplementary information

Supplementary Information

Reporting Summary

Transparent Peer Review file

## Source data

Source Data

## Data Availability

The data supporting the findings from this study are available within the manuscript and its Supplementary Information. Source data are provided with this paper. The mouse single-cell RNA-sequencing data generated in this study are available under accession code GSE338157. The RAW264.7 EP300 CUT&Tag data generated in this study are available under accession code GSE338159. The RAW264.7 H3K27ac ChIP-seq data generated in this study are available under accession code GSE338158. The human HBV-related liver bulk RNA-seq dataset analyzed in this study has been previously deposited under accession code GSE298435 and was reanalyzed in the present study. The human scRNA-seq data were integrated from PRJNA913603 and GSE216314. The human HBV-related PBMC bulk RNA-seq data were obtained from SRP201023 and GSE168048. The human non-HBV-related liver bulk RNA-seq data were obtained from GSE139602. The human non-HBV-related PBMC bulk RNA-seq data were obtained from GSE180014. The mouse bone marrow-derived macrophage ATAC-seq and ChIP-seq datasets were obtained from GSE234694 and GSE235281, respectively. Source data are provided with this paper.

## Code availability

The software and algorithms used for data analysis in this study are well-established from previous work and are cited throughout the manuscript.
