# Supplementary material for: EP300 inhibition alleviates acute-on-chronic liver failure through modulation of scar-associated macrophages
Source: Nat Commun. 2026 Oct 7;17:11432. doi: 10.1038/s41467-026-78245-x (PMC13737046; doi:10.1038/s41467-026-78245-x)
Supplement: Supplementary file 1 [file 41467_2026_78245_MOESM1_ESM.pdf]

Supplementary Information

EP300 inhibition alleviates acute-on-chronic liver failure through modulation of scar-associated macrophages

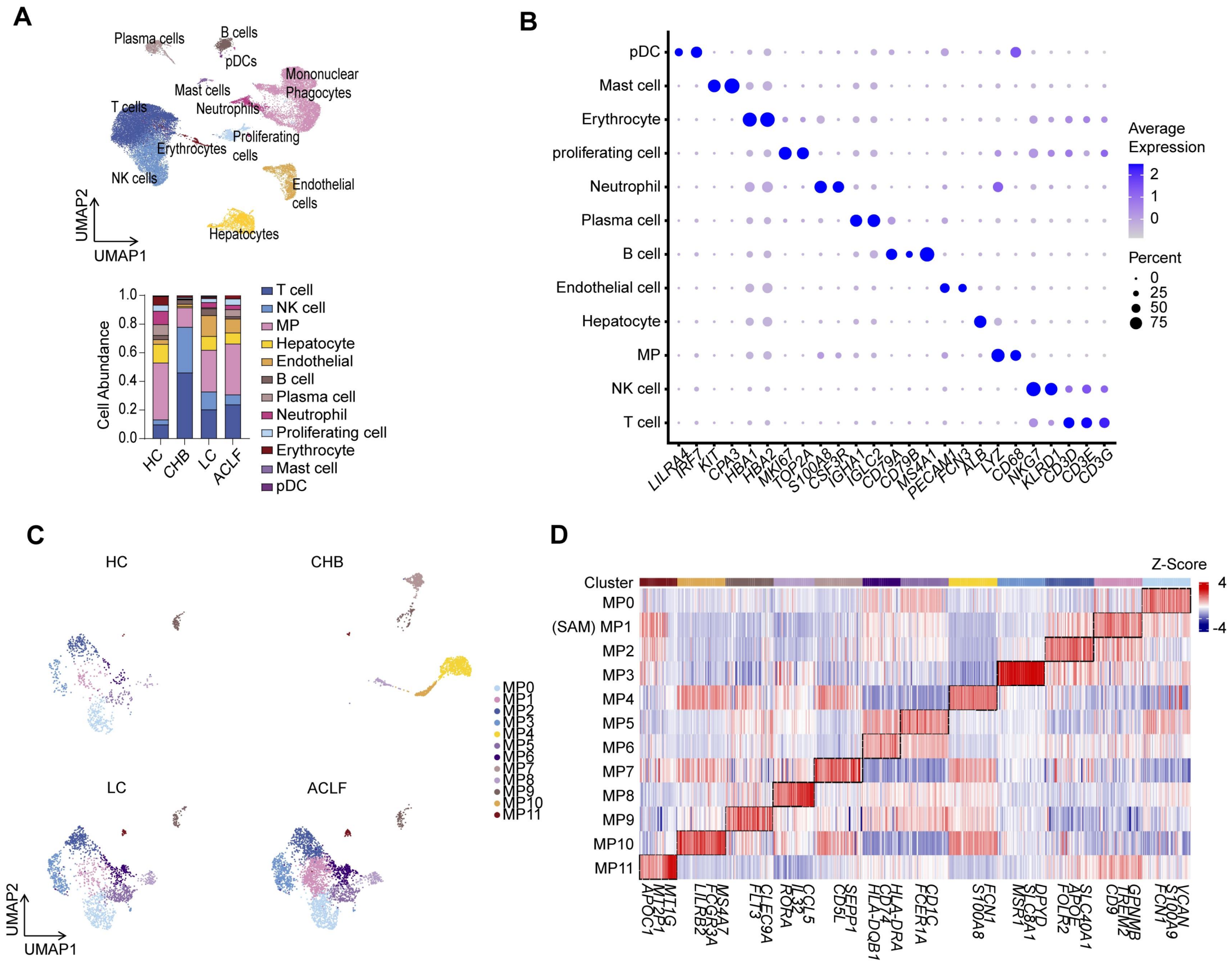

Figure S1. Single-cell transcriptomics of ACLF patients

(A) UMAP embedding of all cell types from integrated liver datasets (upper panel) and their proportions at HC and different stages of HBV-related liver disease (lower panel). (B) Dot plot showing representative marker genes for the identification of each cell types. (C) Differences in the frequency of MP subpopulations between HC and each stage of HBV-related liver disease, shown on UMAP embedding. (D) Heatmap of the top 50 genes for MP subpopulations, with marker genes specifically highlighted. Source data are provided as a Source Data file.

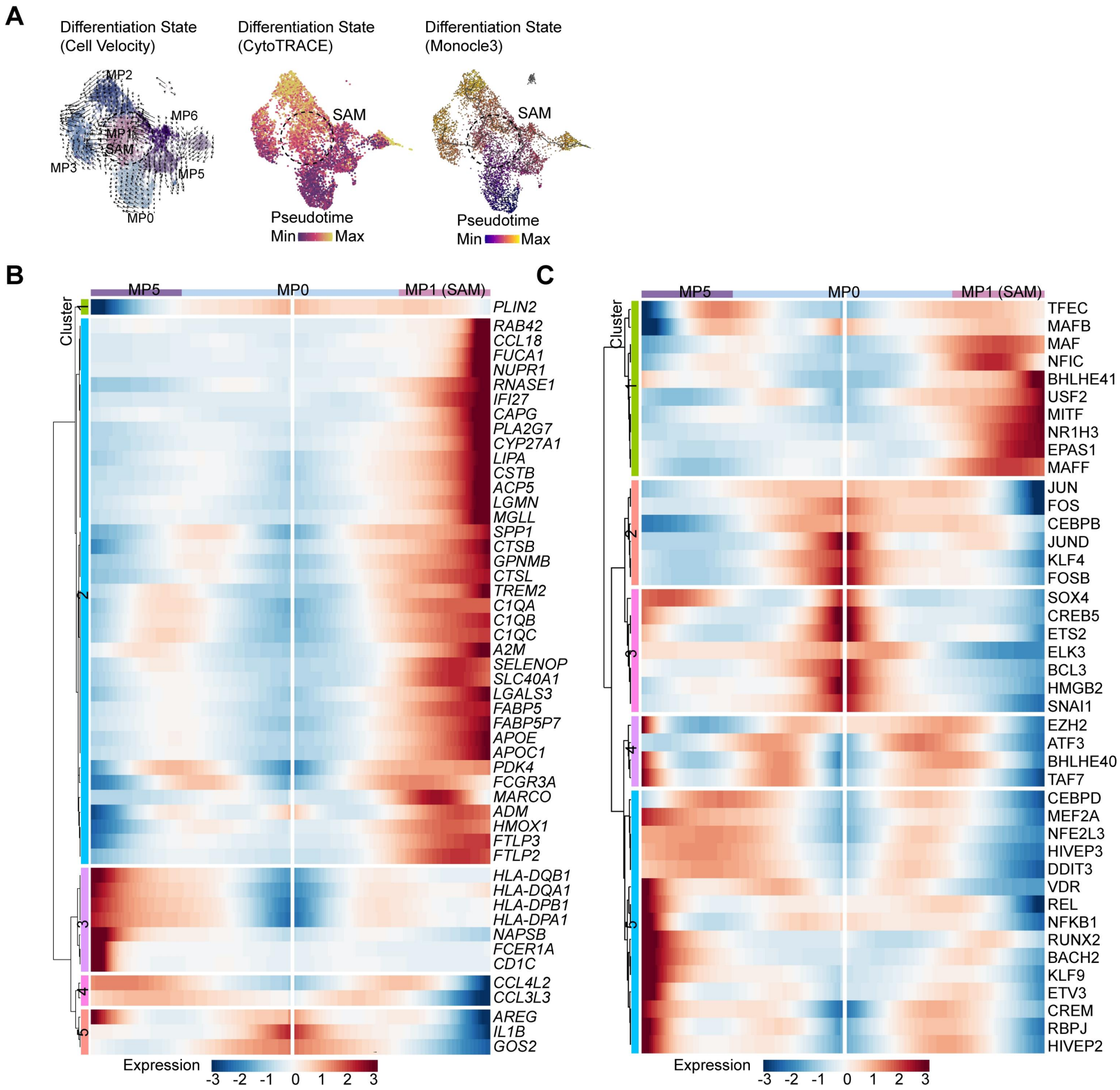

Figure S2. Developmental trajectory of SAMs

(A) Differentiation state estimated by Cell Velocity, CytoTRACE, Monocle3. (B) Heatmap showing genes that undergo changes during SAM differentiation (with the trajectory from MP0 to MP5 used as an exclusion control), clustered by alteration pattern. (C) Heatmap showing TFs that undergo changes during SAM differentiation (with the trajectory from MP0 to MP5 used as an exclusion control), clustered by alteration pattern.

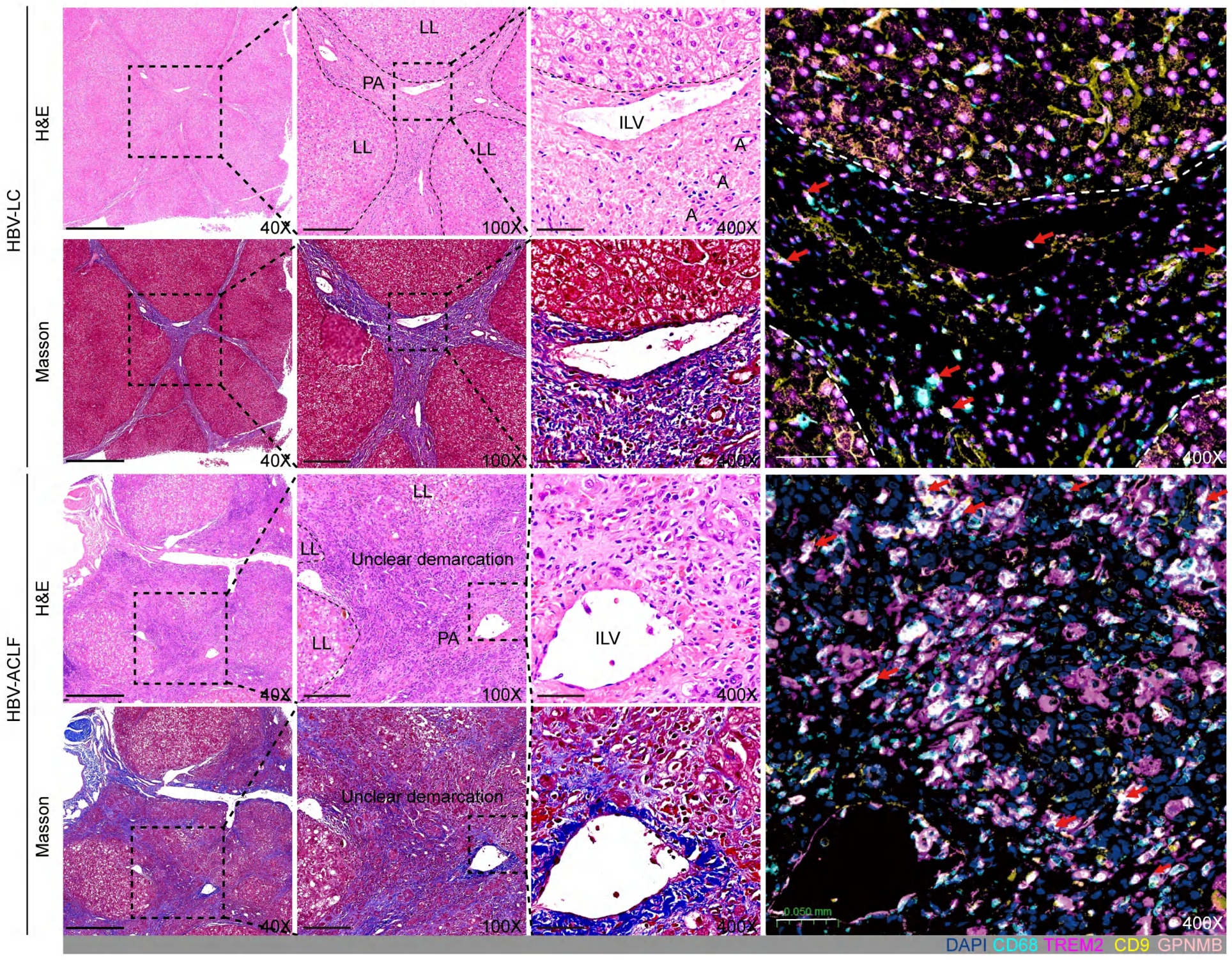

Figure S3. Pathological characteristics and SAMs infiltration sites in HBV-related LC and HBV-related ACLF patients.

Representative H&E staining, Masson trichrome, and mIHC images of the patient's liver (n = 11 for LC and n = 13 for ACLF, biologically independent human samples). Compared with LC, ACLF livers show disruption of fibrous septa, poorly demarcated portal and lobular structures, and broader SAM infiltration around the portal areas. Scale bars: 0.625 mm at 40x, 0.2 mm at 100x, and 0.05 mm at 400x. SAMs (red arrows) were identified using CD68, TREM2, CD9, and GPNMB. PA, portal area; LL, liver lobe; ILV, interlobular vein; A, artery.

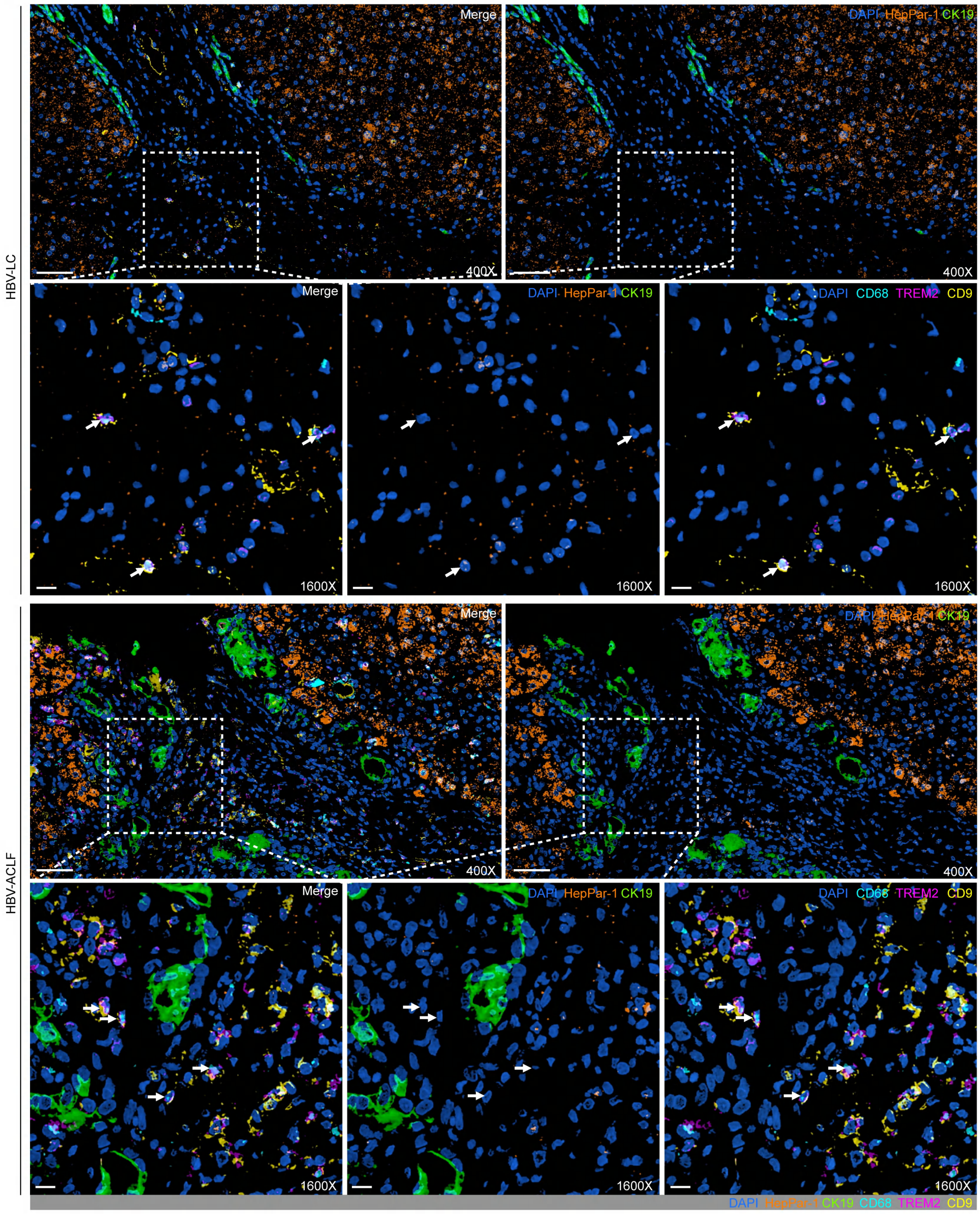

Figure S4. SAMs infiltration sites in HBV-related LC and HBV-related ACLF patients.

Hepatocyte marker (HepPar-1) and cholangiocyte marker (CK19) were used to identify liver structures, while CD68, TREM2, and CD9 were used to identify SAMs (White arrows) (n = 5 biologically independent human samples per group). Scale bars, 0.05 mm at 400x and 0.025 mm at 1600x. LC, liver cirrhosis; ACLF, Acute-on-Chronic Liver Failure.

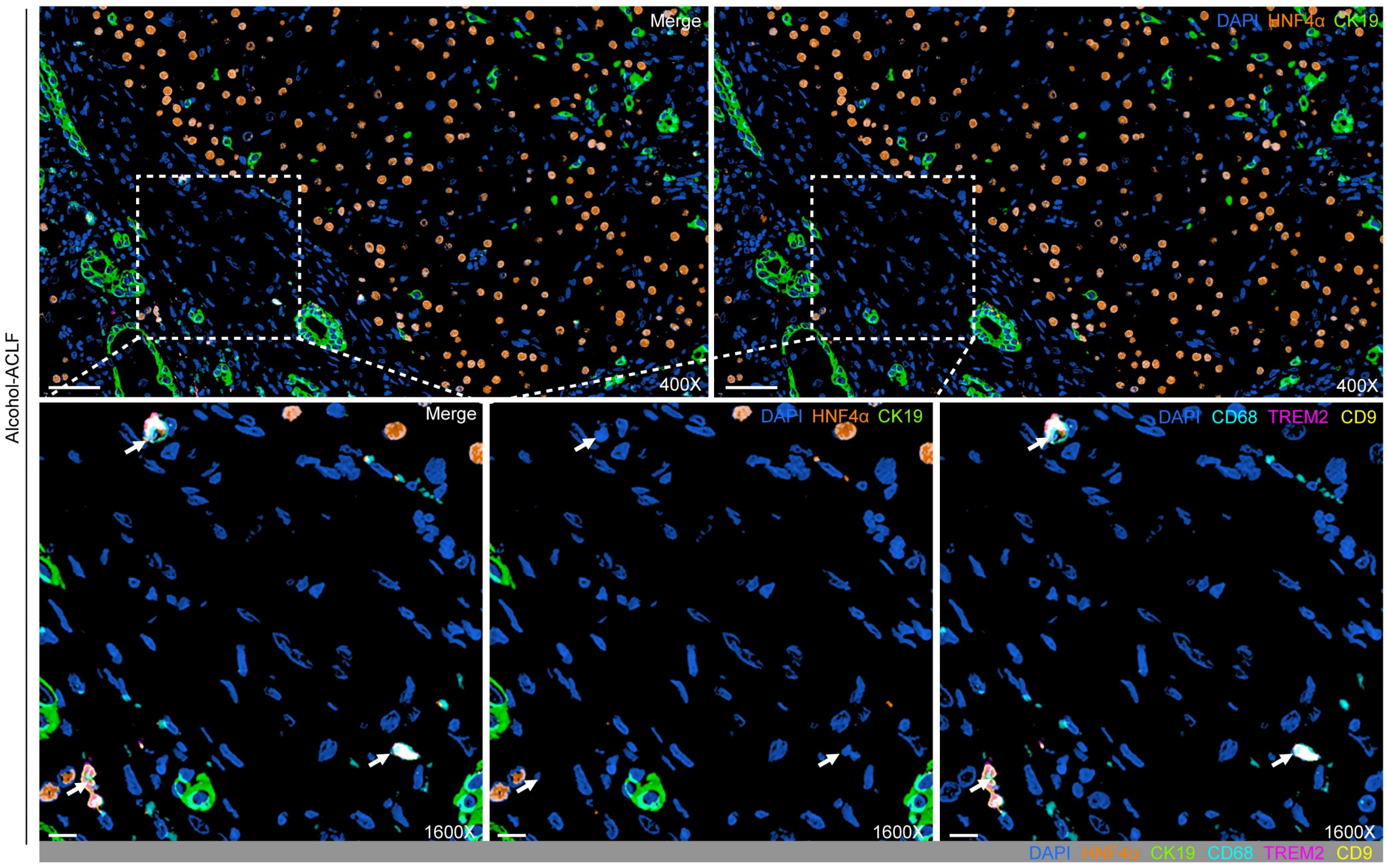

Figure S5. SAMs infiltration sites in alcohol-related ACLF patients.
Representative mIHC images of the ACLF patients (n = 3 biologically independent human samples). Scale bars, 0.05 mm at 400x and 0.025 mm at 1600x. Hepatocyte marker (HNF4α) and cholangiocyte marker (CK19) were used to identify liver structures; CD68, TREM2, and CD9 were used to identify SAMs (White arrows).

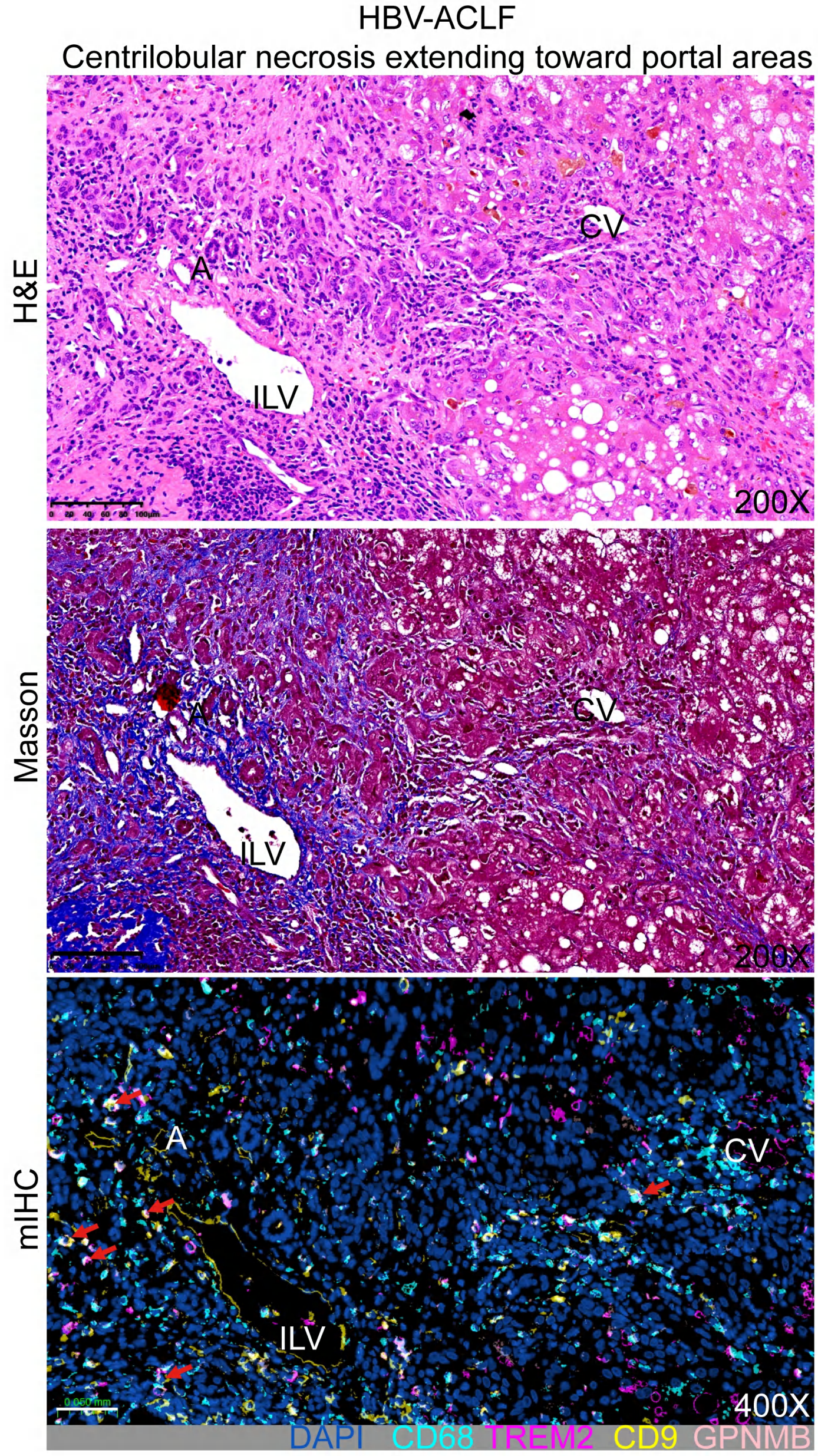

Figure S6. Pathological characteristics and SAMs infiltration sites in HBV-related ACLF patients.

Representative H&E staining, Masson trichrome, and mIHC images of the patient's liver (n = 13 for ACLF, biologically independent human samples). In the liver of HBV-ACLF patient, centrilobular necrosis originates from the central vein (CV) and extends toward the portal area, showing blurred boundaries between necrotic and periportal regions. SAMs (red arrows) were enriched in and around the PA, identified using CD68, TREM2, CD9, and GPNMB. Scale bars: 0.1 mm at 200x and 0.05 mm at 400x. A, artery; ILV, interlobular vein.

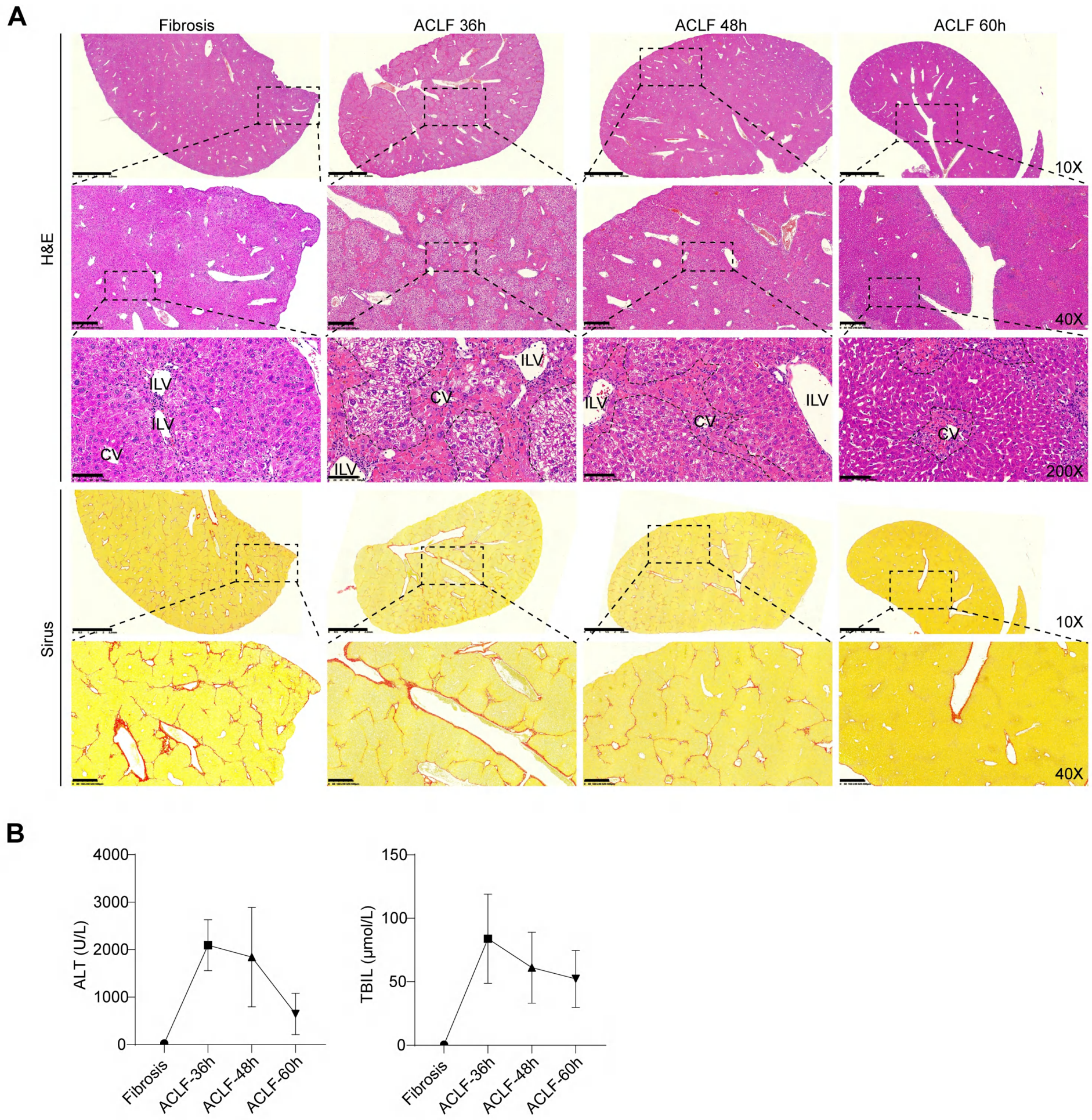

Figure S7. The pathological characteristics of fibrosis and ACLF mice

(A) Representative H&E and Sirus Red staining images of the mouse livers (n = 5 biologically independent mice samples per group). Scale bars, 2.5 mm at 10x, 0.4 mm at 40x,and 0.1 mm at 200x. (B) Serum ALT and TBIL levels in ACLF model mice at different time points (n = 8 biologically independent mice samples per group). ILV, interlobular vein; CV, central vein.

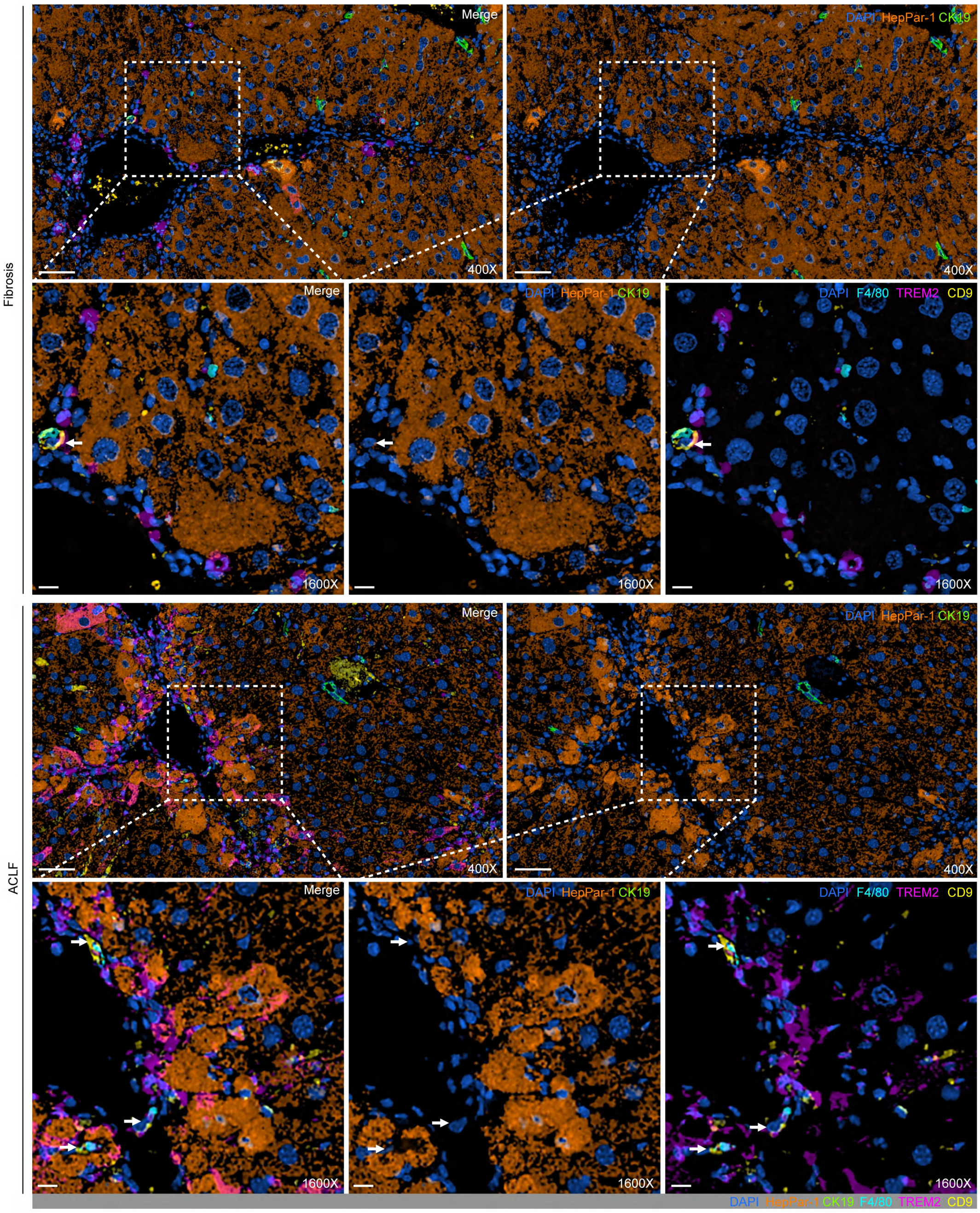

Figure S8. SAMs infiltration sites in fibrosis and ACLF mice.

Representative mIHC images of the mouse livers (n = 5 biologically independent mice samples per group). Hepatocyte marker (HepPar-1) and cholangiocyte marker (CK19) were used to identify liver structures, while F4/80, TREM2, and CD9 were used to identify SAMs (White arrows). Scale bars, 0.05 mm at 400x and 0.025 mm at 1600x.

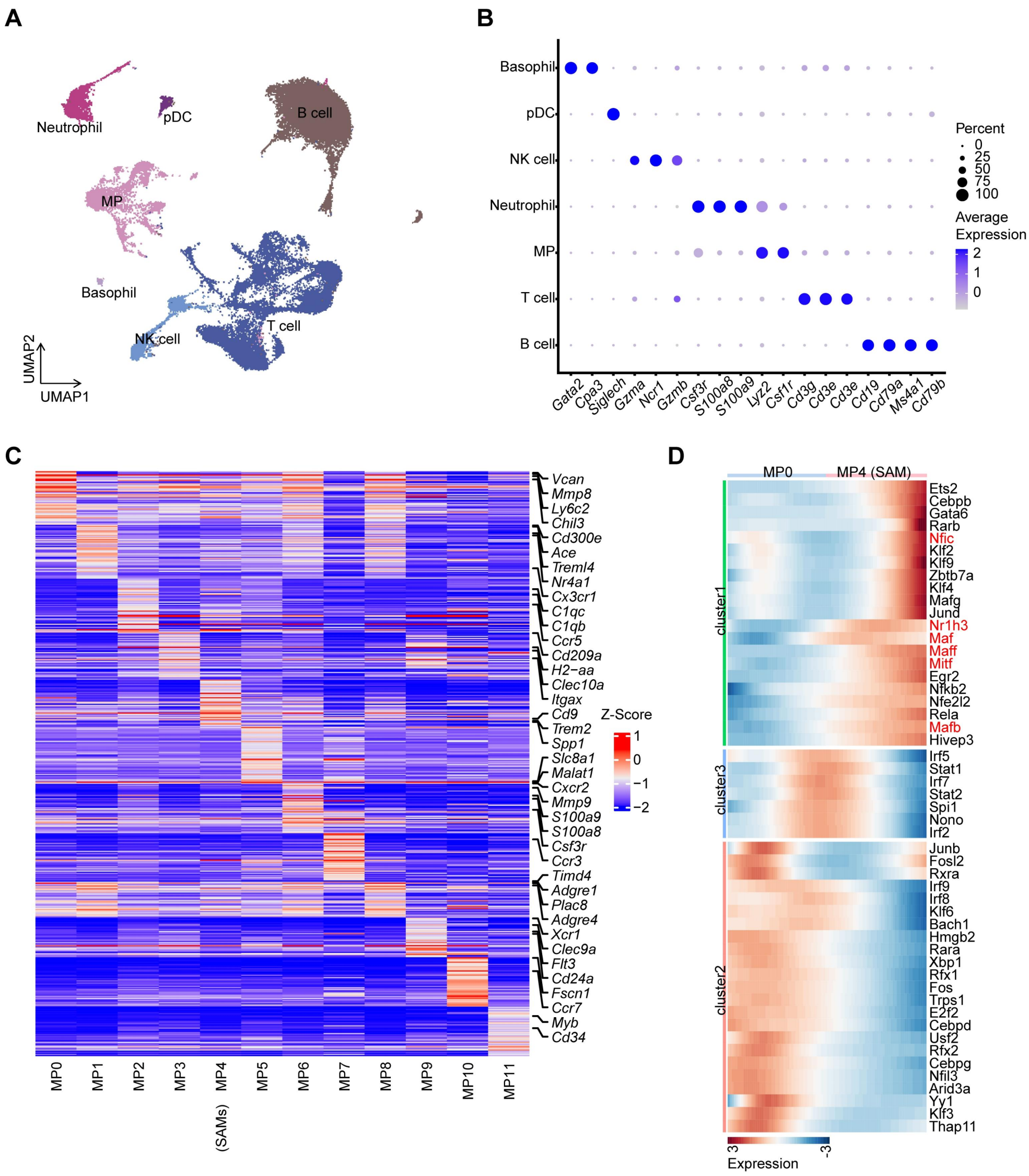

Figure S9. Single-cell transcriptomics of ACLF mice

(A) UMAP embedding of $Cd45^{+}$ immune cells sorted from the liver of fibrosis and ACLF model mice. (B) Dot plot showing representative marker genes for identification of immune cell populations. (C) Heatmap of the top 50 genes for MP subpopulations, with marker genes specifically highlight. (D) Heatmap showing TFs that undergo changes during SAM differentiation in mice scRNA-seq data, clustered by alteration pattern.

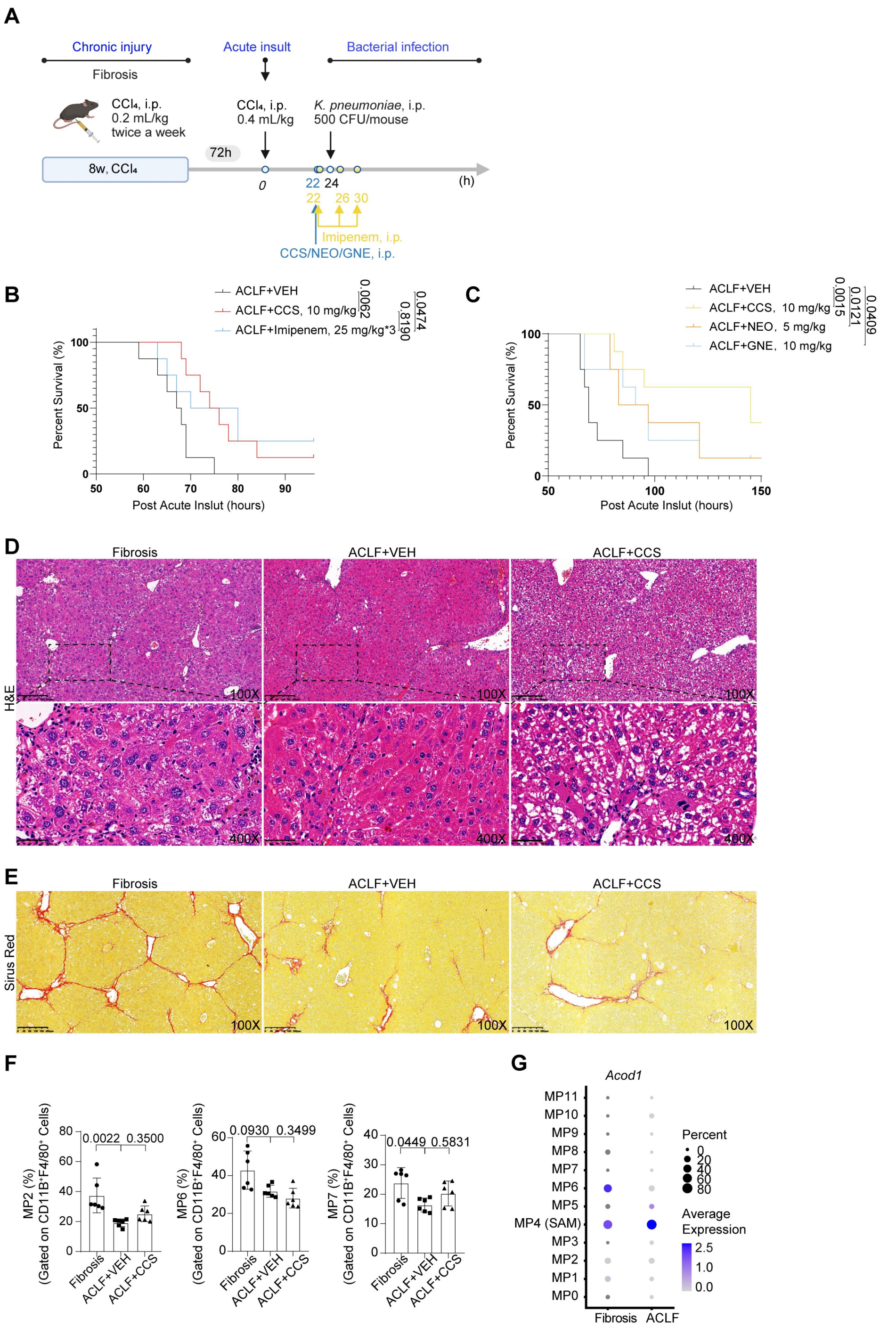

Figure S10. Ep300 inhibitor treatment in ACLF mice

(A) Schematic illustration of compound administration. Created in BioRender. Yu, P. (2026) https://BioRender.com/cs0j9mo. (B) Survival analysis of ACLF model mice treated with vehicle (black line), CCS1477 (10 mg/kg, red line) or imitate (25 mg/kg*3, blue line) (n = 8). (C) Survival analysis of ACLF model mice treated with vehicle (black line), CCS1477 (10 mg/kg, yellow line), NEO2734 (5 mg/kg, orange line) and GNE207 (10 mg/kg, blue line) (n = 8). (D) Representative H&E staining image of the mice livers (n = 5 biologically independent mice per group). Scale bars, 0.2 mm at 100x, and 0.05 mm at 400x. (E) Representative Sirus Red staining image of the mice livers (n = 5 biologically independent mice per group). Scale bars, 0.2 mm at 100x. (F) Flow

cytometry analysis of the changes in the proportion of MP2 (CCR5$^+$CXCR3$^+$), MP6 (CXCR2$^+$CD9$^+$) and MP7 (CCR3$^+$) within the CD11B$^+$F4/80$^+$ gated population in liver of fibrosis and ACLF model mice with vehicle or CCS1477 (n = 6) (G) Dot plot showing *Acod1* expression across MPs subsets in the liver of fibrosis and ACLF model mice. Sample sizes (n) represent biologically independent mice, as indicated in each panel. Bars represent mean ± SD. In (B, C), survival curves were compared using the two-sided log-rank (Mantel-Cox) test. In (F, MP2 and MP7), Kruskal-Wallis test followed by Dunn's multiple-comparisons test was used. In (F, MP6), Brown-Forsythe and Welch ANOVA followed by Games-Howell's multiple-comparisons test was used. VEH, vehicle; CCS, CCS1477; NEO, NEO2734; GNE, GNE207. Source data are provided as a Source Data file.

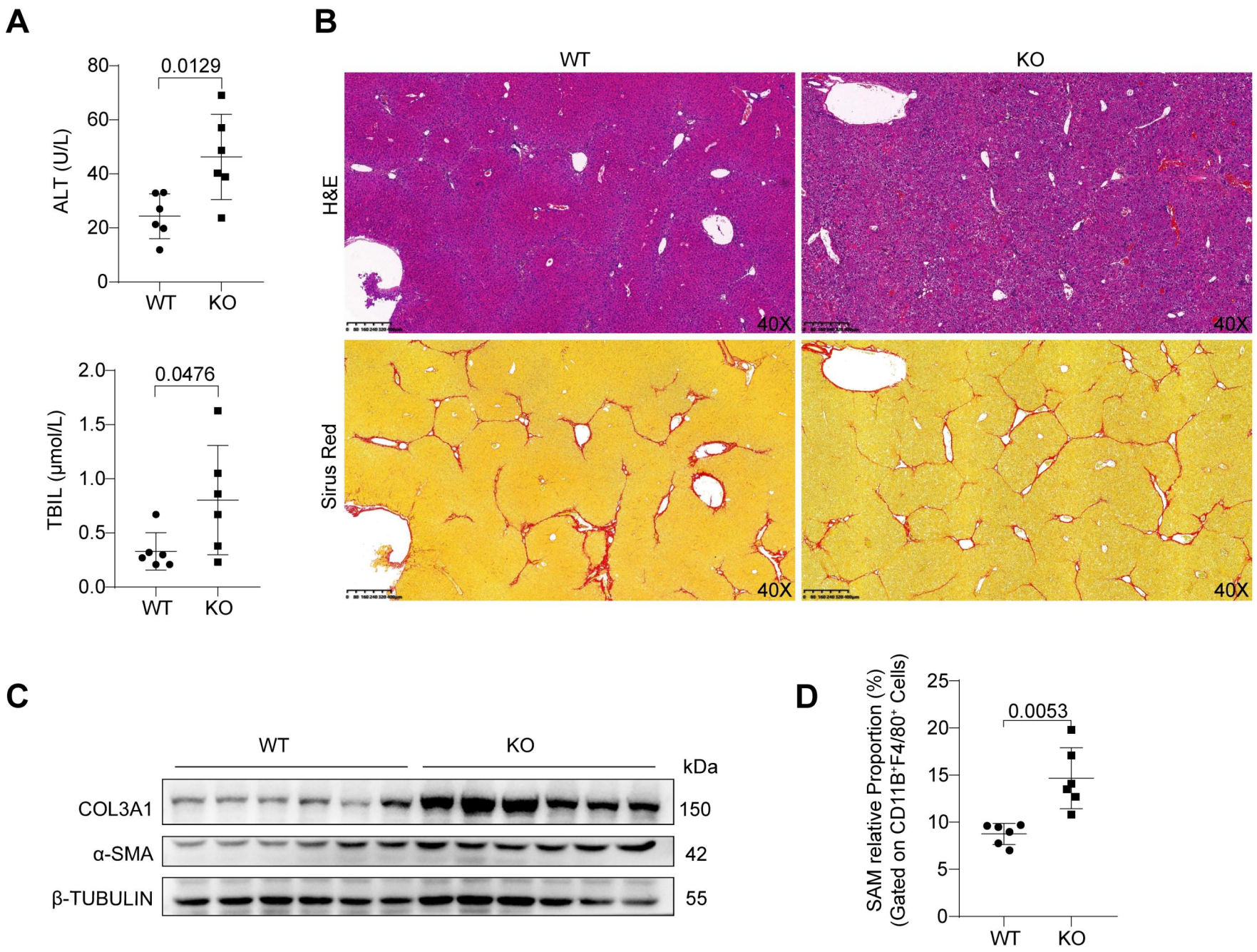

Figure S11. *Acod1$^{Mye-/-}$* mice exhibit exacerbated liver fibrosis and increased numbers of SAMs.

(A) Serum ALT and TBIL levels in the fibrosis mouse model of WT and *Acod1$^{Mye-/-}$* mice (n = 6 biologically independent mice per group). (B) Representative images of H&E and Sirius Red staining in the liver (n = 5 biologically independent mice samples per group). (C) Western blot analysis showing protein levels of COL3A1 and α-SMA in the liver (n = 3 biologically independent experiments). (D) Flow cytometry analysis in the proportion of SAMs (TREM2$^+$CD9$^+$) within the CD11B$^+$F4/80$^+$ gated population in liver (n = 6 biologically independent mice samples per group). Bars represent mean ± SD. In (A), a two-sided unpaired Student's t-test was used for ALT and a two-sided Mann-Whitney U test was used for TBIL. In (D), a two-sided unpaired Welch's t-test was used. Source data are provided as a Source Data file.

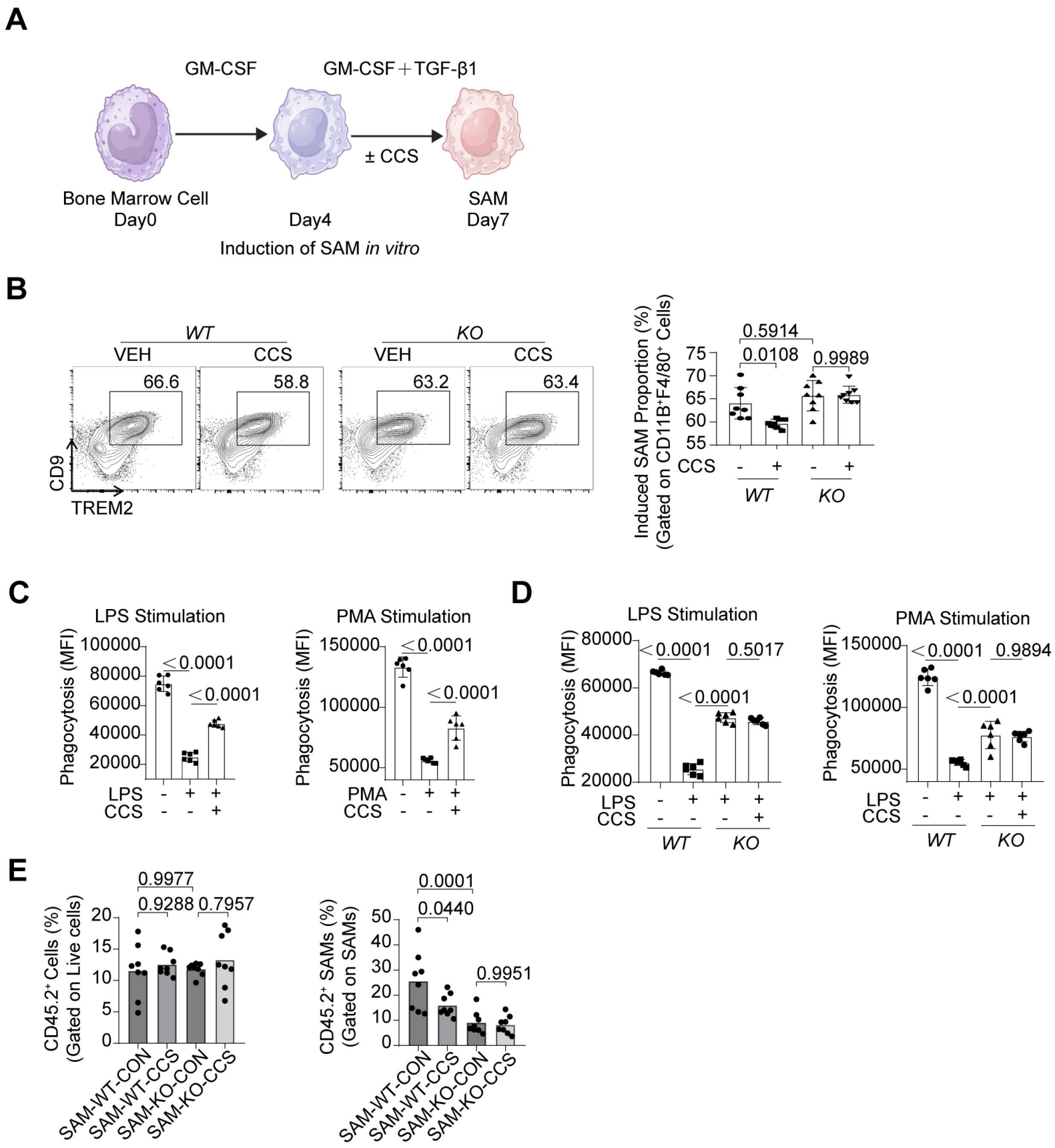

Figure S12. Induction of SAMs *in vitro* and assessment of their bacterial phagocytic capacity under ACLF-like conditions.

(A) Schematic diagram of the *in vitro* assay for the induction of SAMs and CCS treatment. Created in BioRender. Yu, P. (2026) https://BioRender.com/c68u638. (B) Representative flow cytometry gating schematic (left panel) and statistical analyses (right panel) showing the proportion of induced SAMs ($TREM2^+CD9^+$) within the $CD11B^+F4/80^+$ gated population, as determined by the assay described in (A) (n = 8 biologically independent mice samples per group). (C) Phagocytosis of *E. coli* by induced SAMs from WT mice under indicated conditions (± LPS/PMA, ± CCS1477), accessed by flow cytometry (n = 6 biologically independent mice samples per group). (D)Phagocytosis of *E. coli* by induced SAMs from WT mice and *Acod1*$^{Mye-/-}$ mice under indicated conditions (± LPS/PMA, ± CCS1477), accessed by flow cytometry (n = 6 biologically independent mice samples per group) (E) Flow cytometry analysis showing the proportion of $CD45.2^+$ cells within the live cell population and SAMs ($TREM2^+CD9^+$) within the $CD11B^+F4/80^+$ gated population in CD45.1 ACLF recipient mice after adoptive transfer of SAMs from different groups (n = 8 biologically independent mice samples per group). Bars represent mean ± SD. In (B-D) and (E, $CD45.2^+$SAMs), one-way ANOVA followed by Tukey's multiple-comparisons test was used. In (E, $CD45.2^+$ cells), Brown-Forsythe and Welch ANOVA followed by Games-Howell's multiple-comparisons test was used. VEH, vehicle; CCS, CCS1477; KO, *Acod1*$^{Mye-/-}$; MFI, Mean Fluorescence Intensity; SAM-WT-CON, *in vitro*-induced SAMs from WT mouse bone marrow treated with DMSO; SAM-WT-CCS, *in*

vitro-induced SAMs from WT mouse bone marrow treated with CCS1477; SAM-KO-CON, *in vitro*-induced SAMs from *Acod1*$^{Mye-/-}$ mouse bone marrow treated with DMSO; SAM-KO-CCS, *in vitro*-induced SAMs from *Acod1*$^{Mye-/-}$ mouse bone marrow treated with CCS1477. Source data are provided as a Source Data file.

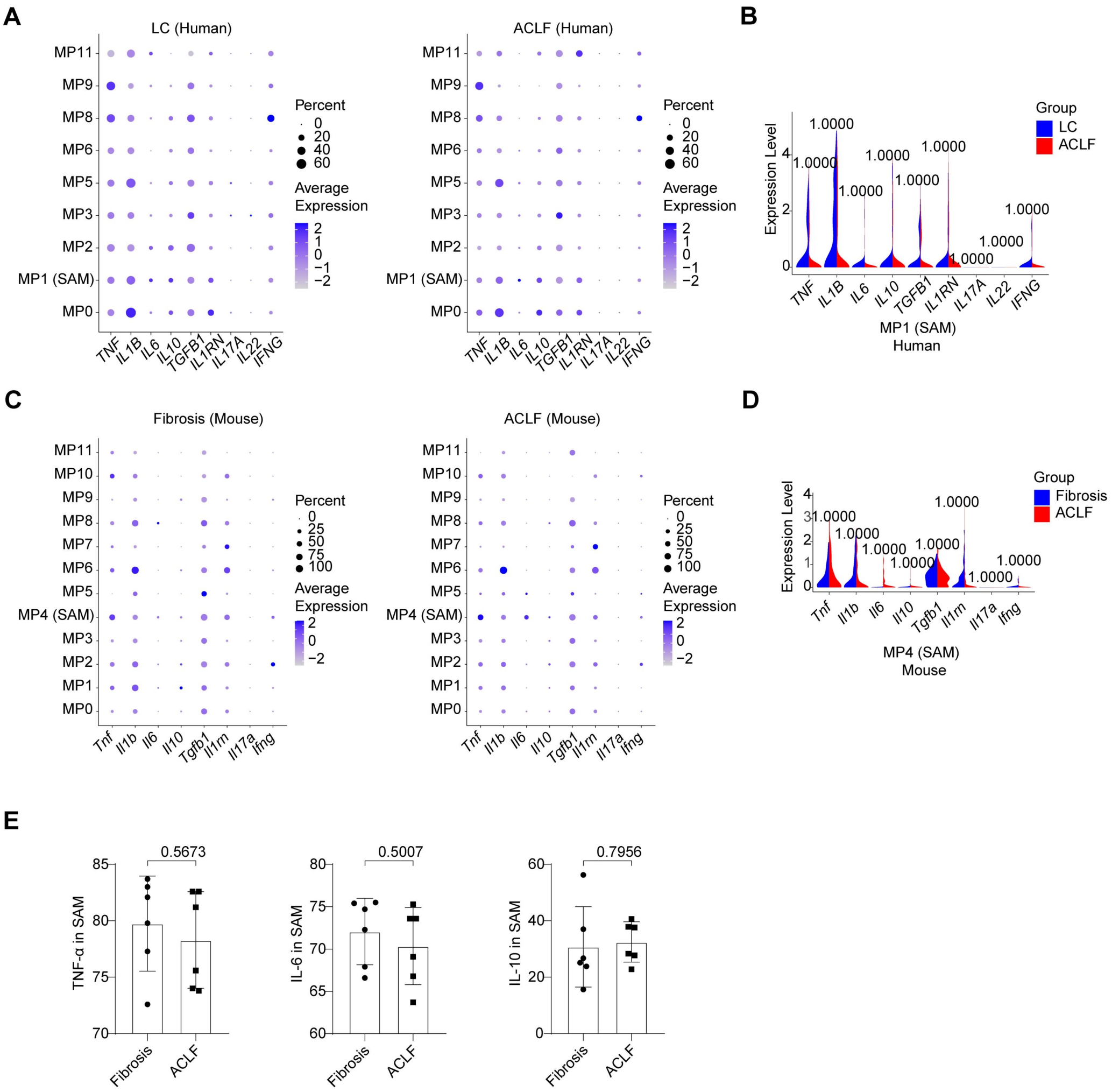

Figure S13. Cytokine secretion by MPs.

(A) Dot plot showing cytokine expression across MP subsets in the livers of LC and ACLF patients. (B) Violin plot showing cytokine expression in MP1 (SAMs) between LC and ACLF patients. (C) Dot plot showing cytokine expression across MP subsets in the livers of fibrosis and ACLF mouse models. (D) Violin plot showing cytokine expression in MP4 (SAMs) between fibrosis and ACLF mouse models. (E) Flow cytometry analysis showing cytokine secretion in SAMs (TREM2$^+$CD9$^+$) from fibrosis and ACLF mice (n = 6 biologically independent mice samples per group). Bars represent mean ± SD. In (B, D), differential expression was analyzed using the Wilcoxon rank-sum test. In (E), a two-sided unpaired Student's t-test was used. Source data are provided as a Source Data file.

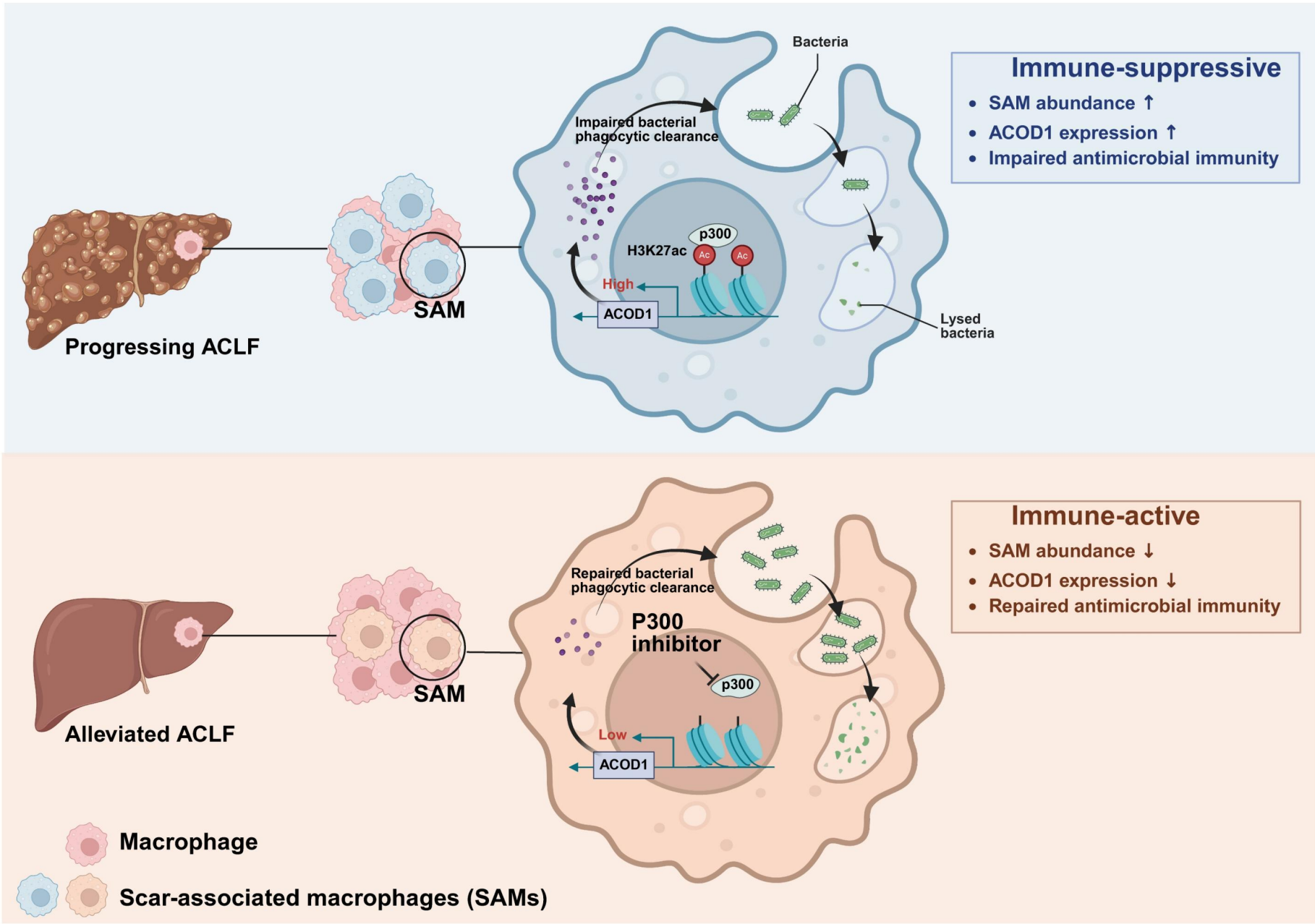

Figure S14. Schematic summary of EP300 inhibition alleviating ACLF by modulating SAMs via ACOD1.

Created in BioRender. Yu, P. (2026) https://BioRender.com/d94l124.

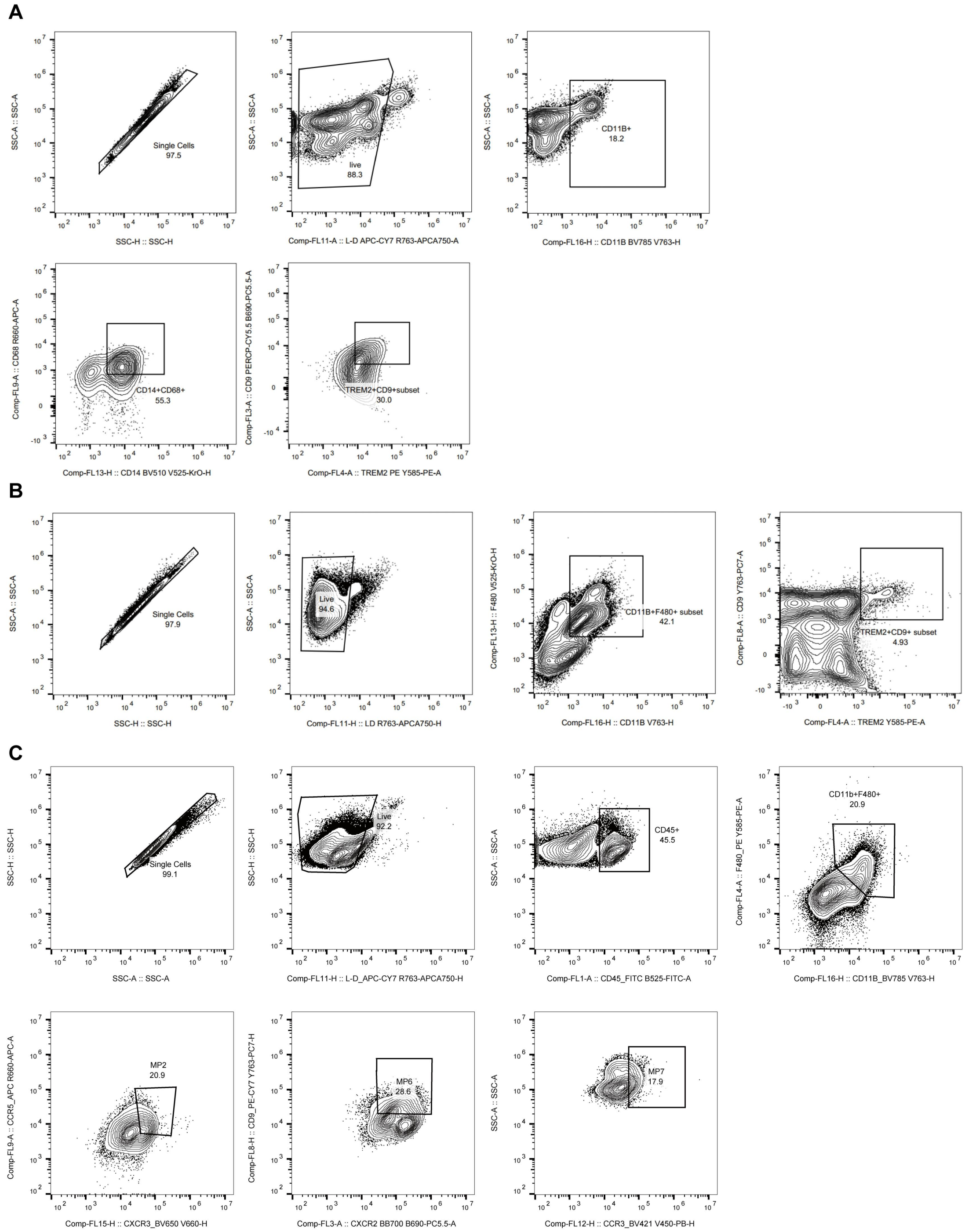

Figure S15. Flow cytometry gating strategy.

(A) Representative flow cytometry gating strategy for the analysis shown in Fig. 1I. (B) Representative flow cytometry gating strategy for the analyses shown in Fig. 2B, 3G and 4C. (C) Representative flow cytometry gating strategy for the analysis shown in Supplementary Fig. S10F.

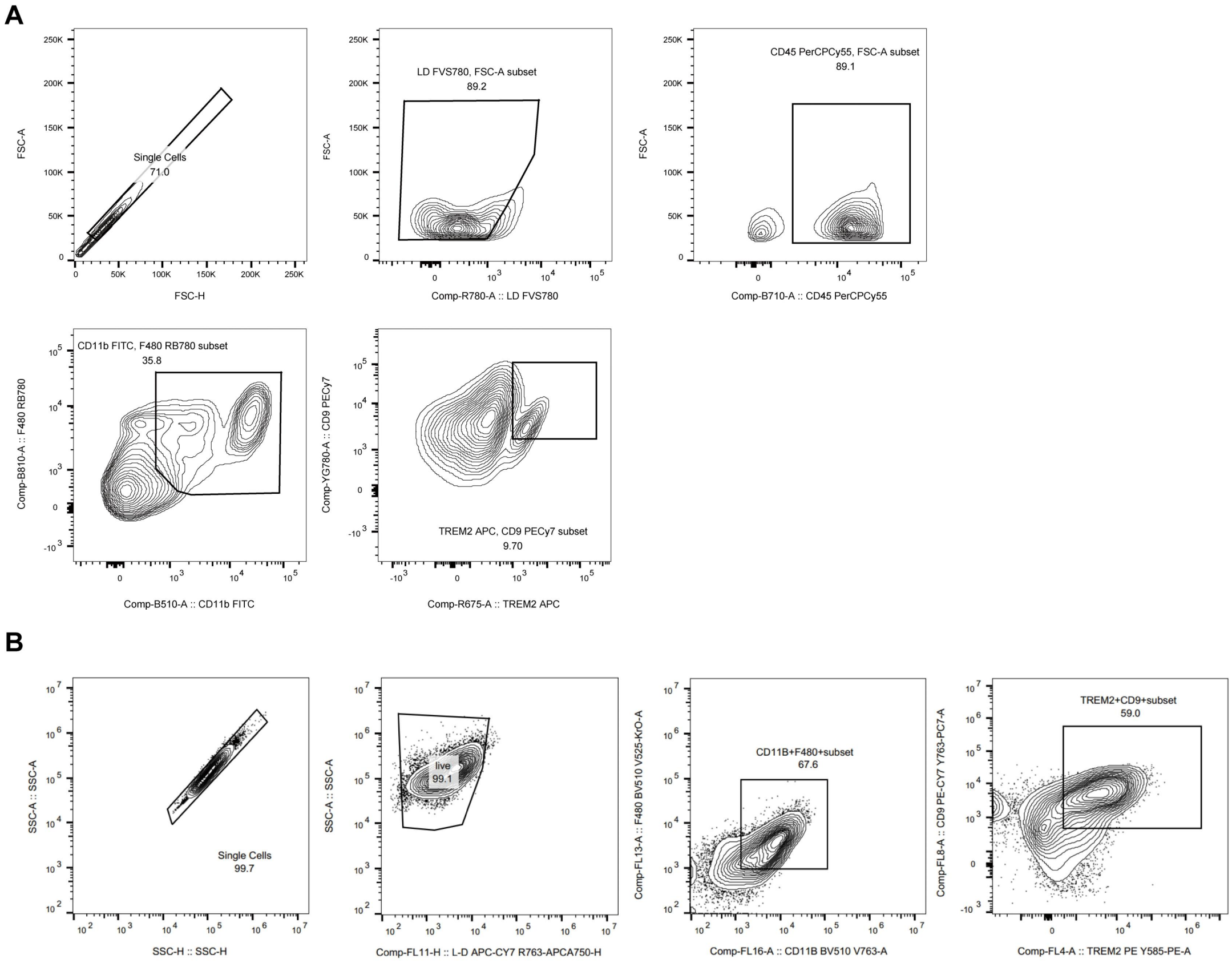

Figure S16. Flow cytometry gating strategy.

(A) Representative flow cytometry gating strategy for the analyses shown in Fig. 1D and 13E. (B) Representative flow cytometry gating strategy for the analysis shown in Supplementary Fig. S12E.

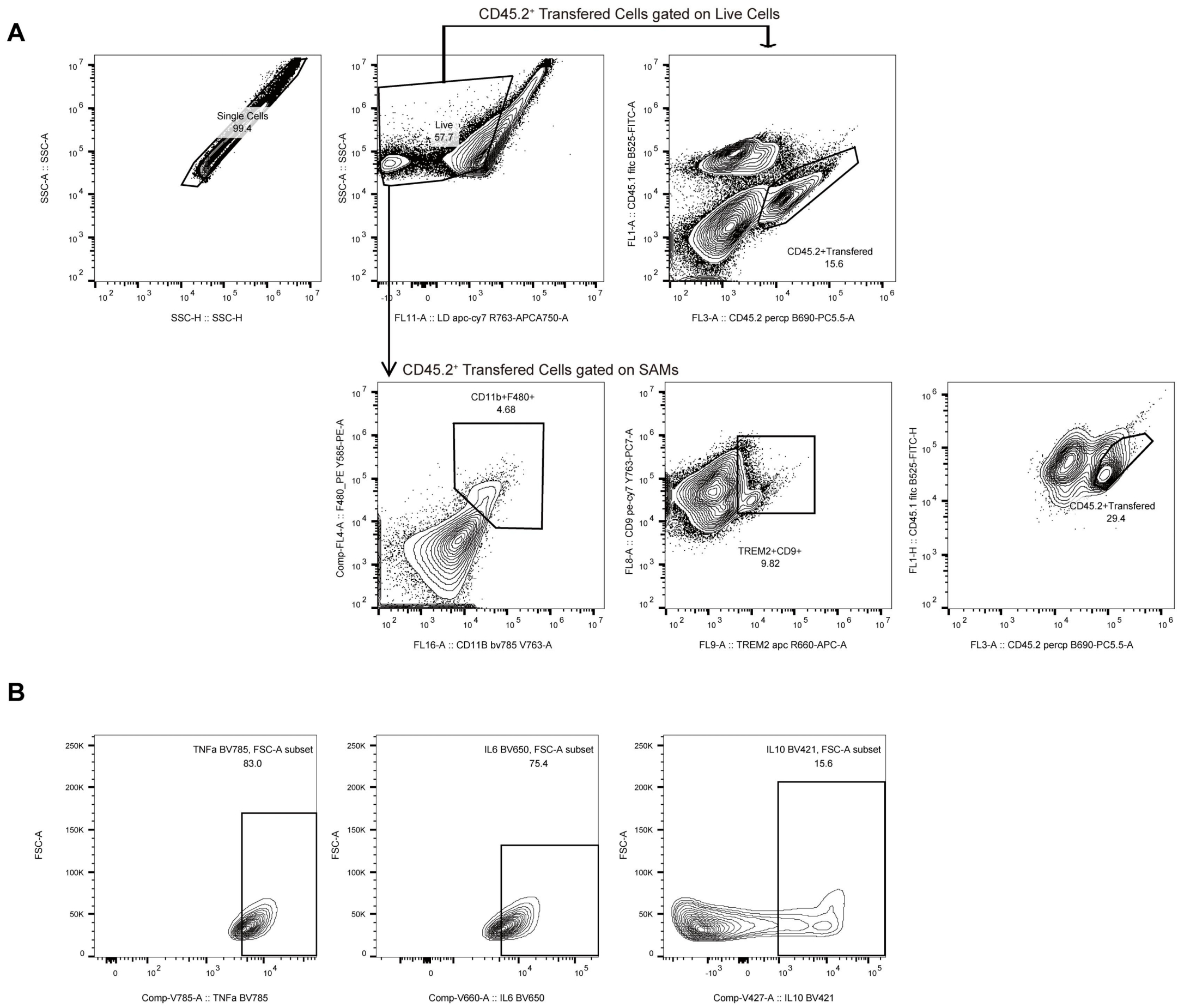

Figure S17. Flow cytometry gating strategy.

(A) Representative flow cytometry gating strategy for the analysis shown in Supplementary Fig. S12E. (B) Representative flow cytometry gating strategy for the analysis shown in Supplementary Fig. S13E.

Table S1 Clinical characteristics of patients in Flow cytometry cohort of PBMCs		
Characteristics	ACLF (n = 27)	LC (n = 34)
Age (years)	48.0 (38.0, 50.8)	59 (48, 65.3)
Male (No.)	85.19% (23)	76.47% (26)
HBV DNA level (IU/mL)		
$\leq 2\times10^2$	48.15% (13)	91.18% (31)
2×10^2-2×10^6	51.85% (14)	5.88% (2)
$>2\times10^6$	0.00% (0)	2.94% (1)
Laboratory data		
Alanine aminotransferase (U/L)	92.0 (38.0, 152.0)<br>**, P=0.0039	29.0 (22.5, 62.5)
Aspartate aminotransferase (U/L)	73.0 (54.0, 144.0)<br>**, P=0.0024	40.0 (33.0, 67.3)
Albumin (g/L)	35.6 (32.3, 39.2)<br>*, P=0.0146	30.2 (25.8, 35.0)
Total bilirubin (μmol/L)	388.6 (223.7, 492.9)<br>****, P＜0.0001	38.0 (19.7, 61.6)
Creatinine (μmol/L)	54.0 (45.0, 74.0)<br>n.s., P=0.3422	62.9 (49.8, 79.6)
Sodium (mmol/L)	135.0 (133.0, 137.0)<br>***, P=0.0003	139.4 (136.0, 142.3)
White blood cell count (10^9/L)	5.5 (3.6, 8.7)<br>**, P=0.0077	3.4 (2.4, 5.7)
Platelet count (10^9/L)	48.0 (38.0, 99.0)<br>n.s., P=0.9512	52.0 (36.5, 111.8)
Haemoglobin (g/L)	101.0 (79.0, 132.0)<br>n.s., P=0.9339	107.0 (82.8, 122.3)
INR	2.2 (1.8, 2.9)<br>****, P＜0.0001	1.4 (1.2, 1.5)
Severity score		
MELD	28.0 (23.0, 31.0)<br>****, P＜0.0001	13.0 (10.8, 15.3)
MELD-Na	31.0 (25.0, 34.0)<br>****, P＜0.0001	13.0 (10.8, 15.3)

Data are presented as medians (P25, P75) or percentages (number of patients). Statistical significance was calculated using two-sided Mann-Whitney U tests for alanine aminotransferase, aspartate aminotransferase, creatinine, sodium, white blood cell count, platelet count, haemoglobin and INR; two-sided unpaired Student's t-tests for albumin and MELD; and two-sided unpaired Welch's t-tests for total bilirubin and MELD-Na. Statistical significance is indicated as follows: ****P < 0.0001, ***P < 0.001, **P < 0.01, *P < 0.05, *n.s.*, not significant. INR, international normalized ratio; MELD-Na, Model for End-Stage Liver Disease Sodium.

Table S2 Clinical characteristics of patients in mIHC of livers

Characteristics	ACLF (n = 13)	LC (n = 11)
Age (years)	40.0 (38.0, 49.0)	47.0 (40.0, 57.0)
Male (No.)	92.3% (12)	81.8% (9)
HBV DNA level (IU/mL)		
$\le 2\times10^2$	23.1% (3)	18.2% (2)
2×10^2 –2×10^6	69.2% (9)	81.8% (9)
$>2\times10^6$	7.7% (1)	0.0% (0)
Laboratory data		
Alanine aminotransferase (U/L)	131.0 (117.5, 361.5)<br>****, P＜0.0001	24.0 (17.0, 45.0)
Aspartate aminotransferase (U/L)	233.0 (148.0, 367.5)<br>****, P＜0.0001	33.0 (23.0, 61.0)
Albumin (g/L)	33.8 (30.9, 37.8)<br>n.s., P=0.4063	36.1 (31.5, 39.6)
Total bilirubin (μmol/L)	531.9 (512.6, 626.4)<br>****, P＜0.0001	20.8 (19.4, 28.9)
Creatinine (μmol/L)	63.0 (52.0, 78.5)<br>n.s., P=0.6636	67.0 (52.0, 75.0)
Sodium (mmol/L)	137.0 (130.5, 140.0)<br>**, P=0.0017	143.0 (142.0, 146.0)
White blood cell count (10^9/L)	6.3 (4.6, 10.1)<br>***, P=0.0009	3.6 (2.6, 3.9)
Platelet count (10^9/L)	62 (36.5, 105.5)<br>n.s., P=0.5149	75.0 (49.5, 107.8)
Haemoglobin (g/L)	111.0 (102.0, 126.0)<br>n.s., P=0.2387	116.0 (69.0, 122.0)
INR	2.4 (2.1, 3.1)<br>****, P＜0.0001	1.3 (1.2, 1.4)
Severity score		
MELD	30.0 (26.5, 32.0)<br>****, P＜0.0001	11.0 (9.0, 13.0)
MELD-Na	31.0 (26.5, 38.5)<br>****, P＜0.0001	11.0 (9.0, 13.0)

Data are presented as medians (P25, P75) or percentages (number of patients). Statistical significance was calculated using two-sided Mann-Whitney U tests for alanine aminotransferase, albumin, total bilirubin, creatinine, white blood cell count, platelet count and MELD; two-sided unpaired Student's t-tests for sodium and haemoglobin; and two-sided unpaired Welch's t-tests for aspartate aminotransferase, INR and MELD-Na.Statistical significance is indicated as follows: ****$P < 0.0001$, ***$P < 0.001$, **$P < 0.01$, *n.s.*, not significant. INR, international normalized ratio; MELD-Na, Model for End-Stage Liver Disease Sodium.

Table S3 Clinical characteristics of patients in mRNA of LMC		
Characteristics	ACLF (n = 10)	LC (n = 10)
Age (years)	49.0 (38.8, 56)	50.5 (39, 59)
Male (No.)	80% (8)	70% (7)
HBV DNA level (IU/mL)		
$\leq 2\times10^2$	10% (1)	90% (9)
2×10^2-2×10^6	80% (8)	0% (0)
$>2\times10^6$	10% (1)	10% (1)
Laboratory data		
Alanine aminotransferase (U/L)	110.5 (55.5, 253.8) **, P=0.0083	33.5 (16.0, 73.8)
Aspartate aminotransferase (U/L)	167.5 (66.3, 364.5) **, P=0.0027	33.0 (23.8, 68.8)
Albumin (g/L)	35.8 (31.0, 38.9) n.s., P=0.9725	33.1 (28.7, 42.8)
Total bilirubin (μmol/L)	526.9 (386.8, 641.2) ****, P＜0.0001	27.4 (19.6, 61.5)
Creatinine (μmol/L)	52.0 (36.5, 65.0) n.s., P=0.3646	56.0 (48.8, 74.3)
Sodium (mmol/L)	136.0 (129.3, 141.0) **, P=0.0060	142.5 (141.5, 144.3)
White blood cell count (10^9/L)	5.3 (4.0, 8.3) ***, P=0.0002	2.8 (2.2, 3.9)
Platelet count (10^9/L)	67.0 (47.0, 96.8) n.s., P=0.1469	43.0 (28.5, 76.0)
Haemoglobin (g/L)	102.5 (98.5, 112.3) n.s., P=0.7668	109.5 (79.5, 129.5)
INR	2.5 (1.9, 2.7) ****, P＜0.0001	1.4 (1.2, 1.5)
Severity score		
MELD	29.5 (25.0, 31.3) ****, P＜0.0001	11.5 (10.5, 16.3)
MELD-Na	31.0 (25.8, 36.5) ****, P＜0.0001	11.5 (10.5, 16.3)

Data are presented as medians (P25, P75) or percentages (number of patients). Statistical significance was calculated using two-sided Mann-Whitney U tests for alanine aminotransferase, aspartate aminotransferase, total bilirubin, white blood cell count and MELD-Na; two-sided unpaired Student's t-tests for albumin, creatinine, platelet count and MELD; and two-sided unpaired Welch's t-tests for sodium, haemoglobin and INR. Statistical significance is indicated as follows: ****$P < 0.0001$, ***$P < 0.001$, **$P < 0.01$, *$P < 0.05$, *n.s.*, not significant. INR, international normalized ratio; MELD-Na, Model for End-Stage Liver Disease Sodium.

Table S4: REAGENT and Primer sequences used in the experiments			
**Antibodies**			
All primary antibodies used in this study were commercially obtained and validated by the manufacturers for the indicated applications. Validation information is available on the manufacturers' websites.			
**REAGENT or RESOURCE**	**SOURCE**	**IDENTIFIER**	**Working Dilution**
HNF4α (mIHC)	Cell Signaling Technology	Cat# 3113	1:1500
HepPar-1 (mIHC)	Cell Signaling Technology	Cat# 62042	1:500
HepPar-1 (mIHC)	Abcam	Cat# ab129076	1:100
CK19 (mIHC)	Proteintech	Cat# 10712-1-AP	1:3000
CD68 (mIHC)	Cell Signaling Technology	Cat# 97778	1:600
TREM2 (mIHC)	Affinity Biosciences	Cat# DF12529	1:200
TREM2 (mIHC)	Cell Signaling Technology	Cat# 55739	1:1000
CD9 (mIHC)	Affinity Biosciences	Cat# AF5139	1:200
CD9 (mIHC)	Cell Signaling Technology	Cat# 98327	1:100
GPNMB (mIHC)	Cell Signaling Technology	Cat# 38313	1:200
ACOD1 (mIHC)	Affinity Biosciences	Cat# DF7723	1:200
ACOD1 (WB)	Cell Signaling Technology	Cat# 17805	1:1000
F4/80 (mIHC)	Cell Signaling Technology	Cat# 70076	1:200
EP300 (mIHC)	Active Motif	Cat# 61401	1:500
EP300 (mIHC)	Affinity Biosciences	Cat# AF5360	1:200
EP300 (WB)	Cell Signaling Technology	Cat# 67621	1:1000
EP300 (CUT&Tag)	Cell Signaling Technology	Cat# 57625	1:50
H3K27Ac (ChIP)	Cell Signaling Technology	Cat# 8173	1:100
GAPDH (WB)	Cell Signaling Technology	Cat# 5174	1:5000
Fixable Viability Stain 780	BD Horizon™	Cat# 565388	1:1000
PerCP-Cy™5.5 Rat Anti-Mouse CD45	BD Horizon™	Cat# 550994	0.5ul/test
FITC Rat Anti-Mouse CD45	BD Horizon™	Cat# 553079	0.5ul/test
FITC anti-mouse/human CD11b	BioLegend	Cat# 101206	0.5ul/test
Brilliant Violet 785™ anti-mouse/human CD11b	BioLegend	Cat# 101243	0.5ul/test
Brilliant Violet 510™ anti-mouse F4/80	BioLegend	Cat# 123135	0.5ul/test
RB780 Rat Anti-Mouse F4/80	BD Horizon™	Cat# 569223	0.5ul/test
PE Rat Anti-Mouse F4/80	BD Horizon™	Cat# 565410	0.5ul/test
PE anti-TREM-2	BioLegend	Cat# 824806	0.5ul/test
Human/Mouse TREM2 APC-conjugated Antibody	R&D Systems	Cat# FAB17291A	0.5ul/test
PE/Cyanine7 anti-mouse	BioLegend	Cat# 124816	0.5ul/test

CD9			
Brilliant Violet 785™ anti-mouse TNF-α	BioLegend	Cat# 506341	0.5ul/test
IL-6 Monoclonal Antibody Brilliant Violet™ 650	eBioscience™	Cat# 416-7061-80	0.5ul/test
Brilliant Violet 421™ anti-mouse IL-10	BioLegend	Cat# 505022	0.5ul/test
APC anti-mouse CD195 (CCR5)	BioLegend	Cat# 107012	0.5ul/test
Brilliant Violet 650™ anti-mouse CD183 (CXCR3)	BioLegend	Cat# 126531	0.5ul/test
BB700 Rat Anti-Mouse CD182 (CXCR2)	BD Horizon™	Cat# 747809	0.5ul/test
Brilliant Violet 421™ anti-mouse CD193 (CCR3)	BioLegend	Cat# 144517	0.5ul/test
FITC Mouse Anti-Mouse CD45.1	BD Horizon™	Cat# 561871	0.5ul/test
PerCP-Cy™5.5 Mouse Anti-Mouse CD45.2	BD Horizon™	Cat# 552950	0.5ul/test
Brilliant Violet 785™ anti-human CD11B	BioLegend	Cat# 301346	0.5ul/test
APC anti-human CD68	BioLegend	Cat# 333810	0.5ul/test
Brilliant Violet 510™ anti-human CD14	BioLegend	Cat# 367124	0.5ul/test
PerCP/Cyanine5.5 anti-human CD9	BioLegend	Cat# 312110	0.5ul/test
TREM-2 (B-3) PE	SantaCruz	Cat# sc-373828PE	0.5ul/test

**Chemicals, peptides, and recombinant proteins**		
**REAGENT or RESOURCE**	**SOURCE**	**IDENTIFIER**
RIPA buffer	Beyotime	Cat# P0013B
Fetal Bovine Serum	Gibco	Cat# 10091-148
EZ Trans	Shanghai Life-iLab Biotech	Cat# AC04L092
SYBR Green	Vazyme Biotech Co., Ltd	Cat# Q221-01
Opti-MEM	Thermo Fisher Scientific	Cat# 31985070
Ficoll- Hypaque Plus	GE Healthcare	Cat# 17144003
PERCOLL	GE Healthcare	Cat# 17089109
Bovine Serum Albumin	MedChemExpress	Cat# HY-D0842
Protein A/G agarose beads	MedChemExpress	Cat# HY-K0202
Protease Inhibitor Cocktail	MedChemExpress	Cat# HY-K0010
Deacetylase Inhibitor Cocktail	MedChemExpress	Cat# HY-K0030
PEG300	MedChemExpress	Cat# HY-Y0873

DMSO	MedChemExpress	Cat# HY-Y0320
Phosphate-Buffered Saline	MedChemExpress	Cat# HY-K3005
Olive oil	MedChemExpress	Cat# HY-108749
CCS1477	MedChemExpress	Cat# HY-111784
NEO2734	MedChemExpress	Cat# HY-136938
GNE207	MedChemExpress	Cat# HY-120028
Imipenem	MedChemExpress	Cat# HY-B1369A
AntiFade Mounting Medium (with DAPI)	MedChemExpress	Cat# HY-K1047
Penicillin-Streptomycin (100×), Suitable for Cell Culture	Yeasen, Shanghai, China	Cat# 60162ES
TS-GelRed Nucleic Acid Gel Stain	Beijing Tsingke Biotech Co., Ltd	Cat# TSJ002
Recombinant Murine GM-CSF	PeproTech	Cat# 315-03-50UG
Recombinant Human TGF-beta1 (HEK293 derived)	PeproTech	Cat# 100-21-100UG
**Critical commercial assays**		
**REAGENT or RESOURCE**	**SOURCE**	**IDENTIFIER**
BCA Protein Assay kit	Yeasen	Cat# 20201ES96
FastPure®Cell/Tissue Total RNA Isolation Kit V2	Vazyme Biotech Co., Ltd	Cat# RC112
pHrodo™ Green *E. coli* BioParticles™ conjugates for phagocytosis	Thermo Fisher Scientific	Cat# P35366
pHrodo™ RED *E. coli* BioParticles™ conjugates for phagocytosis	Thermo Fisher Scientific	Cat# P35361
oxidative burst quantitative assay kit	Absin Bioscience	Cat# abS70002
ChIP kit	Cell Signaling Technology	Cat# 9005
**Animal**		
**REAGENT or RESOURCE**	**SOURCE**	**IDENTIFIER**
Mouse: WT: C57BL/6J	Beijing HFK Bio-Technology Co., Ltd	N/A
Mouse: C57BL/6J-*Acod1*^{em1Cflox}/Cya	Cyagen Biosciences (Suzhou) Inc.	N/A

**shRNA sequences used for *Ep300* gene knockdown**		
sh *Ep300*#1	CGCGAATGACAACACAGATTTCTCGAGAAATCTGTGTTGTCATTCGCGTTTT	
sh *Ep300*#2	TAACTCTGGCCATAGCTTAATCTCGAGATTAAGCTATGGCCAGAGTTATTTT	
**RT-qPCR primers**		
GAPDH	F: ACAACTTTGGTATCGTGGAAGG	R: GCCATCACGCCACAGTTTC
ACOD1	F: TTCCATGAATGCCAGATCAA	R: GGTTTTCTCCAGTGCCCATA
EP300	F: TCTTCAGCACCATGGACAGT	R: GTTGCATACGAGGCCCATAG
Gapdh	F: GGTGAAGGTCGGTGTGAACGGA	R: CCAAAGTTGTCATGGATGACCTTGG
Acod1	F: GCGAACGCTGCCACTCA	R: ATCCCAGGCTTGGAAGGTC
Ep300	F: GGGACTAACCAATGGTGGTG	R: ATTGGGAGAAGTCAAGCCTG
**Genotyping validation primers for mouse conditional knockout *Acod1***		
Acod1 Primers	F: TACCTGTGGATTGAAGAGCTTGAT	R: GAAGGAAGGACATTGTGGTTTGTG
Lyz2-Cre Primers	F: CCCAGAAATGCCAGATTACG	R: CTTGGGCTGCCAGAATTTCTC
