# Supplementary material for: EP300 inhibition alleviates acute-on-chronic liver failure through modulation of scar-associated macrophages
Source: Nat Commun. 2026 Oct 7;17:11432. doi: 10.1038/s41467-026-78245-x (PMC13737046; doi:10.1038/s41467-026-78245-x)
Supplement: Supplementary file 2 [file 41467_2026_78245_MOESM2_ESM.pdf]

nature portfolio

Corresponding author(s): Xiong Ma, Yugang Wang, Yuanyuan Zhang, Min Shi, Cheng Luo

Last updated by author(s): Jul 15, 2026

Reporting Summary

Nature Portfolio wishes to improve the reproducibility of the work that we publish. This form provides structure for consistency and transparency in reporting. For further information on Nature Portfolio policies, see our Editorial Policies and the Editorial Policy Checklist.

Statistics

For all statistical analyses, confirm that the following items are present in the figure legend, table legend, main text, or Methods section.

n/a	Confirmed	
☐	☒	The exact sample size (*n*) for each experimental group/condition, given as a discrete number and unit of measurement
☐	☒	A statement on whether measurements were taken from distinct samples or whether the same sample was measured repeatedly
☐	☒	The statistical test(s) used AND whether they are one- or two-sided<br>*Only common tests should be described solely by name; describe more complex techniques in the Methods section.*
☒	☐	A description of all covariates tested
☐	☒	A description of any assumptions or corrections, such as tests of normality and adjustment for multiple comparisons
☐	☒	A full description of the statistical parameters including central tendency (e.g. means) or other basic estimates (e.g. regression coefficient) AND variation (e.g. standard deviation) or associated estimates of uncertainty (e.g. confidence intervals)
☐	☒	For null hypothesis testing, the test statistic (e.g. *F*, *t*, *r*) with confidence intervals, effect sizes, degrees of freedom and *P* value noted<br>*Give P values as exact values whenever suitable.*
☒	☐	For Bayesian analysis, information on the choice of priors and Markov chain Monte Carlo settings
☒	☐	For hierarchical and complex designs, identification of the appropriate level for tests and full reporting of outcomes
☐	☒	Estimates of effect sizes (e.g. Cohen's *d*, Pearson's *r*), indicating how they were calculated

Our web collection on statistics for biologists contains articles on many of the points above.

Software and code

Policy information about availability of computer code

Data collection	Commercial software supplied by the instrument manufacturers was used for data collection. No custom software was used.
Data analysis	Data were analysed using publicly available software and packages as described in the methods section. No custom algorithms were developed for this study.

For manuscripts utilizing custom algorithms or software that are central to the research but not yet described in published literature, software must be made available to editors and reviewers. We strongly encourage code deposition in a community repository (e.g. GitHub). See the Nature Portfolio guidelines for submitting code & software for further information.

Data

Policy information about availability of data

All manuscripts must include a data availability statement. This statement should provide the following information, where applicable:

- Accession codes, unique identifiers, or web links for publicly available datasets
- A description of any restrictions on data availability
- For clinical datasets or third party data, please ensure that the statement adheres to our policy

A Data Availability statement has been included in the manuscript. All newly generated sequencing datasets have been deposited in GEO and are publicly available under accession codes GSE338157, GSE338158 and GSE338159. Previously published datasets used in this study are cited with their corresponding accession codes and hyperlinks. Source data are provided with this paper.

Research involving human participants, their data, or biological material

Policy information about studies with human participants or human data. See also policy information about sex, gender (identity/presentation), and sexual orientation and race, ethnicity and racism.

Reporting on sex and gender	Sex was recorded from participants' medical records and both male and female participants were included in all cohorts. Sex was not considered as a biological variable in the study design or statistical analyses.
Reporting on race, ethnicity, or other socially relevant groupings	All data used in this study were derived from Asian populations.
Population characteristics	These information are mentioned in Table S1-S3.
Recruitment	Participants were recruited at Renji Hospital according to predefined inclusion and exclusion criteria. Written informed consent was obtained from all participants. No participant compensation was provided and no self-selection bias was identified.
Ethics oversight	The ethics committee approval number for this study is KY2020-055 and KY2021-063-B.

Note that full information on the approval of the study protocol must also be provided in the manuscript.

Field-specific reporting

Please select the one below that is the best fit for your research. If you are not sure, read the appropriate sections before making your selection.

☒ Life sciences ☐ Behavioural & social sciences ☐ Ecological, evolutionary & environmental sciences

For a reference copy of the document with all sections, see nature.com/documents/nr-reporting-summary-flat.pdf

Life sciences study design

All studies must disclose on these points even when the disclosure is negative.

Sample size	Sample size was determined based on previous studies. Sample sizes were mentioned in methods, figures and figure legends.
Data exclusions	No data were excluded from the analysis.
Replication	Experiments for which statistical analyses were performed were independently repeated at least three times with similar results.
Randomization	Following liver fibrosis induction, mice were stratified by body weight and randomly assigned to experimental groups using a random number table.
Blinding	Outcome assessment of the experimental data was performed in a blinded manner.

Behavioural & social sciences study design

All studies must disclose on these points even when the disclosure is negative.

Study description	
Research sample	
Sampling strategy	
Data collection	
Timing	
Data exclusions	
Non-participation	
Randomization	

Ecological, evolutionary & environmental sciences study design

All studies must disclose on these points even when the disclosure is negative.

Study description	
Research sample	
Sampling strategy	
Data collection	
Timing and spatial scale	
Data exclusions	
Reproducibility	
Randomization	
Blinding	

Did the study involve field work? ☐ Yes ☐ No

Field work, collection and transport

Field conditions	
Location	
Access & import/export	
Disturbance	

Reporting for specific materials, systems and methods

We require information from authors about some types of materials, experimental systems and methods used in many studies. Here, indicate whether each material, system or method listed is relevant to your study. If you are not sure if a list item applies to your research, read the appropriate section before selecting a response.

Materials & experimental systems

n/a	Involved in the study	
☐	☒	Antibodies
☐	☒	Eukaryotic cell lines
☒	☐	Palaeontology and archaeology
☐	☒	Animals and other organisms
☐	☒	Clinical data
☒	☐	Dual use research of concern
☒	☐	Plants

Methods

n/a	Involved in the study	
☐	☒	ChIP-seq
☐	☒	Flow cytometry
☒	☐	MRI-based neuroimaging

Antibodies

Antibodies used	All primary and secondary antibodies used in this study are listed in Supplementary Table S4.
Validation	All antibodies were validated by the manufacturers and further confirmed by preliminary experiments .

Eukaryotic cell lines

Policy information about cell lines and Sex and Gender in Research

Cell line source(s)	The relevant information is provided in the Methods.
Authentication	All cells were deteceded by STR.
Mycoplasma contamination	Negative.
Commonly misidentified lines (See ICLAC register)	None.

Palaeontology and Archaeology

Specimen provenance	
Specimen deposition	
Dating methods	

☐ Tick this box to confirm that the raw and calibrated dates are available in the paper or in Supplementary Information.

Ethics oversight	

Note that full information on the approval of the study protocol must also be provided in the manuscript.

Animals and other research organisms

Policy information about studies involving animals; ARRIVE guidelines recommended for reporting animal research, and Sex and Gender in Research

Laboratory animals	The relevant information is provided in the Methods
Wild animals	None.
Reporting on sex	The relevant information is provided in the Methods
Field-collected samples	The relevant information is provided in the Methods
Ethics oversight	All animal experiments were approved by the Institutional Animal Care and Use Committee (IACUC) of the Shanghai Institute of Materia Medica, Chinese Academy of Sciences.

Note that full information on the approval of the study protocol must also be provided in the manuscript.

Clinical data

Policy information about clinical studies

All manuscripts should comply with the ICMJE guidelines for publication of clinical research and a completed CONSORT checklist must be included with all submissions.

Clinical trial registration	-
Study protocol	-
Data collection	-
Outcomes	-

Dual use research of concern

Policy information about dual use research of concern

Hazards

Could the accidental, deliberate or reckless misuse of agents or technologies generated in the work, or the application of information presented in the manuscript, pose a threat to:

No	Yes	
☐	☐	Public health
☐	☐	National security
☐	☐	Crops and/or livestock
☐	☐	Ecosystems
☐	☐	Any other significant area

Experiments of concern

Does the work involve any of these experiments of concern:

No	Yes	
☐	☐	Demonstrate how to render a vaccine ineffective
☐	☐	Confer resistance to therapeutically useful antibiotics or antiviral agents
☐	☐	Enhance the virulence of a pathogen or render a nonpathogen virulent
☐	☐	Increase transmissibility of a pathogen
☐	☐	Alter the host range of a pathogen
☐	☐	Enable evasion of diagnostic/detection modalities
☐	☐	Enable the weaponization of a biological agent or toxin
☐	☐	Any other potentially harmful combination of experiments and agents

Plants

Seed stocks	-
Novel plant genotypes	-
Authentication	-

ChIP-seq

Data deposition

☒ Confirm that both raw and final processed data have been deposited in a public database such as GEO.

☒ Confirm that you have deposited or provided access to graph files (e.g. BED files) for the called peaks.

Data access links<br>*May remain private before publication.*	https://www.ncbi.nlm.nih.gov/geo/query/acc.cgi?acc=GSE338158<br>https://www.ncbi.nlm.nih.gov/geo/query/acc.cgi?acc=GSE338159
Files in database submission	Raw FASTQ files, narrowPeak files and metadata.
Genome browser session<br>(e.g. UCSC)	No longer applicable.

Methodology

Replicates	Three independent biological replicates were generated for each experimental group and pooled before ChIP-seq library preparation and sequencing.
Sequencing depth	Paired-end 150-bp sequencing was performed on an Illumina NovaSeq 6000 platform.
Antibodies	The relevant information is fully described in the table S4.
Peak calling parameters	Reads were aligned to the mm10_gencode reference genome using BWA. Peaks were called using MACS2 with default parameters.
Data quality	Only high-quality sequencing data passing standard quality-control procedures were used for downstream analyses.

Software | Bowtie2, SAMtools, deepTools and MACS2 were used for data analysis. No custom software was used.

Flow Cytometry

Plots

Confirm that:

☒ The axis labels state the marker and fluorochrome used (e.g. CD4-FITC).

☒ The axis scales are clearly visible. Include numbers along axes only for bottom left plot of group (a 'group' is an analysis of identical markers).

☒ All plots are contour plots with outliers or pseudocolor plots.

☒ A numerical value for number of cells or percentage (with statistics) is provided.

Methodology

Sample preparation | The relevant information is fully described in the Methods section.

Instrument | The relevant information is fully described in the Methods section.

Software | flowjo.

Cell population abundance | The relevant information is fully described in the Methods section.

Gating strategy | The relevant information is fully described in Supplementary Figures S15-S17.

☒ Tick this box to confirm that a figure exemplifying the gating strategy is provided in the Supplementary Information.

Magnetic resonance imaging

Experimental design

Design type

Design specifications

Behavioral performance measures

Acquisition

Imaging type(s)

Field strength

Sequence & imaging parameters

Area of acquisition

Diffusion MRI ☐ Used ☐ Not used

Preprocessing

Preprocessing software

Normalization

Normalization template

Noise and artifact removal

Volume censoring

Statistical modeling & inference

Model type and settings

Effect(s) tested

Specify type of analysis: ☐ Whole brain ☐ ROI-based ☐ Both

Statistic type for inference

(See Eklund et al. 2016)

Correction

Models & analysis

n/a	Involved in the study
☐	☐ Functional and/or effective connectivity
☐	☐ Graph analysis
☐	☐ Multivariate modeling or predictive analysis

Functional and/or effective connectivity

Graph analysis

Multivariate modeling and predictive analysis
