# Supplementary material for: EP300 inhibition alleviates acute-on-chronic liver failure through modulation of scar-associated macrophages
Source: Nat Commun. 2026 Oct 7;17:11432. doi: 10.1038/s41467-026-78245-x (PMC13737046; doi:10.1038/s41467-026-78245-x)
Supplement: Supplementary file 3 [file 41467_2026_78245_MOESM3_ESM.pdf]

nature portfolio

Peer Review File

EP300 inhibition alleviates acute-on-chronic liver failure through modulation of scar-associated macrophages

Corresponding Author: Professor Cheng Luo

This file contains all reviewer reports in order by version, followed by all author rebuttals in order by version.

Version 0:

Reviewer comments:

Reviewer #1

(Remarks to the Author)
Chen P. et al. presented an intriguing study on the potential role of scar-associated macrophages (SAMs) in liver tissue. The authors demonstrate that EP300 regulates inflammation and injury, likely through modulating Acod1 expression, which in turn influences phagocytosis and bacterial clearance.
While the study addresses an important topic and provides valuable insights, there are several critical issues that warrant further consideration and clarification.

Figure 1G:
The orientation of Figure 1G is challenging to interpret. Including hepatocyte and cholangiocyte markers would improve clarity regarding tissue structure and help determine whether macrophages are primarily located in necrotic areas or associated with preserved tissue islands.
Since SAMs are reported to correlate with disease severity, their specific tissue localization needs to be clarified. Are SAMs confined to necrotic regions? Necrosis is a hallmark of HBV-associated ACLF, which differs from the alcohol- and infection-triggered ACLF commonly seen in Europe. If SAMs are localized only in necrotic areas, their impact on non-necrotic regions —potentially distant from their site of action—remains unclear. This highlights the need for more advanced spatial analyses of tissue localization.
Animal Models:
Similar tissue localization studies should be performed in the animal models, which are distinct from HBV-driven ACLF both etiologically and phenotypically. Greater detail on SAM localization within the tissue and their effects on remaining healthy tissue is needed.
ACLF Cohort:
Clarification is required regarding the origin and etiology of the ACLF cohort for liver and PBMC analysis. Do these livers exhibit submassive necrosis, suggesting HBV-driven ACLF? Providing this context is crucial for interpretation.
Animal Model Results:
The authors employed a CCl4 chronic + CCl4 acute + Klebsiella model. How do the findings on EP300 expression and SAM infiltration align with human observations of submassive necrosis in HBV? While CCl4 induces necrosis, Klebsiella elicits an inflammatory response. Understanding EP300 localization and the proposed mechanism of its action is essential.
Figure 2:
The claim that EP300 is expressed in $Trem2^{+}Cd9^{+}$ cells conflicts with the images, which suggest EP300 colocalization with $Cd9^{+}$ but not with F4/80 or Trem2. This discrepancy needs to be addressed. Additionally, arrows pointing to cells within vessels likely represent artifacts and should be clarified or removed. (See also Figure 3 for SAMs: $F4/80^{+}Trem2^{+}Cd9^{+}$.)
If EP300 is predominantly located in $Cd9^{+}$ cells, what is the proposed mechanism by which EP300 inhibition reduces SAMs? Is this a direct effect on their phenotype, or is it an indirect consequence of reduced tissue injury and subsequent recruitment?
Mechanism of EP300 in Injury:
The mechanism by which EP300 modulates injury requires further explanation. While inhibition of EP300 reduces injury and SAMs, it remains unclear whether this occurs through direct effects on hepatocytes or alternative pathways.
Terminology:
Avoid the term "necroptotic apoptosis." Specify whether necroptosis or apoptosis is being referred to.
Acod1 Knockout:
Acod1 knockout animals showed prolonged survival in ACLF models, with reduced SAMs, less injury, and lower bacterial

loads. Since Acod1 regulates phagocytosis, these effects are hypothesized to result from diminished phagocytic activity. However, if this hypothesis is correct, Acod1 knockout might not affect the CCl4 model. This raises questions about its relevance for HBV-ACLF patients without infection.
EP300 Inhibition and Acod1:
EP300 inhibition via CCS1477 transcriptionally suppresses Acod1, but this is perplexing given that EP300 does not appear to be expressed in true SAMs. Clarification of this mechanism is necessary.
Acod1Mye$^{-/-}$ Mouse Model:
The use of Acod1Mye$^{-/-}$ mice complicates interpretation as the knockout impacts the entire model, including the chronic CCl4 phase. Analyzing the effects in both the chronic phase and the combined CCl4 chronic + acute phase is critical for clearer insights.
Treatment Regimens:
Additional details on the treatment regimens for CCS1477 and other compounds are needed. Were these administered as single or multiple injections? What was the treatment duration, number of injections, and timing relative to the model (e.g., before acute CCl4 or before Klebsiella)?

Reviewer #2

(Remarks to the Author)
In this manuscript, Chen et al. demonstrated that scar-associated macrophages (SAMs) are increased in the livers of acute-on-chronic liver failure (ACLF) both in humans and murine model. They identified EP300 as the most prominent upstream regulator mediating the expression of the transcription factors required for the differentiation SAMs of ACLF. They also demonstrated that ACOD1 expression is induced in ACLF SAMs and its loss mitigates hepatic destruction and extends ACLF mouse survival via enhanced phagocytosis of SAMs. Overall, the data are presented well and the results are potentially interesting, however, there are critical concerns to be addressed.
Major concerns
1. Although the authors showed the increased number of SAMs in the liver of ACLF and its association with the disease progression, it is unclear how these cells directly contribute to the progression of ACLF. Information on cytokine production and cytotoxicity of SAMs is missing, and in addition to the numbers, it is necessary to show whether their characteristics differ from those of liver fibrosis models. Furthermore, the direct involvement of this cell subset in the progression of ACLF must be demonstrated by depleting these cells both in liver fibrosis and ACLF mouse models.
2. Although pharmacological inhibition of EP300 was shown to reduce the hepatic damage of ACLF via removal of SAM (Figure 3), it remains to be shown whether inhibition of EP300 affects other subsets of CD11b+F480+ macrophages in the liver.
3. Related to 2, it is possible that EP300 can program the transcription of immune cells other than liver cells during ACLF. Indeed, a recent paper reported an Ep300-dependent transcriptional program regulating intestinal epithelial cell (IEC) activation during acute liver failure (Cell Discov. 2023 DOI: 10.1038/s41421-023-00578-4). Since it has been suggested that bacterial translocation is involved in the pathogenesis of ACLF, the authors should clarify this point.
Minor concerns
1. In all flowcytometry data, please also show the representative FACS data.
2. Please show longitudinal changes in serum transaminase and liver tissue in the ACLF model administered with Klebsiella pneumoniae compared to the steady state, liver fibrosis model.

Version 1:

Reviewer comments:

Reviewer #1

(Remarks to the Author)
I would like to congratulate the authors for their effort in improving the manuscript. The revised version indeed shows substantial progress. However, I still have concerns regarding two major aspects: (1) the spatial organization of SAMs relative to injury sites and preserved hepatocytes, and (2) the Acod1-related mechanisms. Both elements are central to the authors' mechanistic argument and require a solid evidential basis. My comments mainly relate to the new data and newly added figures, but also reflect general considerations.
Comments
Figure R1:
The CK19 staining appears convincing. However, I have doubts regarding the HNF4a and Trem2 staining:
• HNF4a is expected to be nuclear, while the presented images show predominantly cytoplasmic staining, alternative use Hepar-1 rather than HNF4a.
• Trem2 staining appears to contain substantial background signal, with limited clear association with DAPI. I have difficulties in finding a reliable Trem2 staining.
Additionally, Trem2 seems to stain virtually all cells, including hepatocytes. Is this expected? If so, what is the functional implication of Trem2 expression in hepatocytes?
Figure R4:
HNF4a staining appears reliable at the borders of preserved hepatocyte islands. However, the Trem2 staining again raises concerns: low concordance with DAPI and apparent Trem2 positivity in some hepatocytes. If these signals are indeed specific, the data would suggest that SAMs localize predominantly to regions of ductular reactions—areas characterized by

biliary progenitor cell responses and strong CK19 expression. What is the proposed mechanism in this region?
Figure R5:
The overall staining shown in this figure appears even less distinct, with what looks like considerable nonspecific/background signal. Given this, I am not confident that the images allow firm conclusions about spatial organization of SAMs or zonal distribution.
Acod1Mye−/− Mouse Model in Chronic CCl_4 Injury:
The reported phenotype in the CCl4 chronic face is puzzling. The Acod1 knockout appears detrimental in the chronic CCl_4 model but protective in the acute injury model. What is the mechanistic explanation for this discrepancy?
Similarly, the new in vitro stimulation data (page 9, lines 300–306, and page 10, lines 307–310) are difficult to reconcile. Do SAMs from Acod1Mye−/− mice exhibit reduced or increased phagocytic capacity compared to WT? The text suggests potentially conflicting findings. How do these results align with the increased injury observed in the chronic CCl_4 model?
Additionally, in both Figure 4 and Figure S12, the combination of Acod1 KO and CCS appears not to enhance the beneficial effects of Acod1 deficiency, but instead to reduce phagocytosis. What is the authors' explanation for this unexpected result?

Reviewer #2

(Remarks to the Author)
The authors have addressed most of my concerns through extensive additional experiments. I believe the paper is much improved.

Reviewer #3

(Remarks to the Author)
In this revised paper, in response to major issues raised in the previous round of review, such as whether SAMs (scar-associated macrophages) truly drive ACLF (acute-on-chronic liver failure), the authors replaced depletion experiments with functional enhancement experiments (adoptive transfer) to circumvent technical limitations, emphasizing functional restoration rather than cell quantity. Meanwhile, to determine whether the mechanism is confined to SAMs, the authors excluded the possibility of other cell types playing a dominant role through subgroup analysis, functional validation of Acod1, and EP300 co-localization studies. Regarding whether the mouse model represents human ACLF, in the new version, it is emphasized that bacterial infection-induced ACLF accounts for two-thirds of clinical cases, and the localization and function of SAMs are consistent across species, indicating the model's translational value. Furthermore, addressing concerns about whether the EP300-ACOD1 axis has a direct relationship, the authors employed multi-level evidence, including ChIP-seq, CUT&Tag, and adoptive transfer experiments, to establish direct regulation and functional causality. Overall, the authors have made substantial improvements to the article in response to the former comments from the previous round of review.

The following lists some new comments. First, in the causality validation, the authors have not yet utilized a genetic model specifically targeting the depletion of SAMs. Therefore, the authors need to discuss the necessity of SAMs in ACLF. At present, the article only employs the CCl4 plus bacterial infection model, failing to cover other common ACLF triggers such as alcohol and drugs. Second, the theme of this study is the discovery that EP300 regulates the phagocytic function of SAMs through ACOD1, thereby influencing the progression of ACLF. The content lacks a mechanistic diagram. Although the authors have provided a schematic in the graphical abstract, it is recommended to include a pathway diagram in the main text as well. This diagram could also be used to summarize the main results of the article. The current article structure makes it difficult to discern an inherent logic easily. Third, the authors utilized data from different sources but did not rigorously examine batch effects or important information such as sample gender. This may lead to bias in the results.

Version 2:

Reviewer comments:

Reviewer #1

(Remarks to the Author)
All comments were sufficiently addressed.

I have only one minor comment:

Figure R3 contains a wrong labeling of hepatocyte marker, its labled as Hepar 1 but shows HNF4a

Open Access This Peer Review File is licensed under a Creative Commons Attribution 4.0 International License, which permits use, sharing, adaptation, distribution and reproduction in any medium or format, as long as you give appropriate credit to the original author(s) and the source, provide a link to the Creative Commons license, and indicate if changes were made.
In cases where reviewers are anonymous, credit should be given to 'Anonymous Referee' and the source.
The images or other third party material in this Peer Review File are included in the article's Creative Commons license, unless indicated otherwise in a credit line to the material. If material is not included in the article's Creative Commons license and your intended use is not permitted by statutory regulation or exceeds the permitted use, you will need to obtain permission directly from the copyright holder.
To view a copy of this license, visit https://creativecommons.org/licenses/by/4.0/

Point-by-point responses

We are very grateful to the reviewers for providing us with very helpful, consistent, and constructive feedback on our work to revise and improve our manuscript.

We have conducted several new experiments, performed new analyses, and made several modifications throughout the manuscript and its figures to address all the reviewers' concerns. We have included detailed, point-by-point responses to each of these concerns, describing the corresponding changes in our manuscript. Our responses are highlighted in blue text to facilitate the review process.

Nature Communications

Manuscript Number: NCOMMS-24-69199-T

"EP300 inhibition alleviates acute-on-chronic liver failure through modulation of scar-associated macrophages"

REVIEWER COMMENTS

Reviewer #1 (Comments to the Author):

Chen P. et al. presented an intriguing study on the potential role of scar-associated macrophages (SAMs) in liver tissue. The authors demonstrate that EP300 regulates inflammation and injury, likely through modulating Acod1 expression, which in turn influences phagocytosis and bacterial clearance.

While the study addresses an important topic and provides valuable insights, there are several critical issues that warrant further consideration and clarification.

Response: We sincerely thank the reviewer for the positive and encouraging comments on our study.

1. The orientation of Figure 1G is challenging to interpret. Including hepatocyte and cholangiocyte markers would improve clarity regarding tissue structure and help determine whether macrophages are primarily located in necrotic areas or associated with preserved tissue islands.

Since SAMs are reported to correlate with disease severity, their specific tissue localization needs to be clarified. Are SAMs confined to necrotic regions ? Necrosis is a hallmark of HBV-associated ACLF, which differs from the alcohol- and infection-triggered ACLF commonly seen in Europe. If SAMs are localized only in necrotic areas, their impact on non-necrotic regions—potentially distant from their site of action—remains unclear. This highlights the need for more advanced spatial analyses of tissue localization.

Response: We thank the reviewer for the suggestion. Based on the reviewer's suggestions, we have included the hepatocyte marker (HNF4α) and the cholangiocyte marker (CK19) in the SAM localization images **(Fig. R1)**. SAMs preferentially accumulate in periportal/potal areas rather than confined to necrotic regions **(Fig. R1, R2)**.

SAMs' distribution independent of necrosis: We apologize for the previous data presentation and unclear description that might have linked SAMs with necrotic regions. To clarify this point, we have added additional data:

Although necrotic regions extending along the central veins are a characteristic feature of HBV-ACLF[1], SAMs are sparse around central-vein necrotic zones but are enriched in and around the portal areas **(Fig. R2),** indicating that SAM localization is largely independent of the necrotic-zone distribution. Compared with liver cirrhosis (LC), ACLF livers with extensive necrosis show disruption of fibrous septa, poorly demarcated portal and lobular structures, and broader SAM infiltration around the portal areas **(Fig. R3)**.

SAMs' distribution independent of etiology: SAMs were primarily enriched in the portal areas surrounding collagen-positive scar regions. This pattern was observed in non-HBV cirrhosis[2] and confirmed in both HBV-related **(Fig. R1-R3)** and alcohol-related ACLF livers **(Fig. R4)**.

SAMs' localization suggests their function: Studies have shown that in bacterial infection models, Kupffer cells located around the portal vein play a key role in protecting the host from bacterial spread and sepsis[3], which may suggest a related role for SAMs. Based on our study, the positive correlation between increased numbers of SAMs and ACLF disease severity may reflect that a higher SAM count indicates more severely impaired antibacterial immune function.We have now included this result in the revised manuscript **(page 3-4, lines124-137；Fig. S3；New data Fig. S4-S6)**.

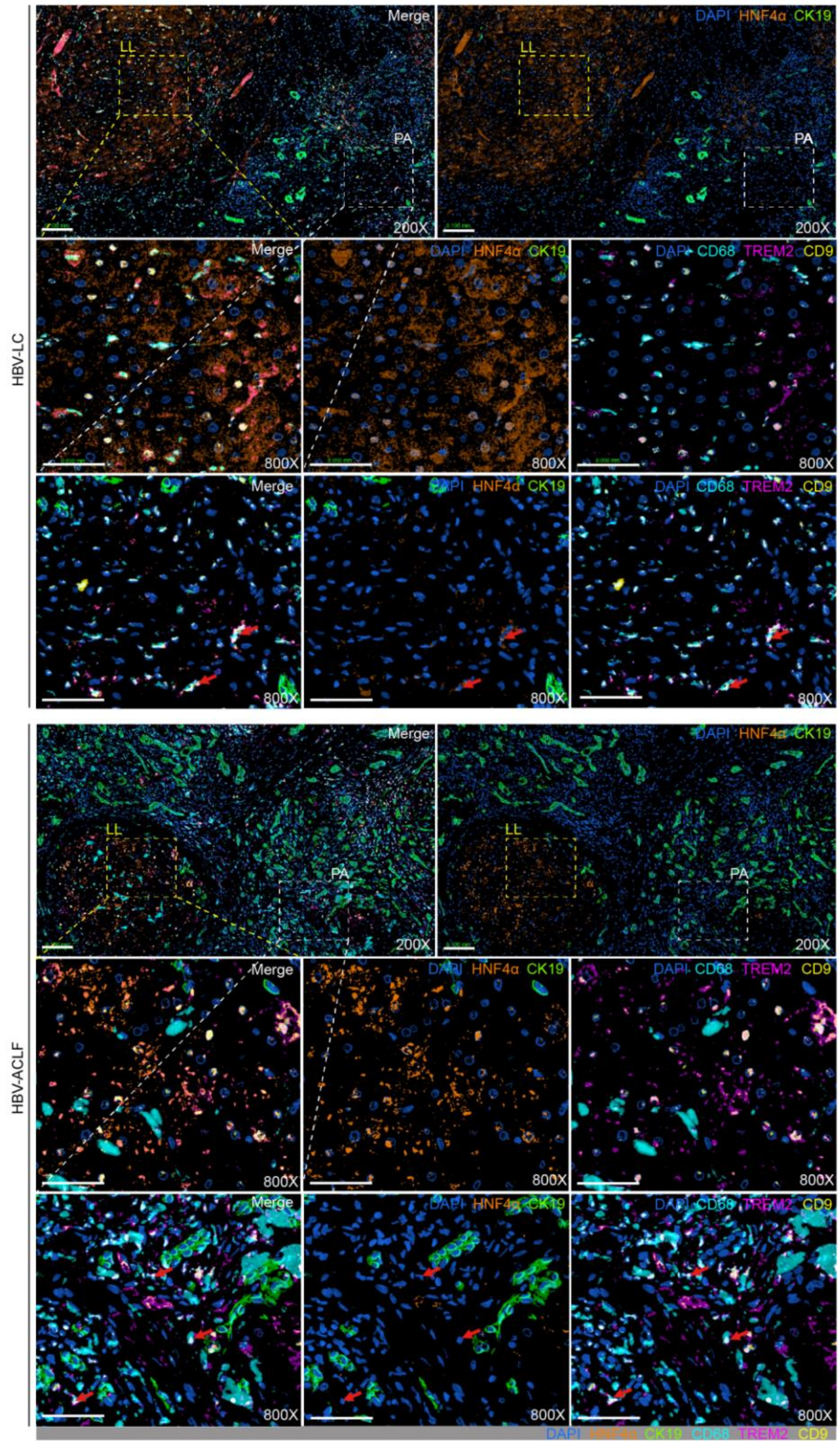

Figure R1. SAMs infiltration sites in HBV-related LC and HBV-related ACLF patients. Related to Figure S4 (New data). Representative mIHC images of the patients showing both the liver lobular (LL) and portal areas (PA). Hepatocyte marker (HNF4α) and cholangiocyte marker (CK19) were used to identify liver structures, while CD68, TREM2, and CD9 were used to identify SAMs (Red arrows). Scale bars, 0.1 mm at 200x and 0.05 mm at 800x. LC, liver cirrhosis; ACLF, Acute-on-Chronic Liver Failure.

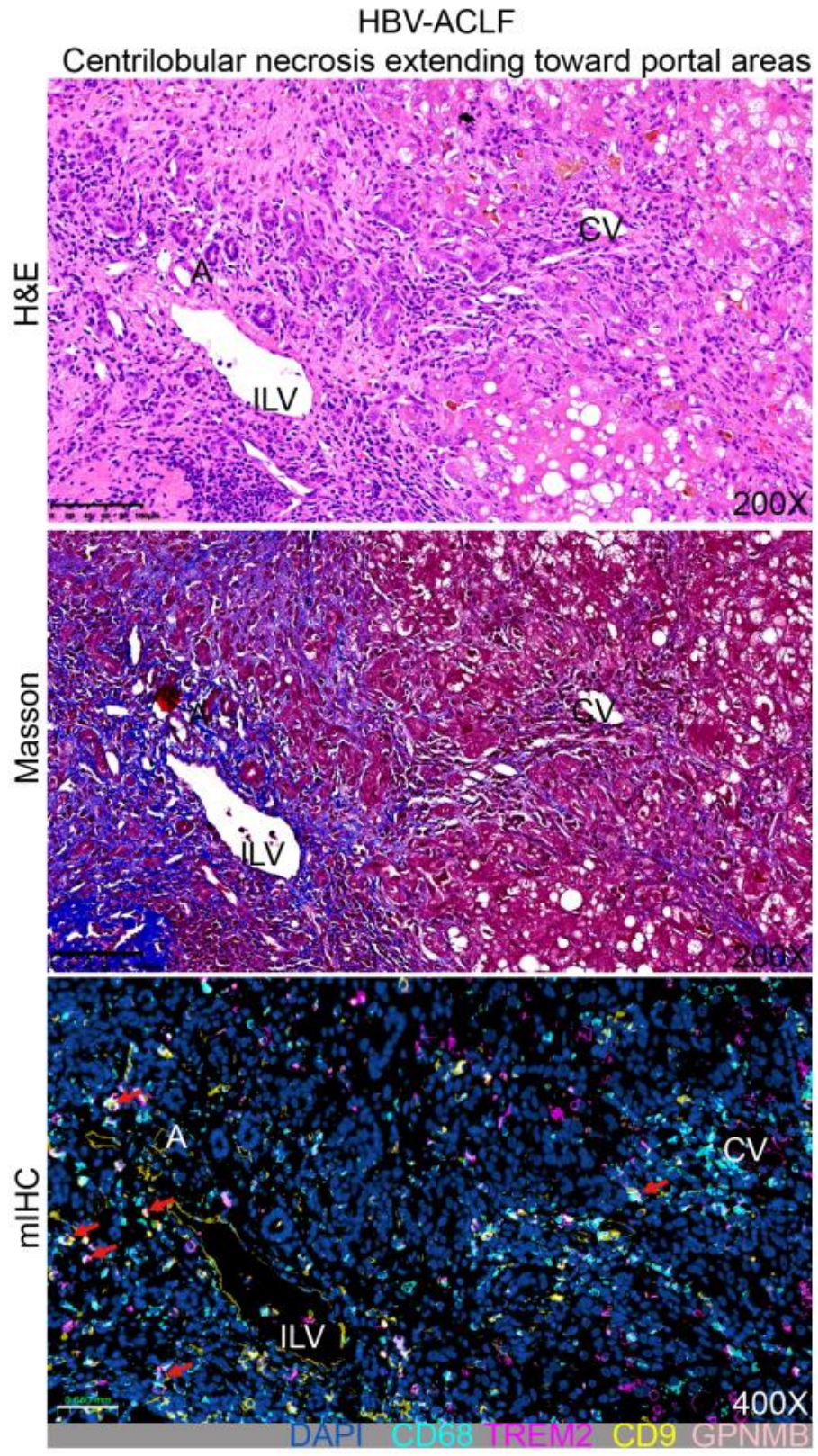

Figure R2. Pathological characteristics and SAMs infiltration sites in HBV-related ACLF patients. Related to Figure S6 (New data).

In the liver of HBV-ACLF patient, centrilobular necrosis originates from the central vein (CV) and extends toward the portal area (PA), showing blurred boundaries between necrotic and periportal regions. SAMs (red arrows) were enriched in and around the PA, identified using CD68, TREM2, CD9, and GPNMB. Scale bars: 0.1 mm at 200× and 0.05 mm at 400×. A, artery; ILV, interlobular vein.

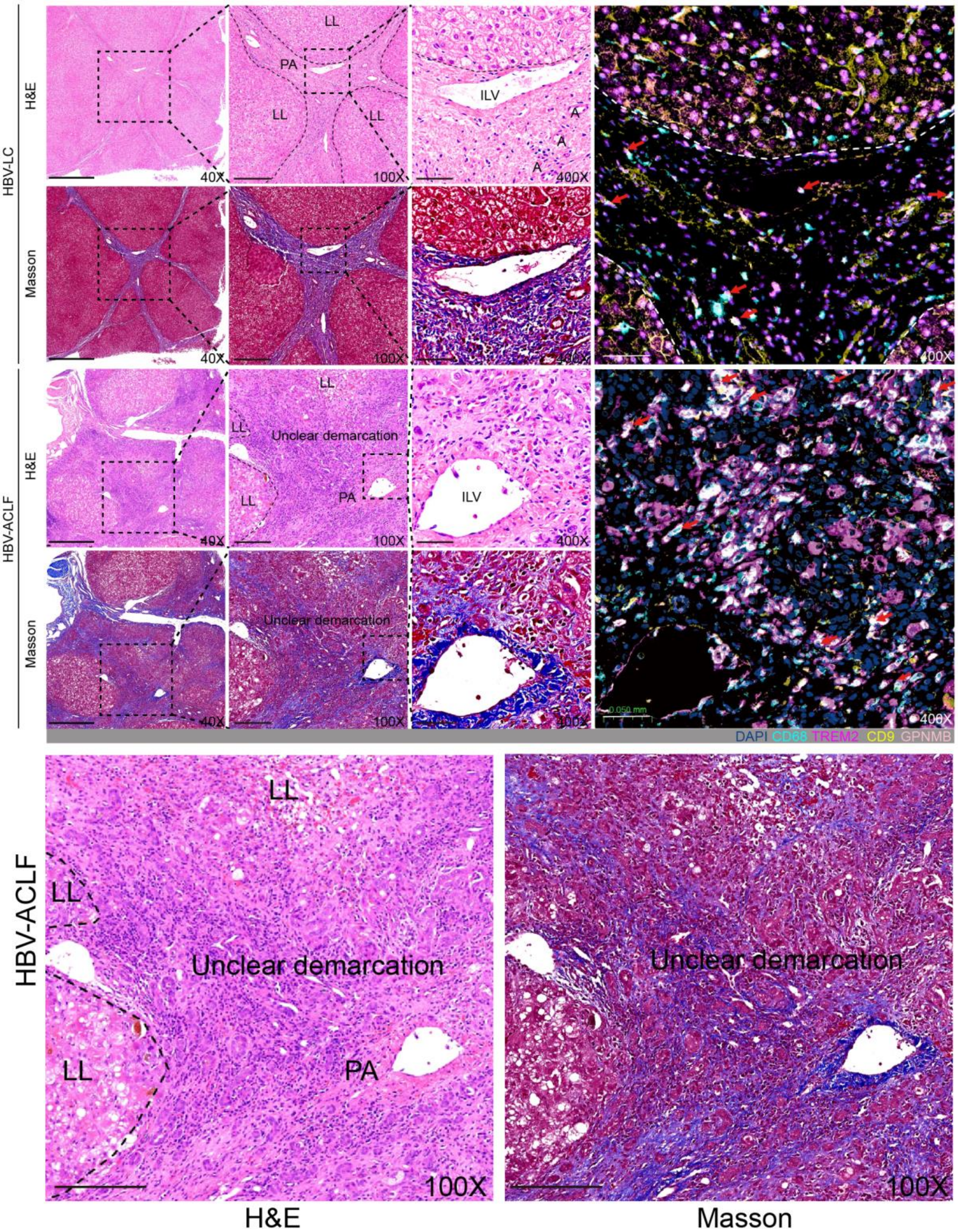

Figure R3. Pathological characteristics and SAMs infiltration sites in HBV-related LC and HBV-related ACLF patients. Related to Figure S3.

Representative H&E staining, Masson trichrome, and mIHC images of the patient's liver. Compared with LC, ACLF livers show disruption of fibrous septa, poorly demarcated portal and lobular structures, and broader SAM infiltration around the portal areas. Scale bars: 0.625 mm at 40×, 0.2 mm at 100×, and 0.05 mm at 400×. SAMs (red arrows) were identified using CD68, TREM2, CD9, and GPNMB. PA, portal area; LL, liver lobe; ILV, interlobular vein; A, artery.

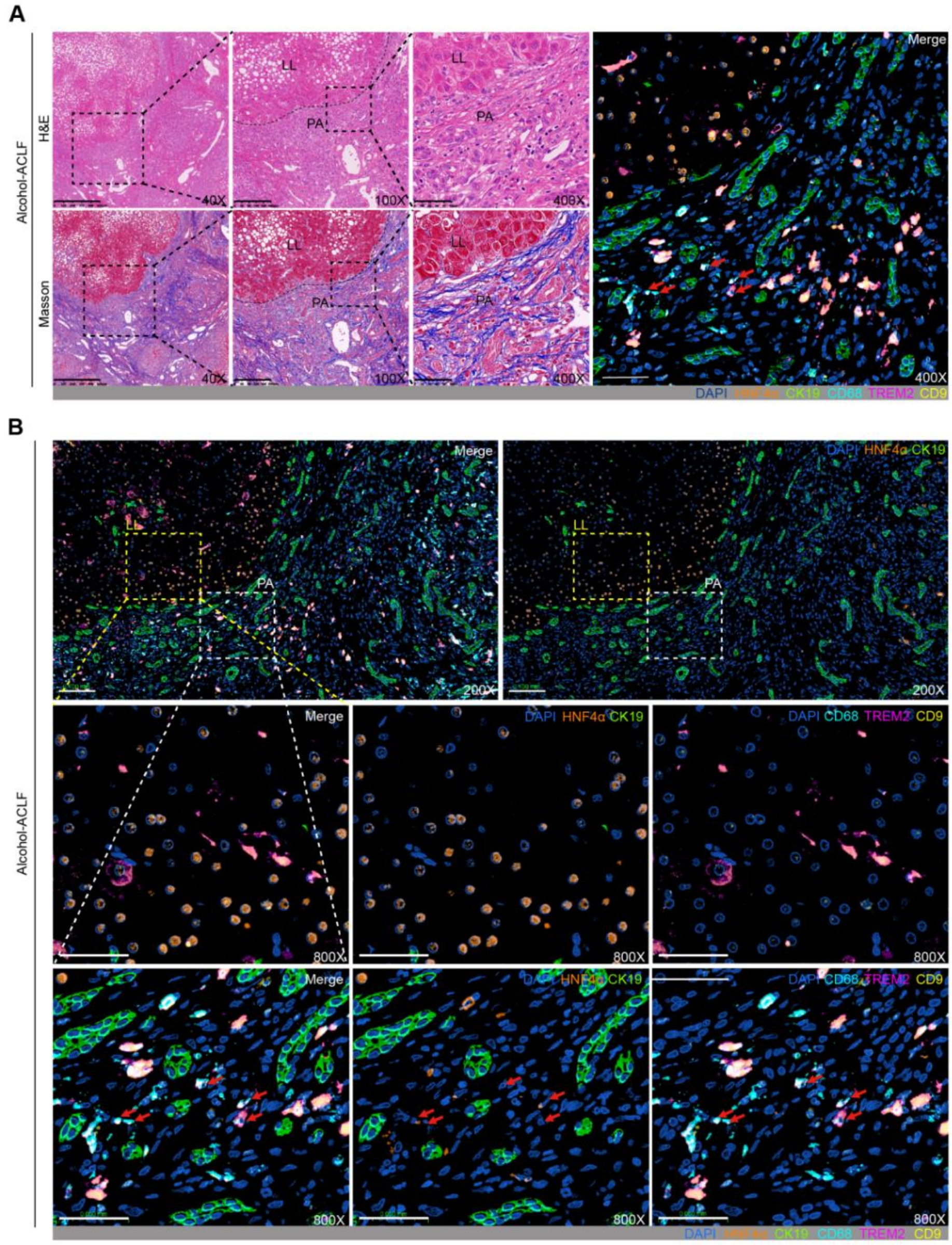

Figure R4. Pathological characteristics and SAMs infiltration sites in alcohol-related ACLF patients . Related to Figure S5 (New data).

(A) Representative H&E staining, Masson trichrome and mIHC images of the patient's liver. Scale bars, 0.625 mm at 40x, 0.2 mm at 100x, and 0.05 mm at 400x. (B) Representative mIHC images of the ACLF patients. Scale bars, 0.1 mm at 200x and 0.05 mm at 800x. Hepatocyte marker (HNF4α) and cholangiocyte marker (CK19) were used to identify liver structures including liver lobular (LL) and portal areas (PA); CD68, TREM2, and CD9 were used to identify SAMs (Red arrows).

2. Similar tissue localization studies should be performed in the animal models, which are distinct from HBV-driven ACLF both etiologically and phenotypically. Greater detail on SAM localization within the tissue and their effects on remaining healthy tissue is needed.

Response: We thank the reviewer for this comment. Based on the reviewer's suggestions, hepatocyte marker (HNF4α) and the cholangiocyte marker (CK19) have been included in mIHC images of ACLF mouse livers **(Fig. R5)**.

In ACLF model mice liver, SAMs are enriched in the portal areas **(Fig. R5)**. In ACLF patients, SAMs were also primarily enriched in the portal areas surrounding collagen-positive scar regions rather than confined to necrotic regions, independent of etiology **(Fig. R1-R4)**. Thus, the spatial distribution of SAMs is phenotypically consistent between ACLF mice and patients and is independent of disease etiology. This ACLF mouse model partially mimics the pathological and immunological features of ACLF patients, particularly those complicated by bacterial infection, which occurs in up to two-thirds of cases[12]. We have now included this result in the revised manuscript **(page 5, lines 172-182；New data Fig. S8)**.

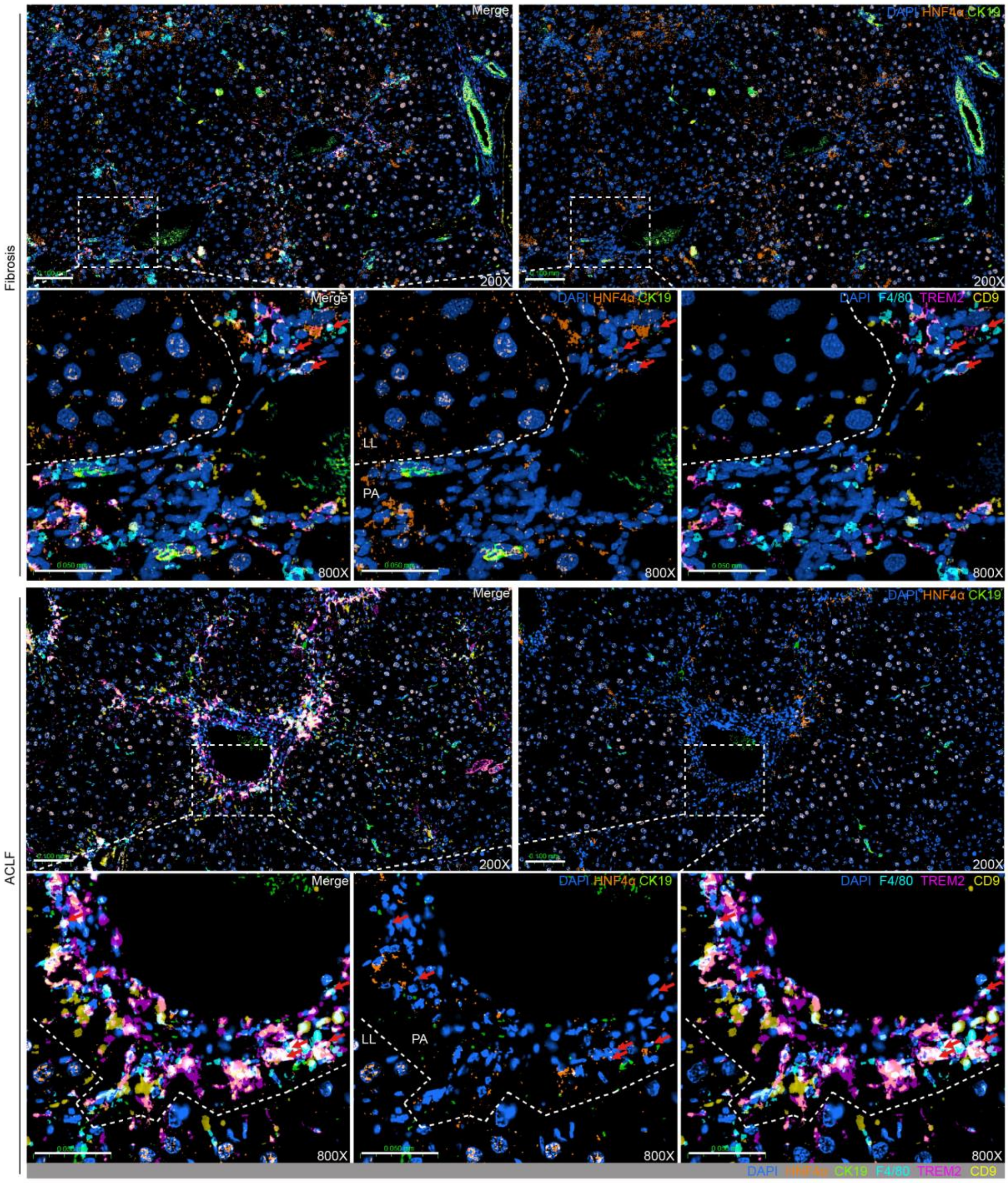

Figure R5. SAMs infiltration sites in fibrosis and ACLF mice. Related to Figure S8 (New data).

Representative mIHC images of the mouse livers. Hepatocyte marker (HNF4α) and cholangiocyte marker (CK19) were used to identify liver structures including liver lobular (LL) and portal areas (PA), while F4/80, TREM2, and CD9 were used to identify SAMs (Red arrows). Scale bars, 0.1 mm at 200x and 0.05 mm at 800x.

3. ACLF Cohort:
Clarification is required regarding the origin and etiology of the ACLF cohort for liver and PBMC analysis. Do these livers exhibit submassive necrosis, suggesting HBV-driven ACLF? Providing this context is crucial for interpretation.

Response: We thank the reviewer for this comment. Based on the reviewer's suggestions, we have supplemented detailed background information regarding the origin and etiology of all ACLF patient cohorts in the supplementary file entitled "**Table 1**. Information of ACLF cohort."

We would like to clarify that while submassive necrosis is not the primary focus of our study, we have summarized the relevant information as follows:

In the previous version of the manuscript, our study included both internal and external cohorts, and all internal cohorts consisted of HBV-related patients, recruited from Shanghai Renji Hospital. Only patients in the liver mIHC cohort underwent histological staining, through which we confirmed the presence of submassive necrosis (defined as necrosis involving 15-90% of the entire liver on explant[1]). For the other internal cohorts and all external datasets, regardless of HBV infection status, the presence of submassive necrosis was not histologically verified. A prospective cohort study of 174 HBV-related patients suggested that submassive necrosis could distinguish ACLF from cirrhosis. Nevertheless, the absence of non-HBV patients limited the evaluation of SMHN in ACLF of other etiologies.

Our internal cohorts included four groups: ①the flow cytometry cohort of PBMCs (ACLF vs. LC = 27 vs. 34), ②the liver mIHC cohort (ACLF vs. LC = 12 vs. 12), ③the liver RNA-seq cohort (ACLF vs. LC vs. HC = 18 vs. 17 vs. 18), and ④the liver mononuclear cell (LMC) mRNA cohort (ACLF vs. LC = 10 vs. 10). Detailed patient information for these internal cohorts is provided in Supplementary **Tables S1-S4**.

4. Animal Model Results:
The authors employed a CCl_4 chronic + CCl_4 acute + Klebsiella model. How do the findings on EP300 expression and SAM infiltration align with human observations of submassive necrosis in HBV? While CCl_4 induces necrosis, Klebsiella elicits an inflammatory response. Understanding EP300 localization and the proposed mechanism of its action is essential.

Response: We thank the reviewer for this comment. The infiltration sites of SAMs were consistent between ACLF patients and ACLF model mice, with both showing enrichment in and around the hepatic portal areas rather than necrosis area **(Fig. R1-R5)**.

EP300 localization: We had previously presented liver multiplex immunohistochemistry (mIHC) staining showing that EP300 showed regions of colocalization with SAMs in both ACLF patients (**Fig. R6A, Related to Figure 6C**) and ACLF mice (**Fig. R6B, Related to Figure 2H**).

EP300 proposed mechanism: SCENIC analysis identified EP300 as the top upstream regulator of these cells **(Fig. R6C).** *EP300* expression was elevated in SAMs from ACLF patients and mice compared with LC or fibrosis, as revealed by single-cell transcriptomic analyses **(Fig. R6D)**. *EP300* expression was also elevated in human ACLF livers compared with LC livers **(Fig. R6E)**, and higher expression correlated with mortality **(Fig. R6F).** mIHC Staining showing that EP300 showed regions of colocalization with SAMs **(Fig. R6A, R6B).** These findings collectively

supporting a potential regulatory role of EP300 in SAMs, and we ultimately confirmed that EP300 regulates the antibacterial immune function of SAMs and attenuates the progression of ACLF. We have now included this result in the revised manuscript **(page 6, lines 197-200；New data Fig. 2G)**.

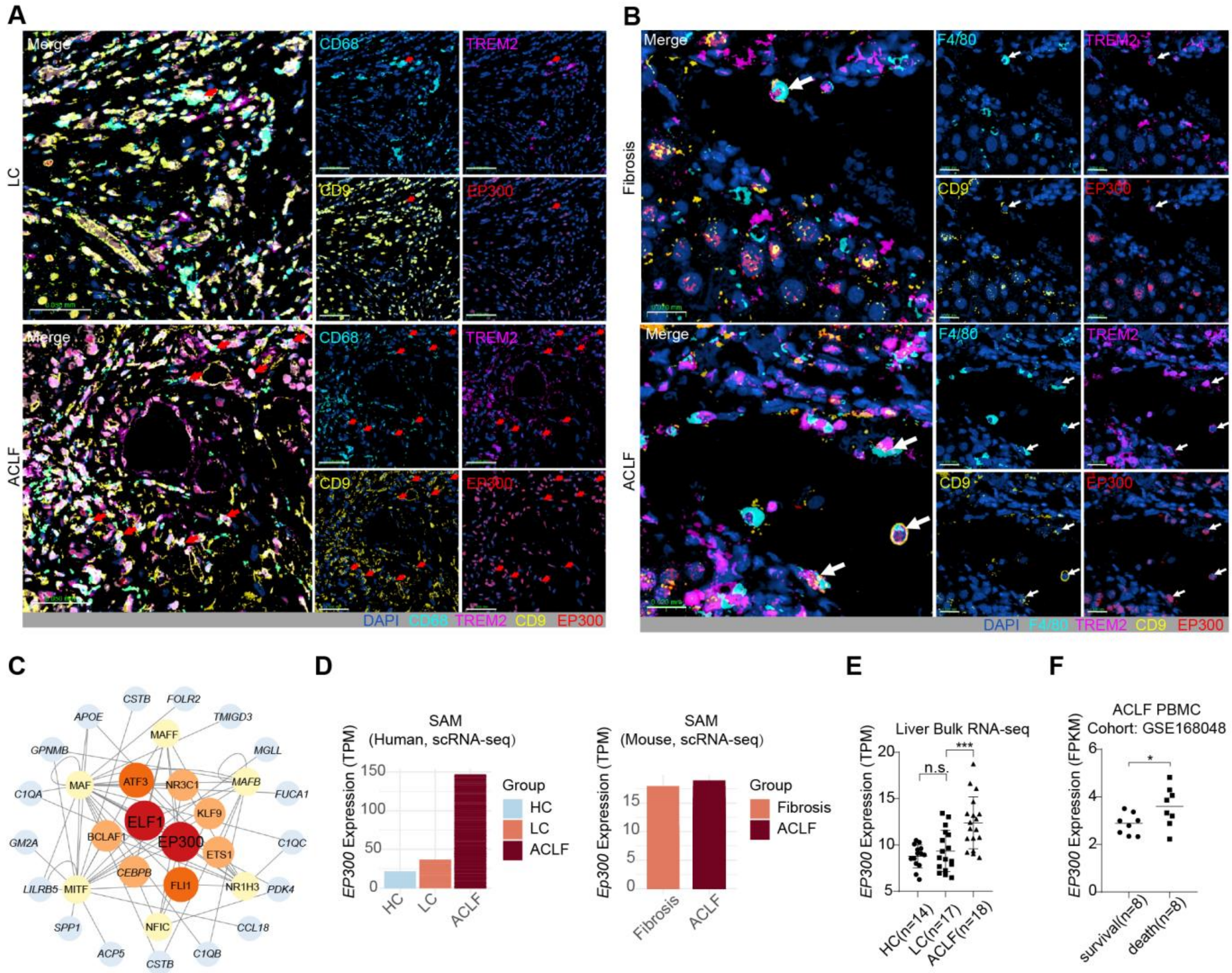

Figure R6. EP300 is a regulator of SAMs in ACLF. Related to Figure 2F-2J and 6C (R6D, New data).

(A) Representative mIHC images of SAMs ($CD68^+Trem2^+Cd9^+$, red arrows) in patients livers and co-localization of SAMs with the Ep300. Scale bars, 0.05 mm. (B) Representative mIHC images of SAMs ($F4/80^+Trem2^+Cd9^+$, white arrows) in mice livers and co-localization of SAMs with the Ep300. Scale bars, 0.02 mm. (C) Prediction of key regulators for SAMs (outer blue circles represent genes regulated by the six light yellow circles and that continuously increasing during SAM differentiation; six light yellow circles denote the intersection TFs; inner circles display the prediction results, where darker colors indicate more promising results). (D) *EP300* expression in SAMs revealed by single-cell transcriptomic analyses of human and mouse livers. Y axis represents relative expression level (TPM). (E) Expression level of *EP300* at HC and different stages of HBV-related liver disease from our liver bulk RNA-seq data. Y axis represents relative expression level (TPM). (F) Expression level of EP300 from PBMC bulk RNA-seq data in ACLF patients with survival and death outcomes. Y axis represents relative expression level (FPKM). Bars represent mean ± SD; **P < 0.01, *P < 0.05, n.s., no significance.

5. Figure 2: The claim that EP300 is expressed in $Trem2^+Cd9^+$cells conflicts with the images, which suggest EP300 colocalization with Cd9 but not with F4/80 or Trem2. This discrepancy

needs to be addressed. Additionally, arrows pointing to cells within vessels likely represent artifacts and should be clarified or removed. (See also Figure 3 for SAMs: F4/80□Trem2□Cd9□.)

Response: We thank the reviewer for spotting these issues. We have optimized the antibody and staining conditions to reduce background noise. EP300 showed regions of colocalization with SAMs triple positive for F4/80, Trem2, and Cd9 **(Fig. R7)**.

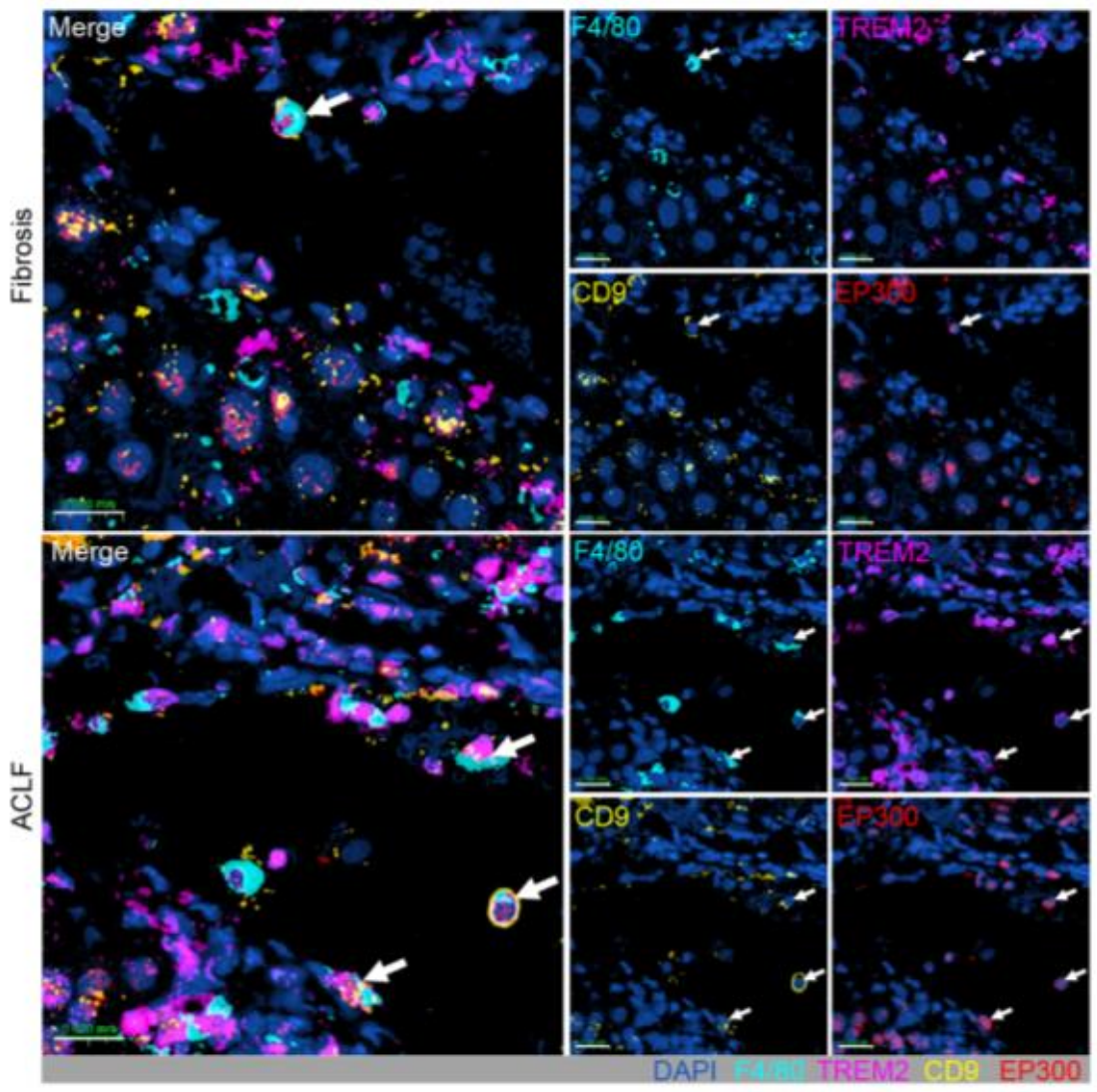

Figure R7. EP300 showed regions of colocalization with SAMs. Related to Figure 2H.

6. If EP300 is predominantly located in Cd9$^+$cells, what is the proposed mechanism by which EP300 inhibition reduces SAMs? Is this a direct effect on their phenotype, or is it an indirect consequence of reduced tissue injury and subsequent recruitment?

Mechanism of EP300 in Injury：The mechanism by which EP300 modulates injury requires further explanation. While inhibition of EP300 reduces injury and SAMs, it remains unclear whether this occurs through direct effects on hepatocytes or alternative pathways.

Response: We thank the reviewer for raising this important point. EP300 showed regions of colocalization with SAMs triple positive for F4/80, Trem2, and Cd9 rather than being colocalized with Cd9$^+$cells **(Fig. R8)**.

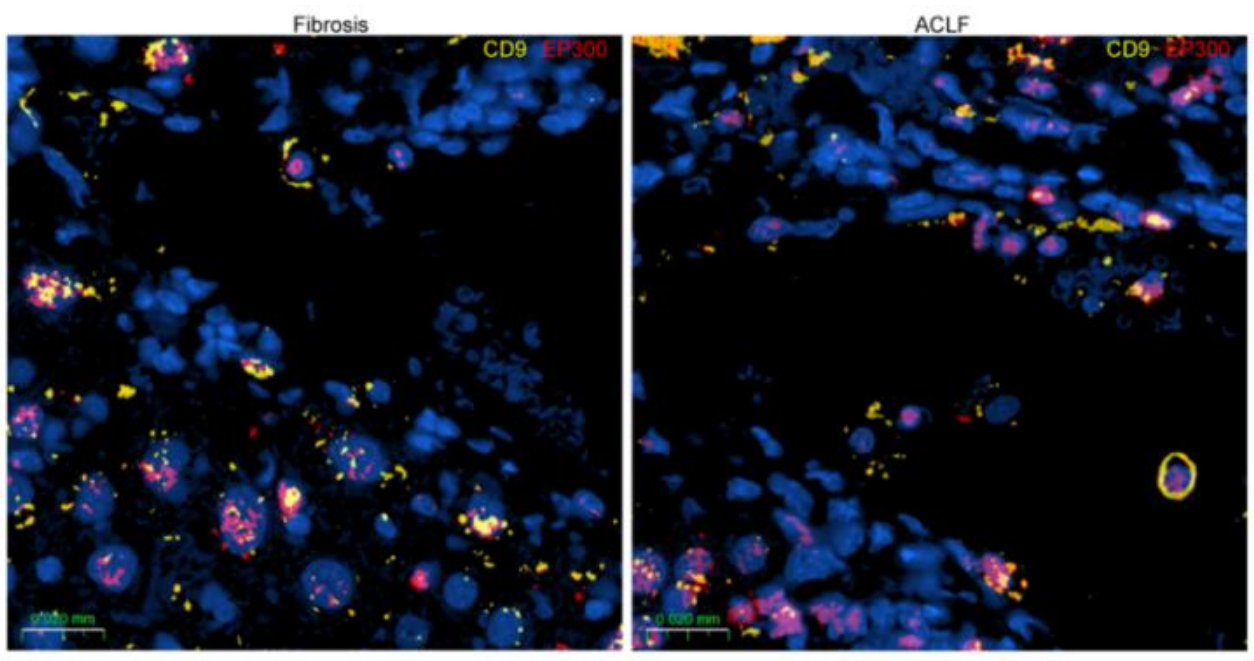

Figure R8. Representative mIHC images of Cd9 and Ep300.

Proposed mechanism of EP300 in SAMs: EP300 inhibition reprograms SAMs via ACOD1, thereby enhancing their antibacterial immune function, reducing bacterial load in ACLF patients, and alleviating tissue injury to alleviate disease progression **(Fig. R9)**. Mechanistically, EP300 represses Acod1 expression by decreasing p300 occupancy and reducing surrounding H3K27

acetylation at the Acod1 promoter region **(Fig. R10)**. Additionally, EP300 inhibitor CCS1477 slightly reduced SAM numbers but primarily enhanced their antibacterial immune function.

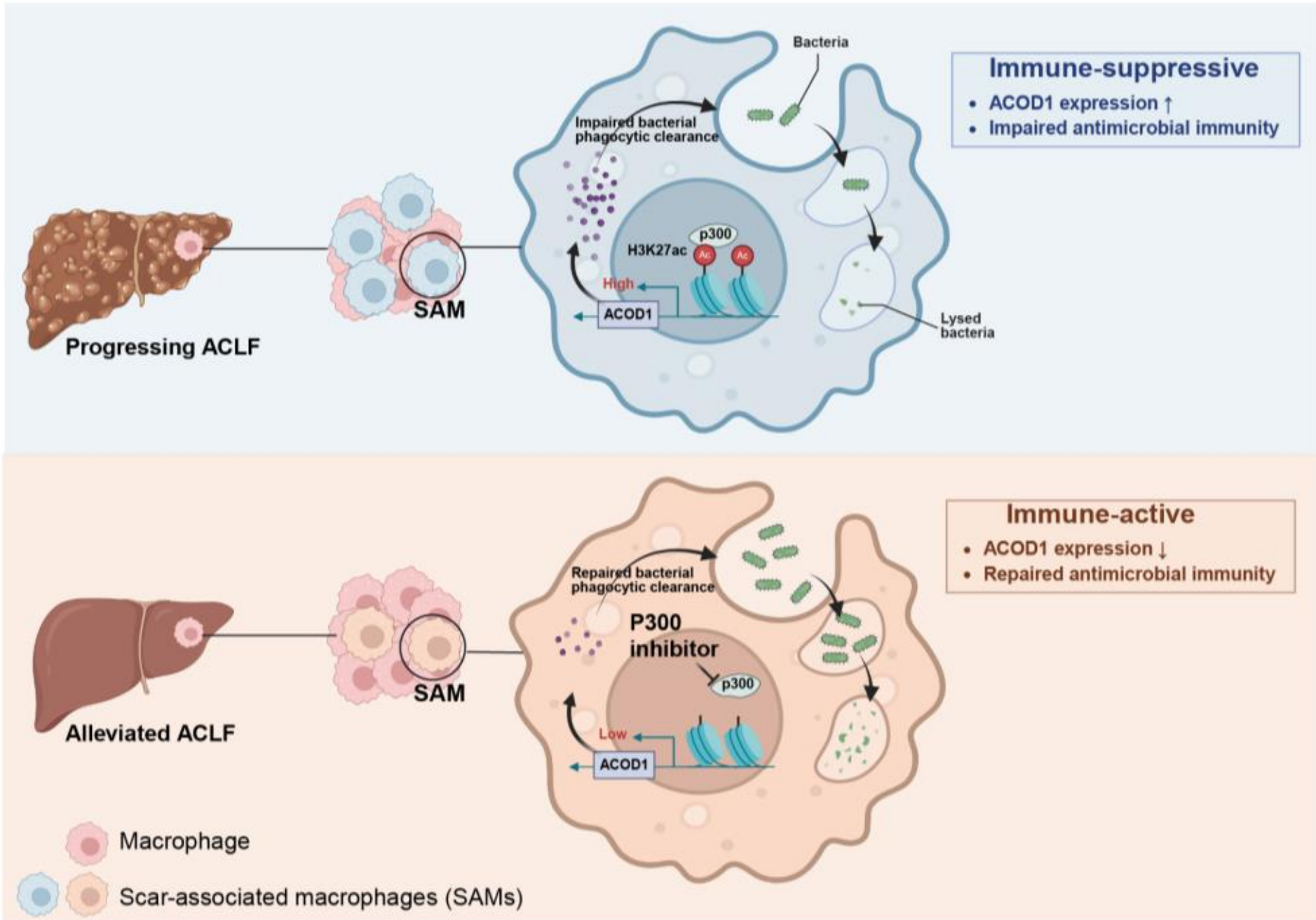

Figure R9. Schematic summary of EP300 inhibition alleviating ACLF by modulating SAMs via ACOD1. Related to Figure S14.

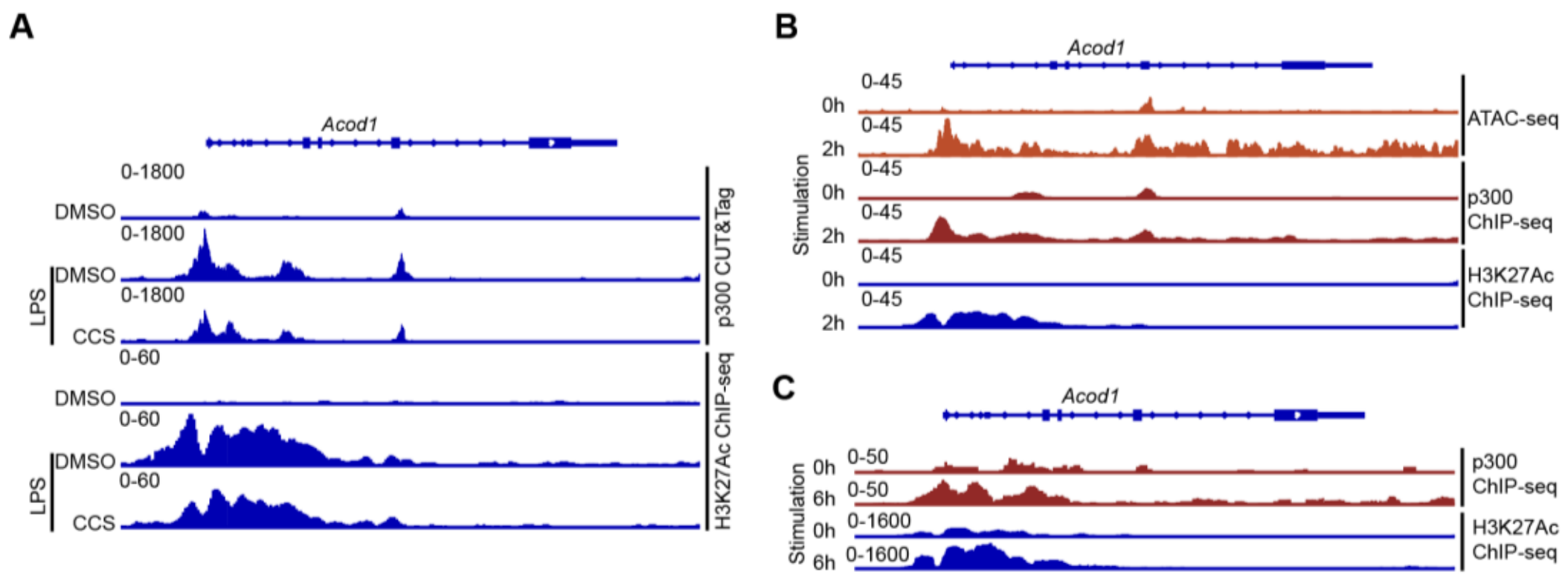

Figure R10. CCS1477 transcriptionally represses Acod1 expression. Related to Figure 5E-5G.

(A) Genome browser views of p300 at *Acod1* before and after LPS stimulation, with and without CCS1477 treatment in RAW264.7 cells. (B, C) Genome browser views of chromatin accessibilities, p300 and H3K27ac at *Acod1* after stimulation at 0 h, 2 h and 6 h in mice BMDMs. CCS, CCS1477.

EP300 inhibition alleviates ACLF by directly enhancing SAM phagocytic function: ①Not mediated by SAM numbers

We apologize for the earlier misinterpretation that part of the therapeutic effect of EP300 inhibition was due to a reduction in SAM numbers. Upon further investigation, we confirmed that SAMs have protective antibacterial activity in ACLF, which becomes impaired during disease progression, resulting in higher pathogen burden and aggravated injury. The EP300 inhibitor CCS1477 alleviated ACLF primarily by enhancing the antibacterial immunity of SAMs rather

than reducing their number. The reduction in SAM numbers represents a minor secondary effect of CCS1477 and do not affect its efficacy.

②Direct enhancement of SAM antibacterial function

To validate this, we performed adoptive transfer of in vitro bone marrow-derived SAMs into ACLF mice **(Fig. R11A, R11B)**. ACLF mice receiving untreated SAMs (WT-SAM-CON) showed prolonged survival and reduced plasma bacterial loads compared with the PBS group **(Fig. R11C, R11D)**, indicating that SAMs exert protective antibacterial immune effects in ACLF. Furthermore, ACLF mice receiving CCS1477-treated SAMs (WT-SAM-CCS) exhibited further improvements in survival and marked reductions in plasma bacterial burden compared with the WT-SAM-CON group **(Fig. R11C, R11D)**, suggesting that EP300 inhibition CCS1477 enhances the antibacterial immunity of SAMs to alleviate ACLF progression. We have now included this result in the revised manuscript **(page 10, lines 319-335；New data Fig. 5H-5J, S12G)**.

Collectively, these results demonstrate that CCS1477 alleviates ACLF mainly by directly enhancing SAM antibacterial function, rather than by reducing SAM numbers or acting through other pathways.

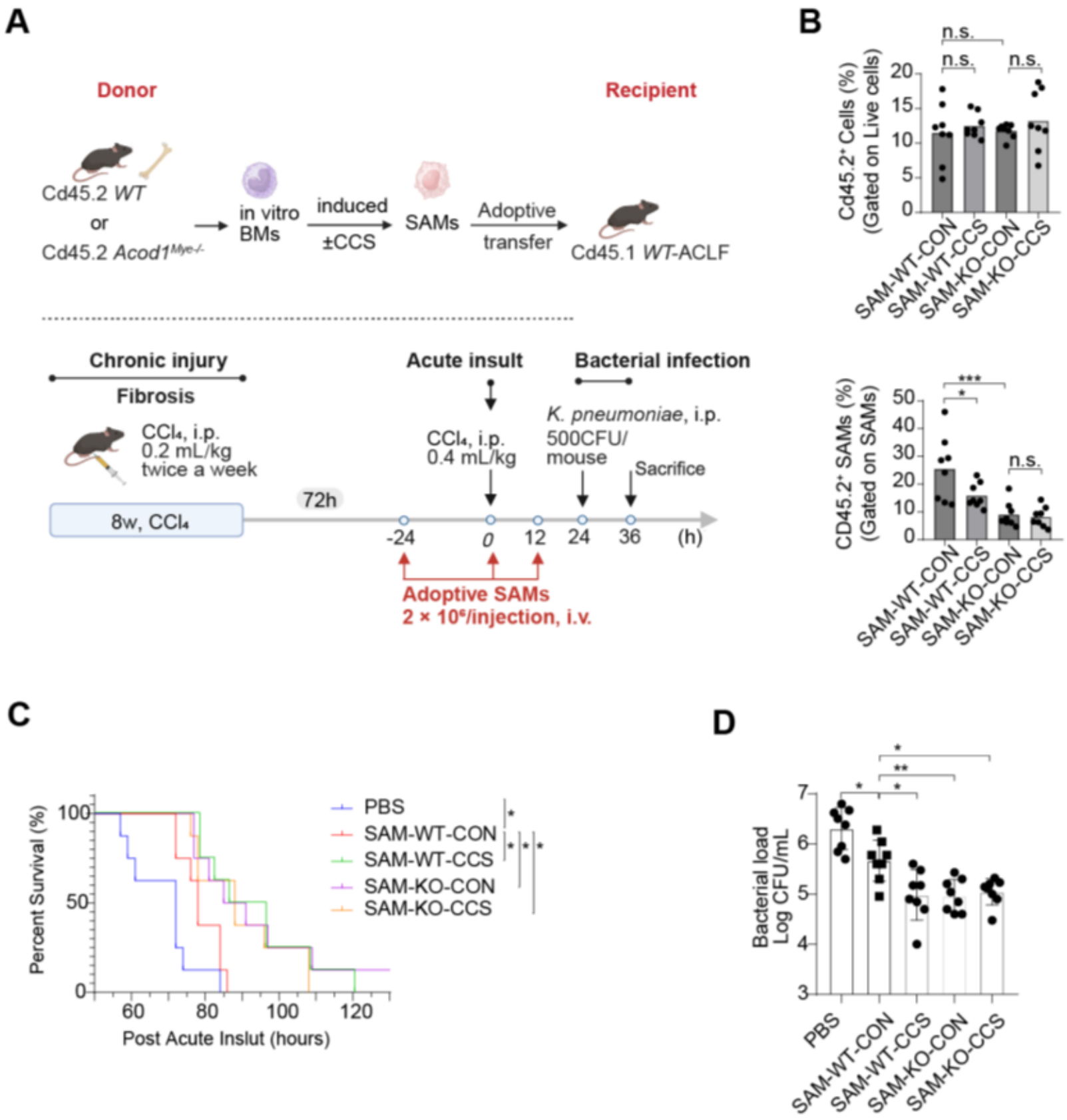

Figure R11. Induction of SAMs in vitro and adoptive transfer of SAMs. Related to Figure 4H-4J and S12G (New data).

(A) Schematic of adoptive transfer of in vitro–induced SAMs from CD45.2 mice into CD45.1 ACLF mice. (B) Flow cytometry analysis showing the proportion of $CD45.2^+$ cells within the live cell population and SAMs ($Trem2^+Cd9^+$) within the $CD11b^+F4/80^+$ gated population in CD45.1 ACLF recipient mice after adoptive transfer of SAMs from different groups (n = 8). (C) Survival analysis of ACLF model mice after adoptive transfer of SAMs from different groups or PBS. (D) Blood bacterial loads in ACLF model mice after adoptive transfer of SAMs from different groups or PBS. Bars represent mean ± SD; $^{**}P < 0.01$, *n.s.*, no significance. CCS, CCS1477; BMs, bone marrow cells; SAM-WT-CON, in vitro-induced SAMs from WT mouse bone marrow treated

with DMSO; SAM-WT-CCS, in vitro-induced SAMs from WT mouse bone marrow treated with CCS1477; SAM-KO-CON, in vitro-induced SAMs from *Acod1*$^{Mye-/-}$ mouse bone marrow treated with DMSO; SAM-KO-CCS, in vitro-induced SAMs from *Acod1*$^{Mye-/-}$ mouse bone marrow treated with CCS1477.

7. Terminology：
Avoid the term "necroptotic apoptosis." Specify whether necroptosis or apoptosis is being referred to.
Response: We agree with the reviewer and have accordingly revised "necroptotic apoptosis" to "necroptosis" throughout the manuscript.

8. Acod1 Knockout：
Acod1 knockout animals showed prolonged survival in ACLF models, with reduced SAMs, less injury, and lower bacterial loads. Since Acod1 regulates phagocytosis, these effects are hypothesized to result from diminished phagocytic activity. However, if this hypothesis is correct, Acod1 knockout might not affect the CCl_4 model. This raises Comments about its relevance for HBV-ACLF patients without infection.
Response: We thank the reviewer for this insightful suggestion. As a key regulator during inflammation and infection, Acod1 exerts context-dependent effects, varying with the specific disease, disease stage and cell types[4-6]. In the chronic CCl_4 phase, *Acod1* knockout in myeloid cells exerted no protective benefit but rather promoted fibrosis **(Fig. R12)**. Therefore, Acod1 regulates SAMs predominantly during the acute phase characterized by bacterial infection. We confirmed that EP300 inhibition reprograms SAMs via ACOD1, enhances their antibacterial immune function, reduces bacterial load, and attenuates ACLF progression.
Based on our current data, these conclusions appear to be restricted to infection-associated ACLF. Whether they also apply to non-infectious ACLF requires further experimental investigation, which we will discuss as a limitation. This direction deserves further exploration in future studies.
Our findings are applicable to infection-exacerbated ACLF patients:
Our study focused on ACLF patients with bacterial infection, which occurs in up to two-thirds of cases and represents a major prognostic determinant[12]. Using the ACLF mouse model that recapitulates the key clinical features of bacterial infection-exacerbated ACLF without being limited to a specific disease etiology[13-15]. We have now included this result in the revised manuscript **(page 9, lines 287-295；New data Fig. S11)**.

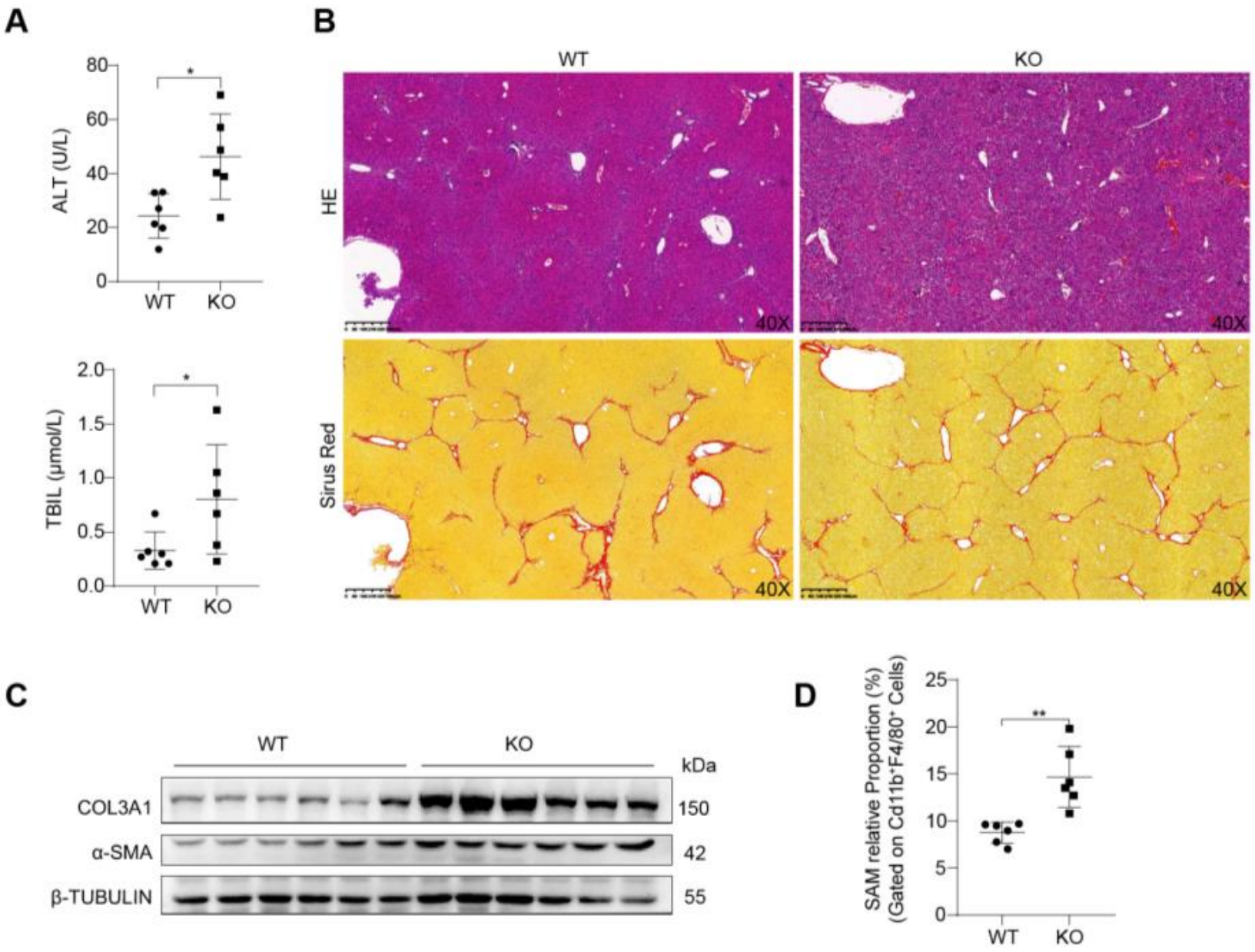

Figure R12 *Acod1$^{Mye-/-}$* mice exhibit exacerbated liver fibrosis and increased numbers of SAMs. Related to Figure S11 (New data).

(A) Serum ALT and TBIL levels in the fibrosis mouse model of WT and *Acod1$^{Mye-/-}$* mice. (B) Representative images of H&E and Sirius Red staining in the liver. (C) Western blot analysis showing protein levels of COL3A1 and α-SMA in the liver. (D) Flow cytometry analysis in the proportion of SAMs (Trem2$^+$Cd9$^+$) within the Cd11b$^+$F4/80$^+$ gated population in liver (n = 6).

9. EP300 Inhibition and Acod1: EP300 inhibition via CCS1477 transcriptionally suppresses Acod1, but this is perplexing given that EP300 does not appear to be expressed in true SAMs. Clarification of this mechanism is necessary.

Response: We thank the reviewer for this insightful comment. We optimized the staining conditions and confirmed that EP300 showed regions of colocalization with SAMs in both ACLF patients and mouse livers. **(Fig. R13A)**. And we have supplemented single-cell transcriptomic data from both human and mouse demonstrate that Ep300 is expressed in SAMs and shows an increasing trend with disease progression **(Fig. R13B)**. We confirmed that EP300 inhibition reprograms SAMs via ACOD1, thereby enhancing their antibacterial immune function, reducing bacterial load in ACLF patients, and alleviating tissue injury to alleviate disease progression.

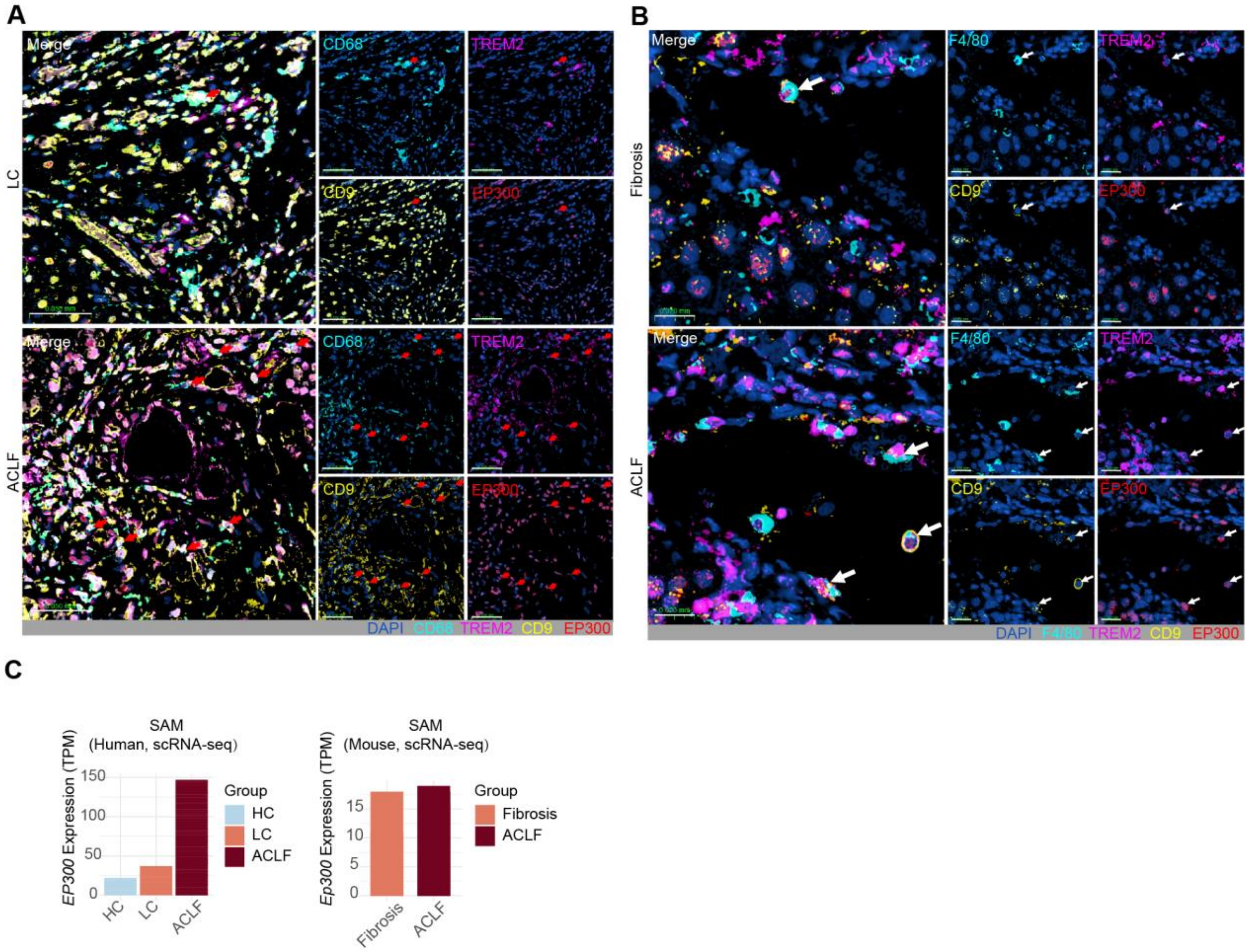

Figure R13. EP300 was expressed in SAMs and showed regions of colocalization with SAMs. R13A Related to Figure 6C, R13B Related to Figure 2H, R13C Related to Figure 2G (New data).

(A) Representative mIHC images of SAMs ($CD68^{+}Trem2^{+}Cd9^{+}$, red arrows) in patients livers and co-localization of SAMs with the Ep300. Scale bars, 0.05 mm. (B) Representative mIHC images of SAMs ($F4/80^{+}Trem2^{+}Cd9^{+}$, white arrows) in mice livers and co-localization of SAMs with the Ep300. Scale bars, 0.02 mm. (C) *EP300* expression in SAMs revealed by single-cell transcriptomic analyses of human and mouse livers. Y axis represents relative expression level (TPM).

10.Acod1$^{Mye-/-}$ Mouse Model:

The use of Acod1$^{Mye-/-}$ mice complicates interpretation as the knockout impacts the entire model, including the chronic CCl_4 phase. Analyzing the effects in both the chronic phase and the combined CCl_4 chronic + acute phase is critical for clearer insights.

Response: We thank the reviewer for this insightful comment. Following the reviewer's suggestion, we have additionally evaluated the impact of *Acod1* knockout in the chronic CCl_4 phase. The results showed that myeloid-specific *Acod1* knockout had no protective effect during the CCl_4-induced chronic liver fibrosis stage.

In CCl_4-induced chronic liver fibrosis stage, *Acod1*$^{Mye-/-}$ mice exhibited higher serum ALT and TBIL levels than WT controls **(Fig. R14A)**, more extensive fibrosis on Sirius Red staining **(Fig. R14B)**, and increased hepatic α-SMA and COL3A1 expression **(Fig. R14C)**. Flow cytometry showed a greater proportion of liver SAMs **(Fig. R14D)**. These findings indicate that *Acod1* KO exacerbates chronic CCl_4-induced fibrosis, suggesting that myeloid-specific *Acod1* knockout

exerts its protective effect mainly during the acute phase rather than the chronic stage in ACLF. We have now included this result in the revised manuscript **(page 9, lines 287-295; New data Fig. S11)**.

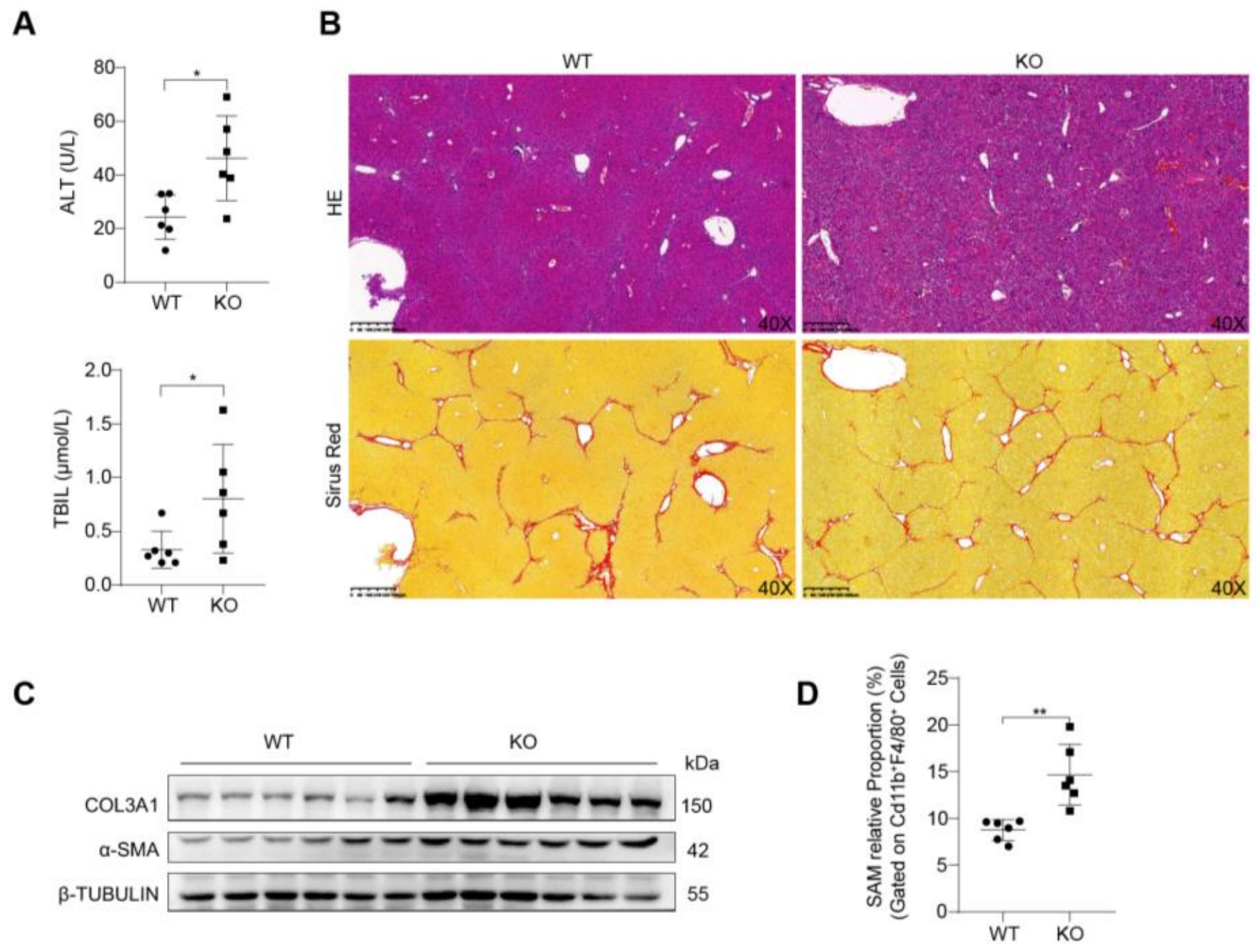

Figure R14 ***Acod1*$^{Mye-/-}$ mice exhibit exacerbated liver fibrosis and increased numbers of SAMs. Related to Figure S11 (New data).**

(B) Serum ALT and TBIL levels in the fibrosis mouse model of WT and *Acod1*$^{Mye-/-}$ mice. (B) Representative images of H&E and Sirius Red staining in the liver. (C) Western blot analysis showing protein levels of COL3A1 and α-SMA in the liver. (D) Flow cytometry analysis in the proportion of SAMs (Trem2$^+$Cd9$^+$) within the Cd11b$^+$F4/80$^+$ gated population in liver (n = 6).

11.Treatment Regimens:

Additional details on the treatment regimens for CCS1477 and other compounds are needed. Were these administered as single or multiple injections? What was the treatment duration, number of injections, and timing relative to the model (e.g., before acute CCl4 or before Klebsiella)?

Response: Thanks for this constructive comment. Detailed information regarding the treatment regimens for CCS1477 and other compounds are provided **(Fig. R15)**.

Mice in the acute exacerbation phase received different pharmacological interventions. EP300 inhibitors, including CCS1477, NEO2734, and GNE207, were administered as a single intraperitoneal injection 2 h prior to *K. pneumoniae* challenge. Imipenem was administered intraperitoneally in three doses: the first injection was given 2 h before *K. pneumoniae* challenge (concurrent with the other compounds), followed by the second and third injections at 4 h and 8 h thereafter, respectively. The treatment duration was limited to the acute phase, and all injections were timed relative to the bacterial challenge rather than the chronic CCl_4 phase. We have now included this result in the revised manuscript **(page 7, lines 229-237; New data Fig. S10A)**.

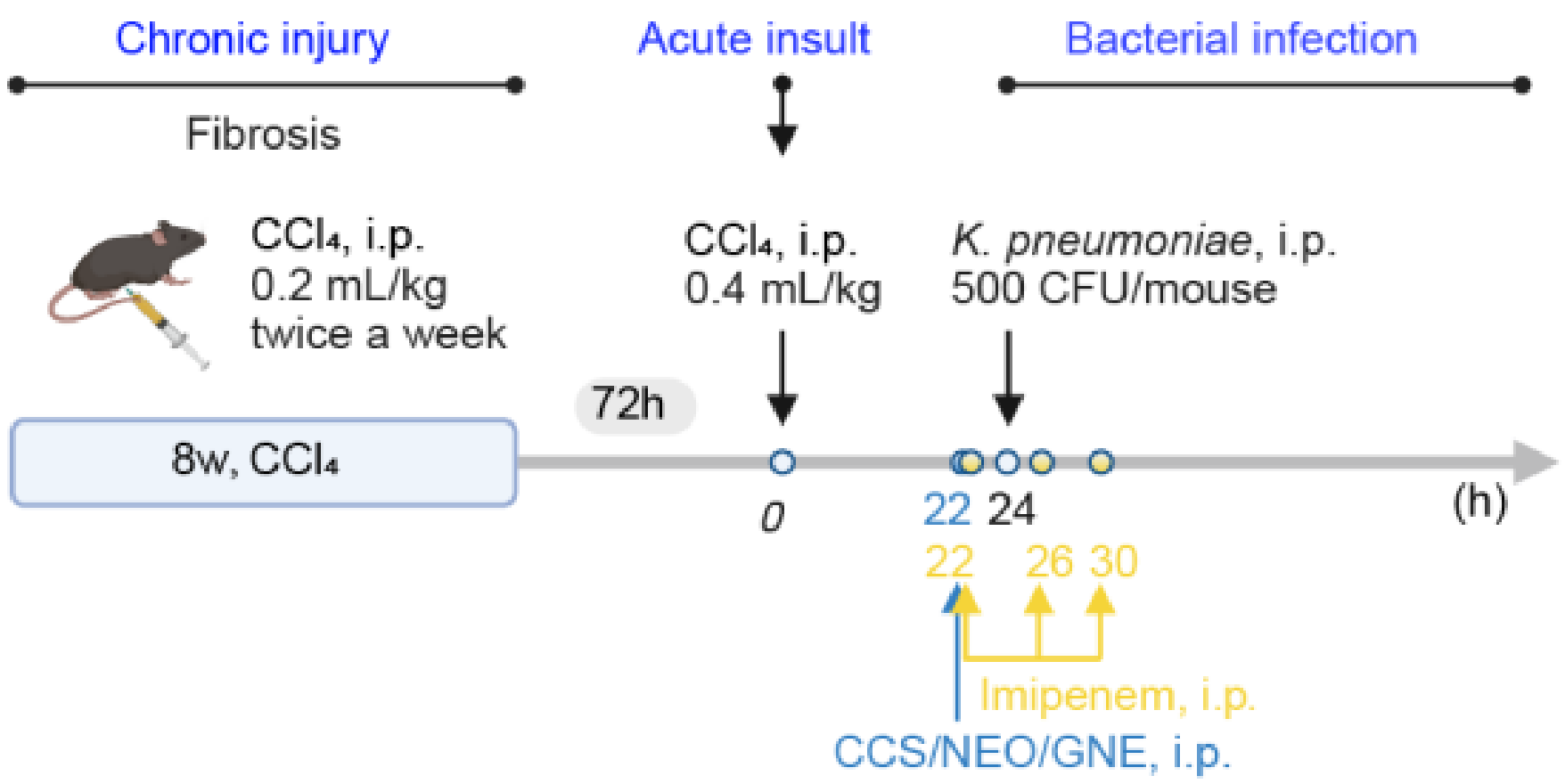

Figure R15. Compounds treatment regimen in ACLF mice. Related to Figure S10A (New data).

Schematic illustration of compound administration.

Reviewer #2 (Remarks to the Author):

In this manuscript, Chen et al. demonstrated that scar-associated macrophages (SAMs) are increased in the livers of acute-on-chronic liver failure (ACLF) both in humans and murine model. They identified EP300 as the most prominent upstream regulator mediating the expression of the transcription factors required for the differentiation SAMs of ACLF. They also demonstrated that ACOD1 expression is induced in ACLF SAMs and its loss mitigates hepatic destruction and extends ACLF mouse survival via enhanced phagocytosis of SAMs. Overall, the data are presented well and the results are potentially interesting, however, there are critical concerns to be addressed.

Response: We sincerely thank the reviewer for the positive evaluation and constructive comments on our work.

Major concerns

1. Although the authors showed the increased number of SAMs in the liver of ACLF and its association with the disease progression, it is unclear how these cells directly contribute to the progression of ACLF.

Response: We thank the reviewer for this insightful comment. In this study, we confirmed that during the progression from liver fibrosis/cirrhosis to ACLF, SAMs were mainly enriched in and around the hepatic portal areas **(Fig. R16)**, where they exert protective antibacterial immune functions. In ACLF, although the number of SAMs increased, their phagocytic capacity was impaired and these SAMs with impaired phagocytic function failed to eliminate pathogens or prevent ACLF progression **(Fig. R17)**. Therefore, strategies aimed at restoring SAM immune function to combat bacterial infections, such as EP300 inhibition or myeloid-specific *Acod1* knockout, can alleviate ACLF in mice and provide promising therapeutic options for ACLF patients **(Fig. R17)**.

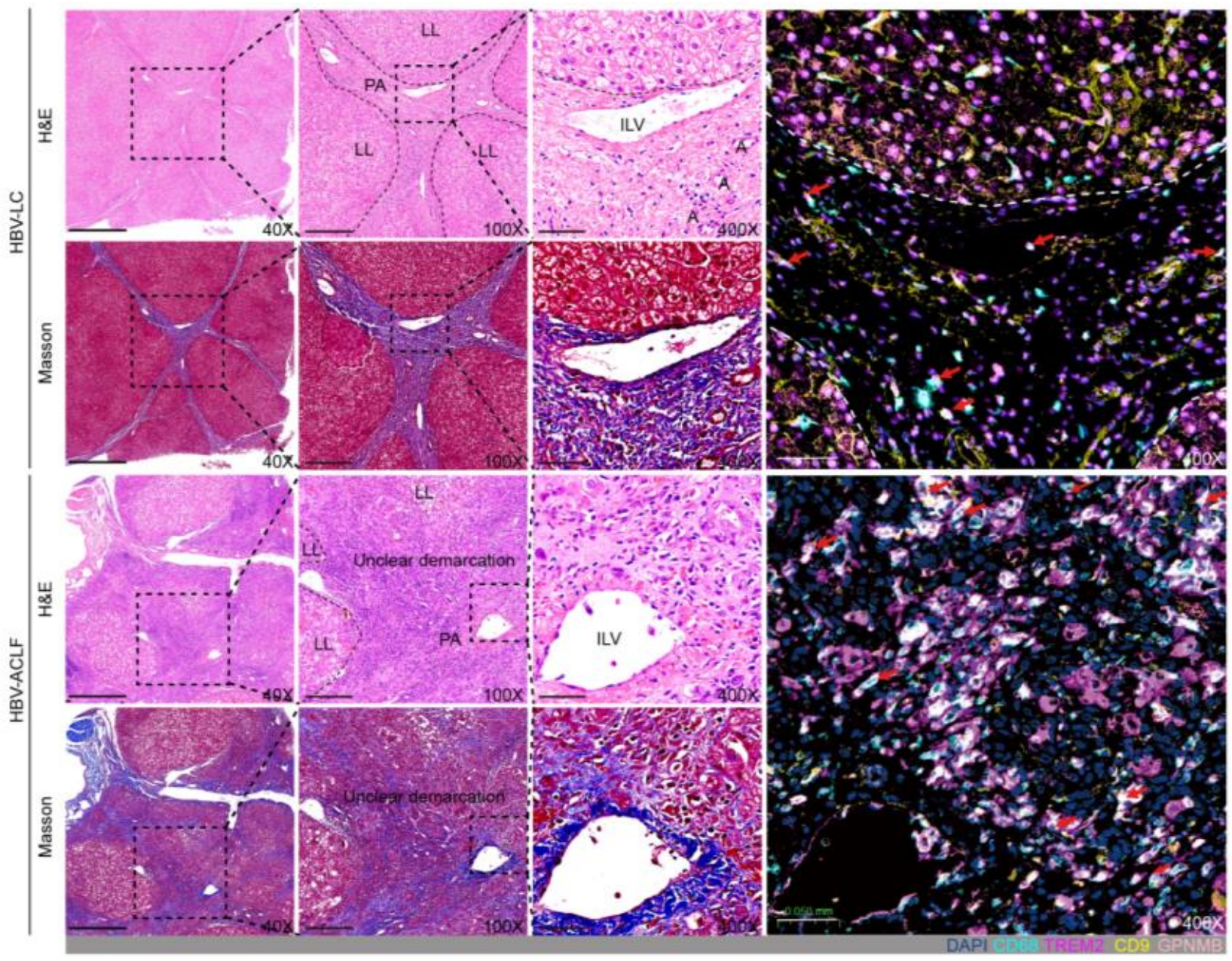

Figure R16. Pathological characteristics and SAMs infiltration sites in HBV-related LC

and HBV-related ACLF patients. Related to Figure S3.

Representative H&E staining, Masson trichrome, and mIHC images of the patient's liver. Compared with LC, ACLF livers show disruption of fibrous septa, poorly demarcated portal and lobular structures, and broader SAM infiltration around the portal areas. Scale bars: 0.625 mm at 40×, 0.2 mm at 100×, and 0.05 mm at 400×. SAMs (red arrows) were identified using CD68, TREM2, CD9, and GPNMB. PA, portal area; LL, liver lobe; ILV, interlobular vein; A, artery.

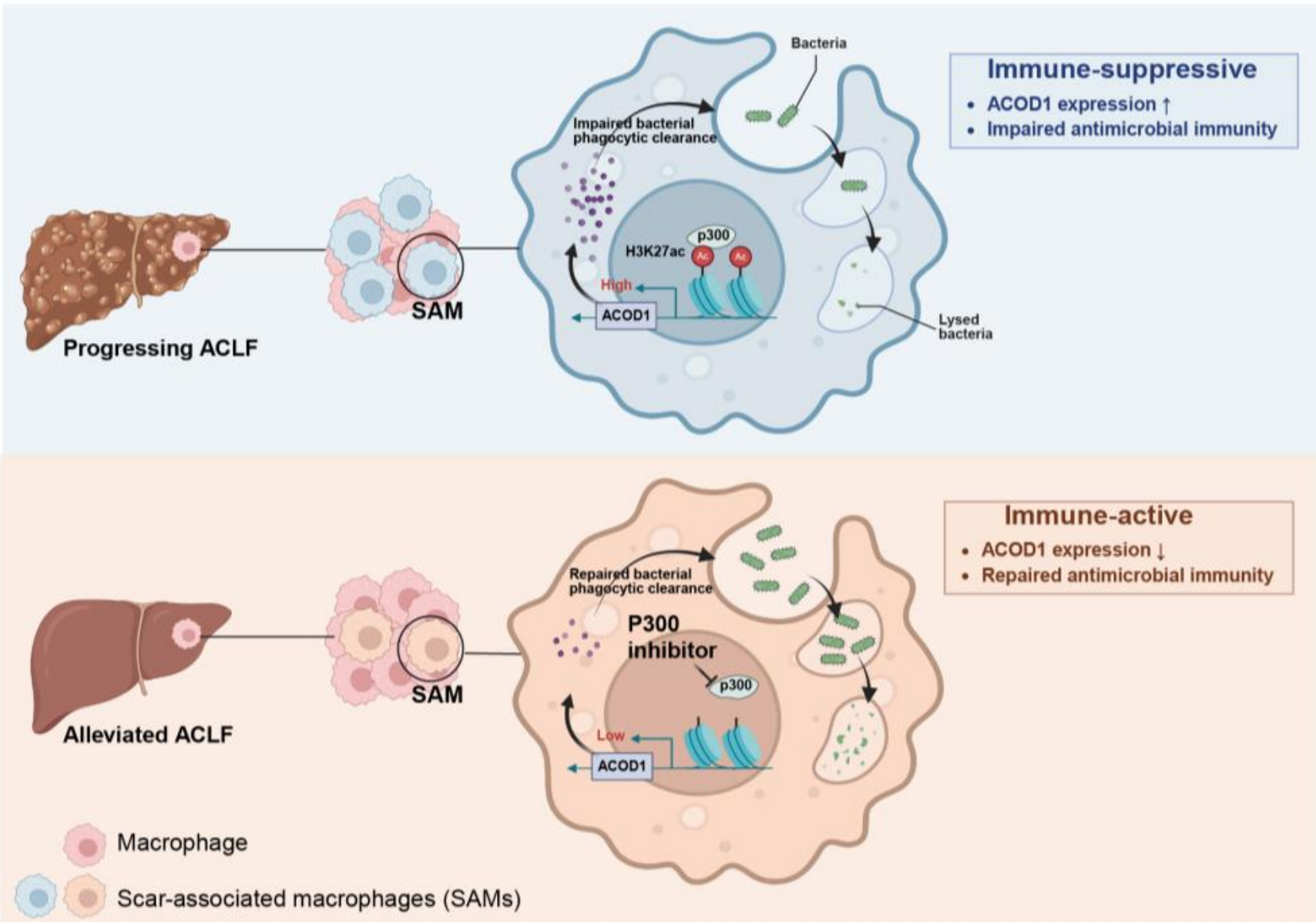

Figure R17. Schematic summary of EP300 inhibition alleviating ACLF by modulating SAMs via ACOD1. Related to Figure S14.

Information on cytokine production and cytotoxicity of SAMs is missing, and in addition to the numbers, it is necessary to show whether their characteristics differ from those of liver fibrosis models.

Response: We thank the reviewer for this suggestion. As recommended, cytokine production by SAMs was additionally evaluated, and the results showed that during the progression from liver fibrosis/cirrhosis to ACLF, cytokine secretion by these cells remained largely unchanged **(Fig. R18)**.

Human and murine single-cell transcriptomic analyses showed that SAMs expressed cytokines such as TNFα, IL1β, IL6, and IL10, with no marked differences between LC/fibrosis and ACLF **(Fig. R18A-R18D)**. Flow cytometry showed that cytokine secretion by SAMs, including TNFα, IL6 and IL10, did not differ between fibrosis and ACLF mice **(Fig. R18E)** (IL1β, TGFβ, and IL-1Ra were not assessed for lack of suitable antibodies). These results suggest that cytokine production by SAMs is not a major determinant of ACLF progression.

We have now included this result in the revised manuscript **(page 10, lines 336-344; New data Fig. S13)**.

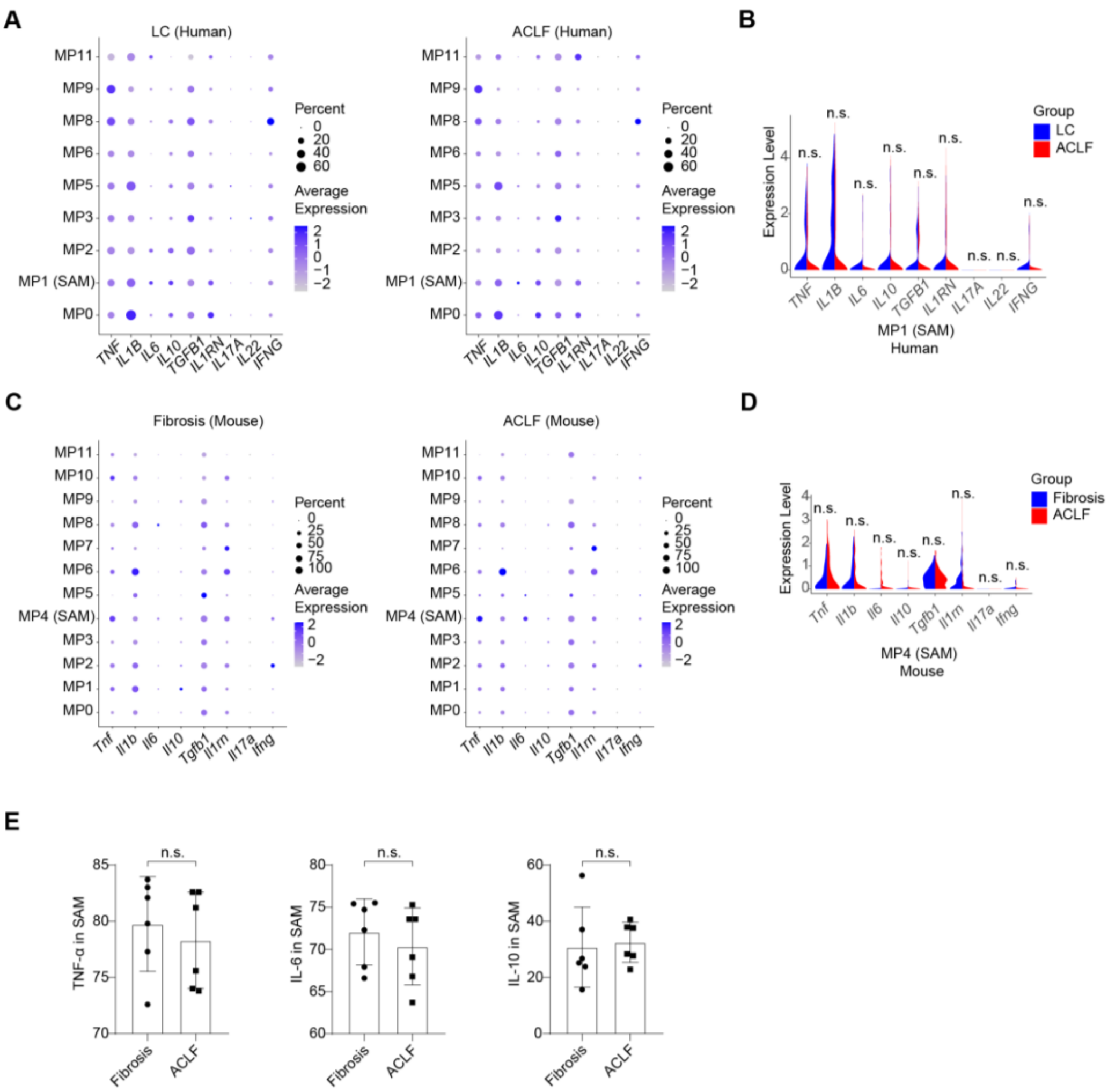

Figure R18. Cytokine secretion by MPs. Related to Figure S13 (New data).
(A) Dot plot showing cytokine expression across MP subsets in the livers of LC and ACLF patients. (B) Violin plot showing cytokine expression in MP1 (SAMs) between LC and ACLF patients. (C) Dot plot showing cytokine expression across MP subsets in the livers of fibrosis and ACLF mouse models. (D) Violin plot showing cytokine expression in MP4 (SAMs) between fibrosis and ACLF mouse models. (E) Flow cytometry analysis showing cytokine secretion in SAMs ($Trem2^{+}Cd9^{+}$) from fibrosis and ACLF mice (n = 6).

Furthermore, the direct involvement of this cell subset in the progression of ACLF must be demonstrated by depleting these cells both in liver fibrosis and ACLF mouse models.

Response: We thank the reviewer for this comment. We regret that selective in vivo ablation of SAMs could not be performed, as current approaches face technical and feasibility limitations. The Trem2-iDTR system is nonspecific and costly, and clodronate liposome depletion caused rapid death of ACLF mice, preventing SAM-specific analysis.

Instead, we performed adoptive transfer of in-vitro bone marrow–derived SAMs into ACLF mice **(Fig. R19A, R19B)**, which allowed more precise control of SAM numbers. Compared with PBS transfer, SAM transfer prolonged survival and lowered plasma bacterial load **(Fig. R19C, R19D),** indicating that in-vitro–induced SAMs possess antibacterial phagocytic activity in ACLF mice. Transfer of SAMs pretreated with the EP300 inhibitor CCS1477 further improved survival

and reduced bacterial load relative to untreated SAMs **(Fig. R19C, R19D)**, indicating that EP300 inhibition enhances SAMs' antibacterial function, thereby attenuating disease progression.

Details of the adoptive transfer experiments: Bone marrow cells from *WT* and *Acod1$^{Mye-/-}$* mice were differentiated into SAMs in vitro, treated with or without the Ep300 inhibitor CCS1477, and then transferred three times into ACLF recipient mice (CD45.1 background), while donor SAMs were CD45.2^{+} **(Fig. R19A)**. The hepatic abundance of transferred CD45.2^{+} cells was similar across groups, but the proportion of SAMs within CD45.2^{+} cells was reduced in mice receiving CCS1477-pretreated WT SAMs **(Fig. R19B)**. This transfer prolonged survival **(Fig. R19C)** and lowered bacterial load **(Fig. R19D)**, indicating that EP300 inhibition alleviates ACLF mainly by enhancing SAM antibacterial function, with the reduced SAM proportion reflecting disease remission. Taken together, these results indicate that SAMs contribute to ACLF primarily through their antibacterial function, and that EP300 inhibition alleviates ACLF injury by directly enhancing the phagocytic capacity of SAMs.

We have now included this result in the revised manuscript **(page 10, lines 319-335；New data Fig. S12)**.

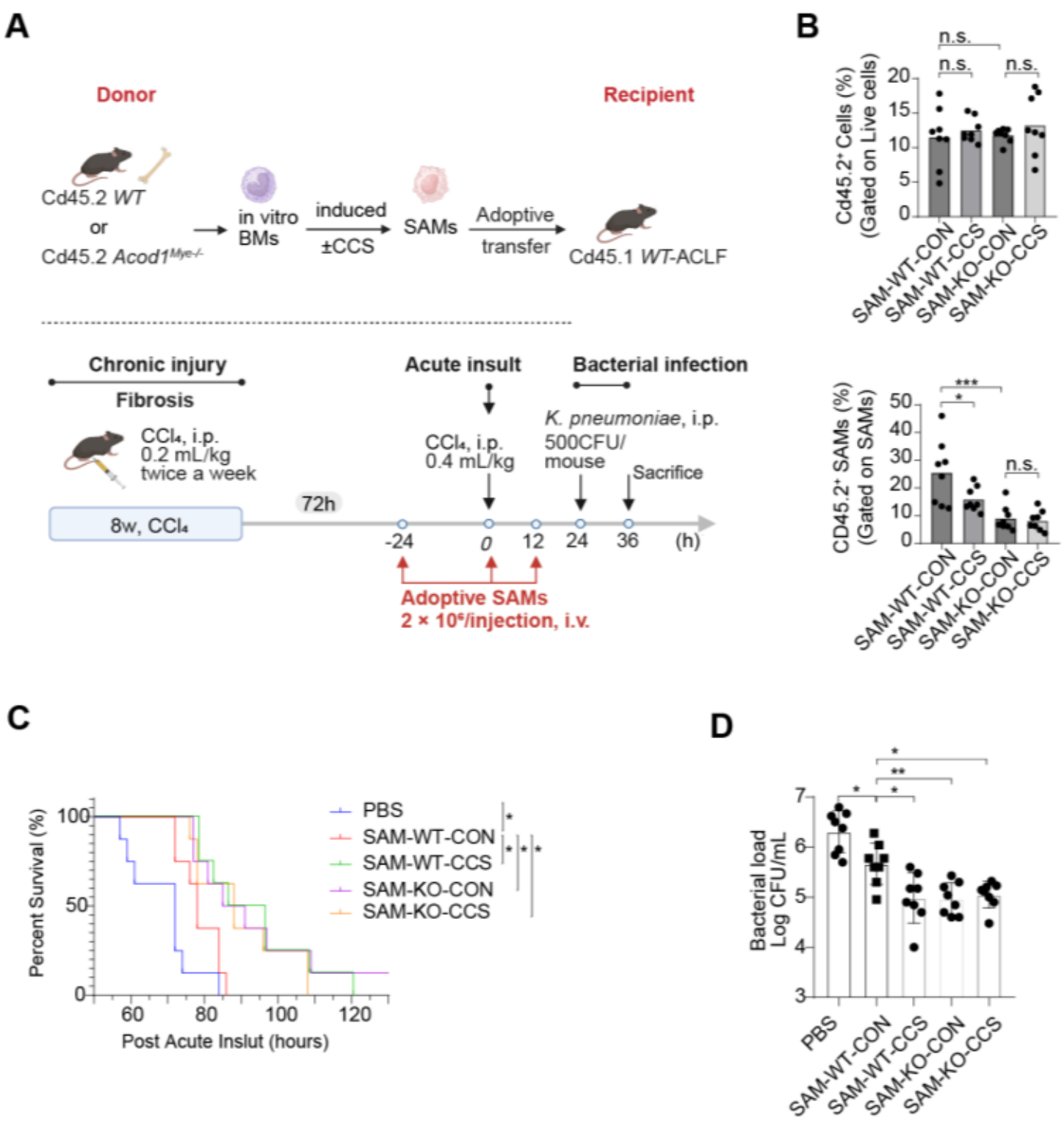

Figure R19. Induction of SAMs in vitro and adoptive transfer of SAMs. Related to Figure 4H-4J and S12G (New data).

(A) Schematic of adoptive transfer of in vitro–induced SAMs from CD45.2 mice into CD45.1 ACLF mice. (B) Flow cytometry analysis showing the proportion of CD45.2^{+} cells within the live cell population and SAMs (Trem2^{+}Cd9^{+}) within the CD11b^{+}F4/80^{+} gated population in CD45.1 ACLF recipient mice after adoptive transfer of SAMs from different groups (n = 8). (C) Survival analysis of ACLF model mice after adoptive transfer of SAMs from different groups or PBS. (D) Blood bacterial loads in ACLF model mice after adoptive transfer of SAMs from different groups

or PBS. Bars represent mean ± SD; $^{**}P < 0.01$, *n.s.*, no significance. CCS, CCS1477; BMs, bone marrow cells; SAM-WT-CON, in vitro-induced SAMs from WT mouse bone marrow treated with DMSO; SAM-WT-CCS, in vitro-induced SAMs from WT mouse bone marrow treated with CCS1477; SAM-KO-CON, in vitro-induced SAMs from *Acod1*$^{Mye-/-}$ mouse bone marrow treated with DMSO; SAM-KO-CCS, in vitro-induced SAMs from *Acod1*$^{Mye-/-}$ mouse bone marrow treated with CCS1477.

Major concerns

2. Although pharmacological inhibition of EP300 was shown to reduce the hepatic damage of ACLF via removal of SAM (Figure 3), it remains to be shown whether inhibition of EP300 affects other subsets of $CD11b^+F480^+$ macrophages in the liver.

Response: We thank the reviewer for this suggestion. As recommended, We evaluated the impact of EP300 inhibition on other $CD11b^+F4/80^+$ macrophage subsets identified in the murine single-cell dataset. Among these, we focused on subsets for which suitable antibodies were available, including MP2 ($Ccr5^+Cxcr3^+$), MP6 ($Cxcr2^+Cd9^+$), and MP7 ($Ccr3^+$). Flow cytometric analyses showed that treatment with the EP300 inhibitor CCS1477 did not alter the proportions of these macrophage subsets **(Fig.R20)**.

We have now included this result in the revised manuscript **(page 7, lines 248-249；New data Fig. S10F)**.

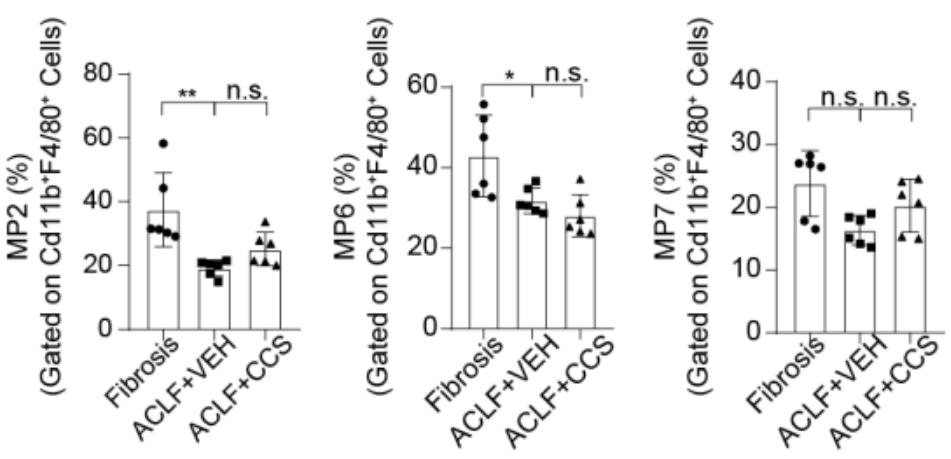

Figure R20. Ep300 inhibitor treatment in ACLF mice. Related to Figure S10F (New data). Flow cytometry analysis of the changes in the proportion of MP2 ($Ccr5^+Cxcr3^+$), MP6 ($Cxcr2^+Cd9^+$) and MP7 ($Ccr3^+$) within the $Cd11b^+F4/80^+$ gated population in liver of fibrosis and ACLF model mice with vehicle or CCS1477 (n = 6). $^{**}P < 0.01$, $^{*}P < 0.05$, *n.s.*, no significance. VEH, vehicle; CCS, CCS1477.

Major concerns

3. Related to 2, it is possible that EP300 can program the transcription of immune cells other than liver cells during ACLF. Indeed, a recent paper reported an Ep300-dependent transcriptional program regulating intestinal epithelial cell (IEC) activation during acute liver failure (Cell Discov. 2023 DOI: 10.1038/s41421-023-00578-4). Since it has been suggested that bacterial translocation is involved in the pathogenesis of ACLF, the authors should clarify this point.

Response: We thank the reviewer for raising this important point. As a key regulator of SAMs, EP300 directly enhanced their antibacterial immune responses. EP300 inhibition reprograms SAMs via ACOD1, thereby enhancing their antibacterial immune function, reducing bacterial load in ACLF patients, and alleviating tissue injury to alleviate disease progression **(Fig. R21)**.

Adoptive transfer of in vitro bone marrow-derived SAMs into ACLF mice confirmed that EP300 inhibition alleviates ACLF progression by enhancing SAM antibacterial immunity rather than acting on liver cells or other pathways.

EP300 regulates IECs in ACLF: EP300 regulates IECs and may influence bacterial translocation in ACLF, which is of potential interest. We have incorporated this point into the Discussion, highlighting it as a direction worthy of future investigation **(page XX, lines XX)**.

.

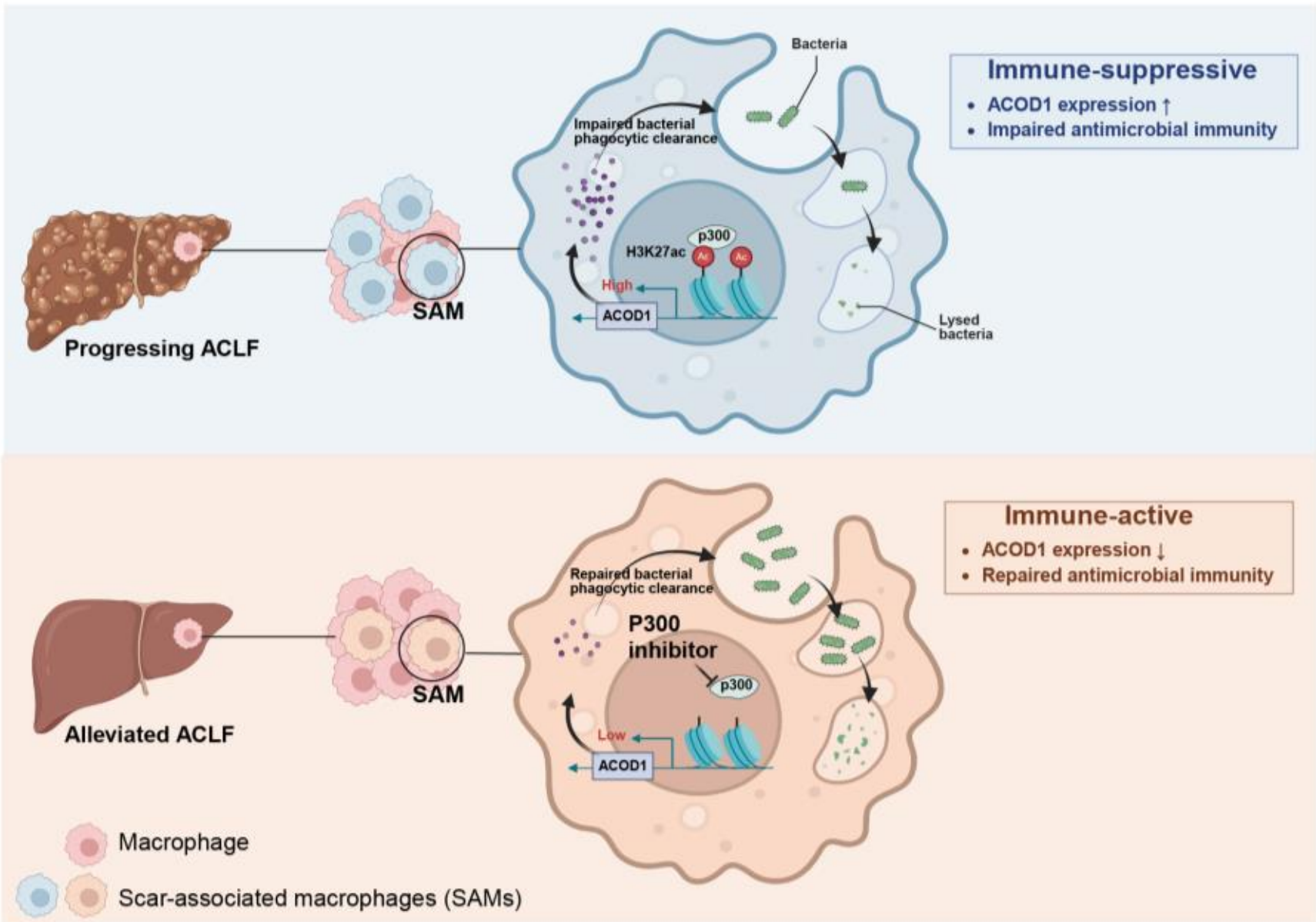

Figure R21. Schematic summary of EP300 inhibition alleviating ACLF by modulating SAMs via ACOD1. Related to Figure S14.

Minor concerns

1. In all flowcytometry data, please also show the representative FACS data.

Response: Thanks for these Comments. We have compiled all representative FACS data from the flow cytometry experiments and included them in the supplementary file entitled “PDF 1 FACS gating strategy and representative data”.

2. Please show longitudinal changes in serum transaminase and liver tissue in the ACLF model administered with Klebsiella pneumoniae compared to the steady state, liver fibrosis model.

Response: Thanks for this constructive suggestion. We have added a longitudinal analysis comparing the ACLF to the liver fibrosis model. In the ACLF mice, progressive disruption of intrahepatic fibrous septa were observed over time **(Fig. R22A)**. Serum ALT and TBIL levels peaked at 36 hours after the acute CCl_4 challenge and then declined **(Fig. S22B)**.

We have now included this result in the revised manuscript **(page 5, lines 175-179；New data Fig. S7)**.

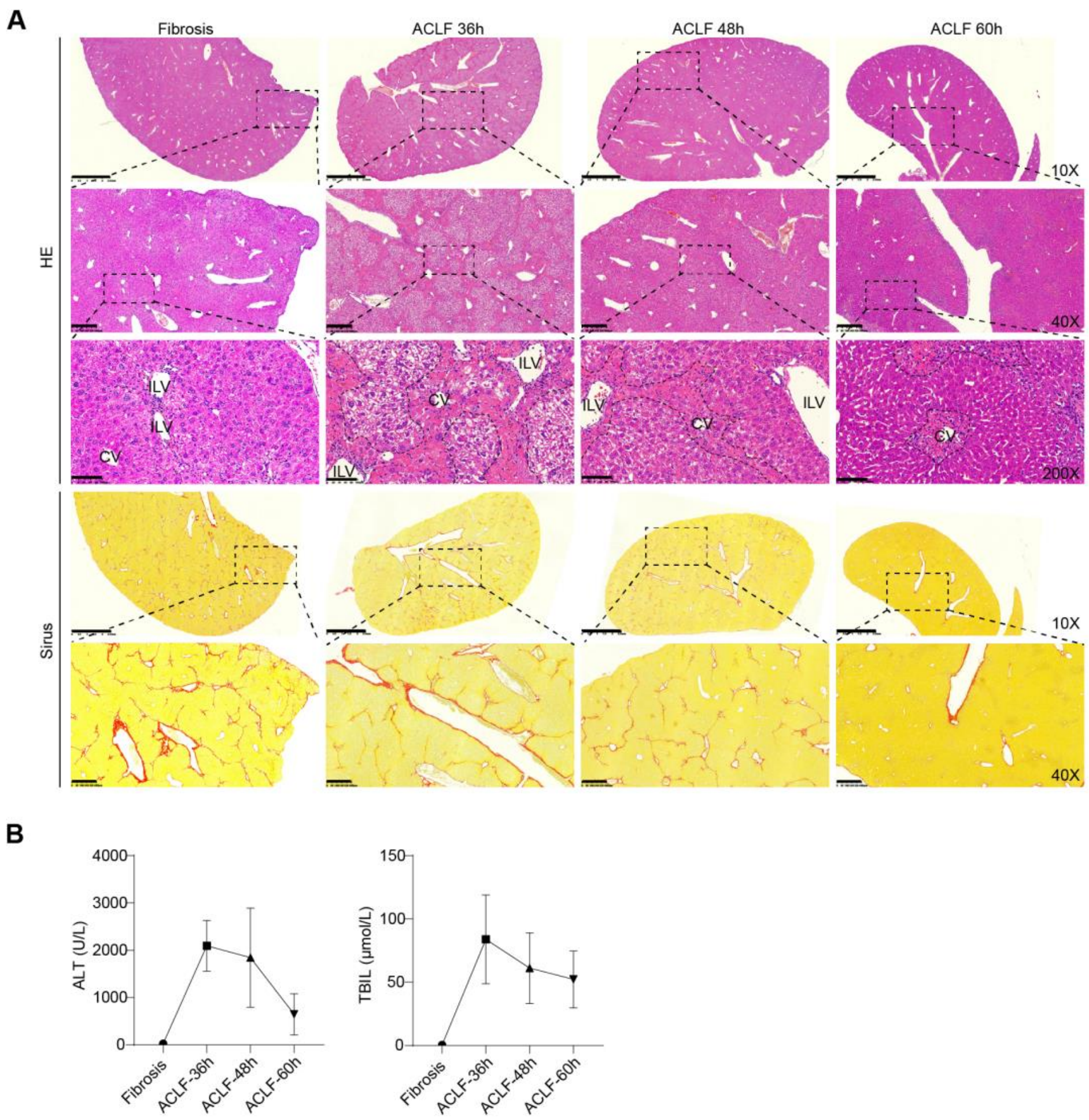

Figure R22. The pathological characteristics of fibrosis and ACLF mice. Related to Figure S7 (New data).

(A) Representative H&E and Sirus Red staining images of the mouse livers. Scale bars, 2.5 mm at 10x, 0.4 mm at 40x, and 0.1 mm at 200x. (B) Serum ALT and TBIL levels in ACLF model mice at different time points. ILV, interlobular vein; CV, central vein.

References

1 Li, H. *et al.* Submassive hepatic necrosis distinguishes HBV-associated acute on chronic liver failure from cirrhotic patients with acute decompensation. *J Hepatol* **63**, 50-59 (2015). https://doi.org:10.1016/j.jhep.2015.01.029

2 Ramachandran, P. *et al.* Resolving the fibrotic niche of human liver cirrhosis at single-cell level. *Nature* **575**, 512-518 (2019). https://doi.org:10.1038/s41586-019-1631-3

3 Gola, A. *et al.* Commensal-driven immune zonation of the liver promotes host defence. *Nature* **589**, 131-136 (2021). https://doi.org:10.1038/s41586-020-2977-2

4 Wu, R., Chen, F., Wang, N., Tang, D. & Kang, R. ACOD1 in immunometabolism and disease. *Cell Mol Immunol* **17**, 822-833 (2020). https://doi.org:10.1038/s41423-020-0489-5

5 Wu, R., Kang, R. & Tang, D. Mitochondrial ACOD1/IRG1 in infection and sterile inflammation. *J Intensive Med* **2**, 78-88 (2022). https://doi.org:10.1016/j.jointm.2022.01.001

6 Wu, R., Liu, J., Tang, D. & Kang, R. The Dual Role of ACOD1 in Inflammation. *J Immunol* **211**, 518-526 (2023). https://doi.org:10.4049/jimmunol.2300101

7 Lampropoulou, V. *et al.* Itaconate Links Inhibition of Succinate Dehydrogenase with Macrophage Metabolic Remodeling and Regulation of Inflammation. *Cell Metab* **24**, 158-166 (2016). https://doi.org:10.1016/j.cmet.2016.06.004

8 Nair, S. *et al.* Irg1 expression in myeloid cells prevents immunopathology during M. tuberculosis infection. *J Exp Med* **215**, 1035-1045 (2018). https://doi.org:10.1084/jem.20180118

9 Daniels, B. P. *et al.* The Nucleotide Sensor ZBP1 and Kinase RIPK3 Induce the Enzyme IRG1 to Promote an Antiviral Metabolic State in Neurons. *Immunity* **50**, 64-76.e64 (2019). https://doi.org:10.1016/j.immuni.2018.11.017

10 Domínguez-Andrés, J. *et al.* The Itaconate Pathway Is a Central Regulatory Node Linking Innate Immune Tolerance and Trained Immunity. *Cell Metab* **29**, 211-220.e215 (2019). https://doi.org:10.1016/j.cmet.2018.09.003

11 Li, Y. *et al.* Immune responsive gene 1 (IRG1) promotes endotoxin tolerance by increasing A20 expression in macrophages through reactive oxygen species. *J Biol Chem* **288**, 16225-16234 (2013). https://doi.org:10.1074/jbc.M113.454538

12 Fernández, J. *et al.* Bacterial and fungal infections in acute-on-chronic liver failure: prevalence, characteristics and impact on prognosis. *Gut* **67**, 1870-1880 (2018). https://doi.org:10.1136/gutjnl-2017-314240

13 Xiang, X. *et al.* Interleukin-22 ameliorates acute-on-chronic liver failure by reprogramming impaired regeneration pathways in mice. *J Hepatol* **72**, 736-745 (2020). https://doi.org:10.1016/j.jhep.2019.11.013

14 Engelmann, C., Mehta, G. & Tacke, F. Regeneration in acute-on-chronic liver failure - the phantom lost its camouflage. *J Hepatol* **72**, 610-612 (2020). https://doi.org:10.1016/j.jhep.2020.01.003

15 Zhai, H., Zhang, J., Shang, D., Zhu, C. & Xiang, X. The progress to establish optimal animal models for the study of acute-on-chronic liver failure. *Front Med (Lausanne)* **10**, 1087274 (2023). https://doi.org:10.3389/fmed.2023.1087274

16 Fabre, T. *et al.* Identification of a broadly fibrogenic macrophage subset induced by type 3 inflammation. *Sci Immunol* **8**, eadd8945 (2023). https://doi.org:10.1126/sciimmunol.add8945

Point-by-point responses

We are very grateful to the reviewers for providing us with very helpful, consistent, and constructive feedback on our work to revise and improve our manuscript.

We have conducted several new experiments, performed new analyses, and made several modifications throughout the manuscript and its figures to address all the reviewers' concerns. We have included detailed, point-by-point responses to each of these concerns, describing the corresponding changes in our manuscript. Our responses are highlighted in blue text to facilitate the review process.

Nature Communications

Manuscript Number: NCOMMS-24-69199B

"EP300 inhibition alleviates acute-on-chronic liver failure through modulation of scar-associated macrophages"

REVIEWER COMMENTS

Reviewer #1 (Comments to the Author):

Reviewer #1 (Remarks to the Author):

I would like to congratulate the authors for their effort in improving the manuscript. The revised version indeed shows substantial progress. However, I still have concerns regarding two major aspects: (1) the spatial organization of SAMs relative to injury sites and preserved hepatocytes, and (2) the Acod1-related mechanisms. Both elements are central to the authors' mechanistic argument and require a solid evidential basis. My comments mainly relate to the new data and newly added figures, but also reflect general considerations.

Response: We sincerely thank the reviewer for the positive evaluation and constructive comments on our work.

1.Figure R1:

The CK19 staining appears convincing. However, I have doubts regarding the HNF4a and Trem2 staining:

• HNF4a is expected to be nuclear, while the presented images show predominantly cytoplasmic staining, alternative use Hepar-1 rather than HNF4a.

• Trem2 appears to contain substantial background signal, with limited clear association with DAPI. I have difficulties in finding a reliable Trem2 staining.

Additionally, Trem2 seems to stain virtually all cells, including hepatocytes. Is this expected? If so, what is the functional implication of Trem2 expression in hepatocytes?

Response: We thank the reviewer for these valuable suggestions. Based on the reviewer's suggestion, we replaced HNF4α with HepPar-1 as a hepatocyte marker **(Fig. R1)**, which provides more reliable hepatocyte identification.

We also optimized the TREM2 staining conditions to reduce background signal. In HBV-related liver cirrhosis and ACLF samples, TREM2 shows signals in some hepatocyte-like regions **(Fig. R1)**. However, our single-cell RNA-seq data indicate that TREM2 expression is restricted to mononuclear phagocyte (MP) populations and is not detected in hepatocytes **(Fig. R2)**. Therefore, we do not interpret these hepatocyte-associated TREM2 signals as evidence of true hepatocyte expression. Instead, they likely reflect HBV-associated hepatocellular alterations that affect antigen accessibility or protein distribution. A similar phenomenon may explain the cytoplasmic shift of HNF4α staining in HBV-related liver cirrhosis and ACLF samples.

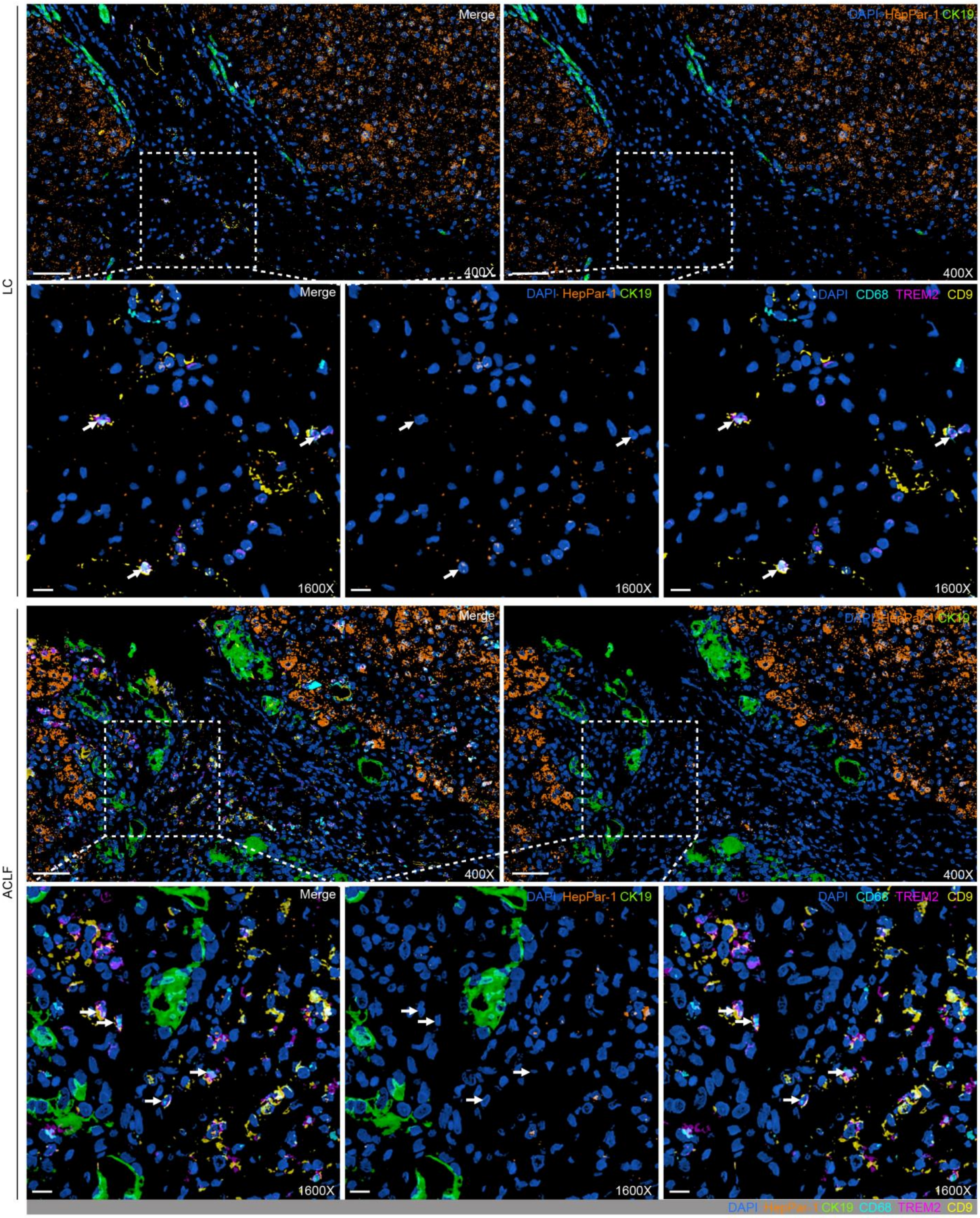

Figure R1. SAMs infiltration sites in HBV-related LC and HBV-related ACLF patients. Related to Figure S4 (New data). Hepatocyte marker (HepPar-1) and cholangiocyte marker (CK19) were used to identify liver structures, while CD68, TREM2, and CD9 were used to identify SAMs (White arrows). Scale bars, 0.05 mm at 400x and 0.025 mm at 1600x. LC, liver cirrhosis; ACLF, Acute-on-Chronic Liver Failure.

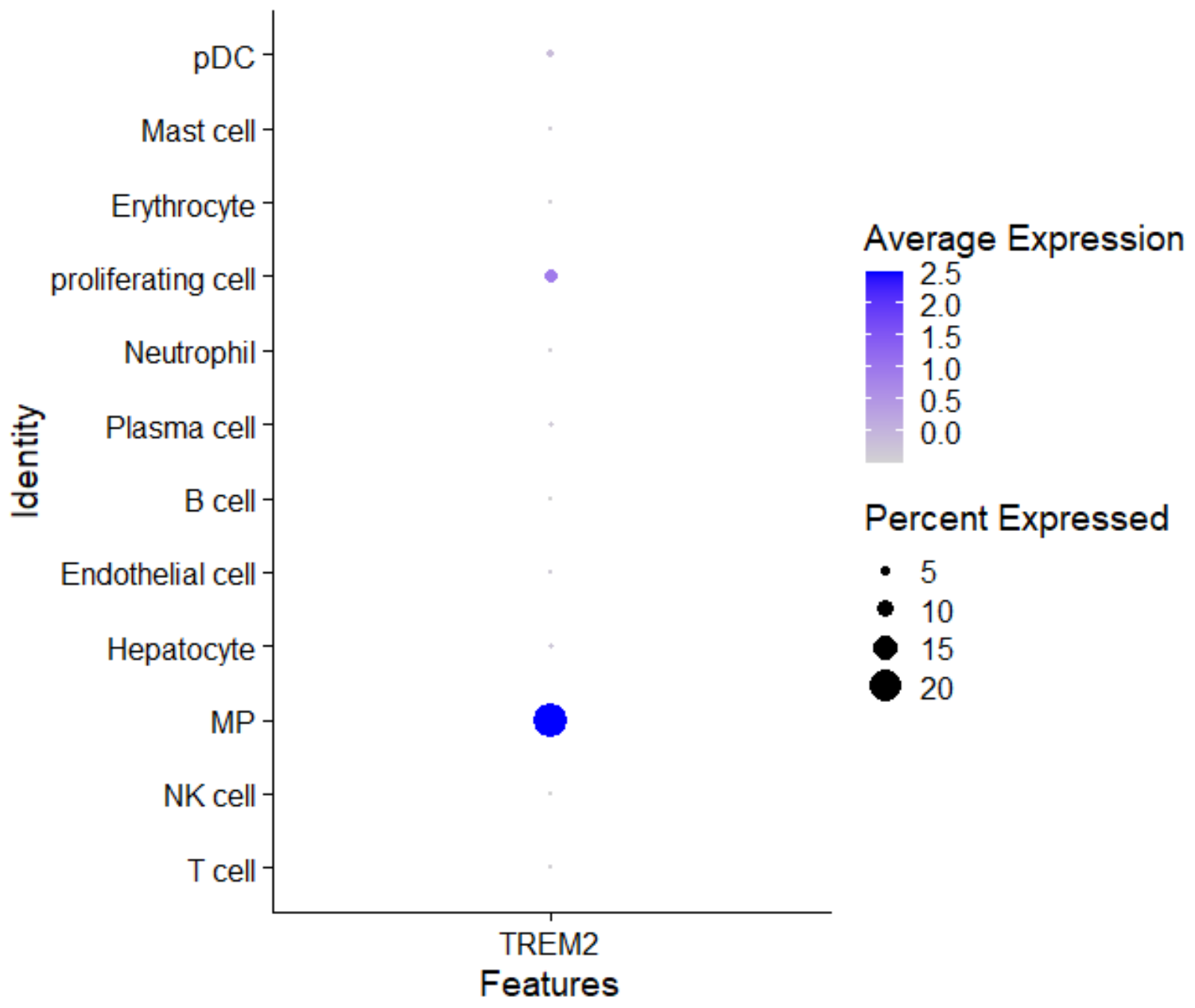

Figure R2. Cell type specific expression of TREM2 in human liver single cell RNA sequencing data (New data).
Dot plot showing TREM2 expression across major hepatic cell populations. Dot size represents the percentage of cells expressing TREM2, and color intensity indicates the average expression level. MP, mononuclear phagocytes.

2.Figure R4:

HNF4a staining appears reliable at the borders of preserved hepatocyte islands. However, the Trem2 staining again raises concerns: low concordance with DAPI and apparent Trem2 positivity in some hepatocytes. If these signals are indeed specific, the data would suggest that SAMs localize predominantly to regions of ductular reactions—areas characterized by biliary progenitor cell responses and strong CK19 expression. What is the proposed mechanism in this region?

Response: We thank the reviewer for these suggestions. In alcohol-related ACLF samples, we retained HNF4α staining and further optimized the TREM2 staining conditions, which reduced background signal and improved signal clarity. With the optimized TREM2 staining, SAMs can be clearly identified. Their distribution is consistent with enrichment in portal regions surrounding collagen-positive scar areas **(Fig. R3)**. Ductular reactions were observed in portal regions in alcohol-related ACLF liver. While SAMs are observed in similar regions, we did not specifically investigate the spatial relationship between SAMs and ductular reactions in this study. The co-localization of SAMs with ductular reactions likely reflects former may related to tissue architecture remodeling. Whether a direct mechanistic interaction exists between these two components requires further investigation.

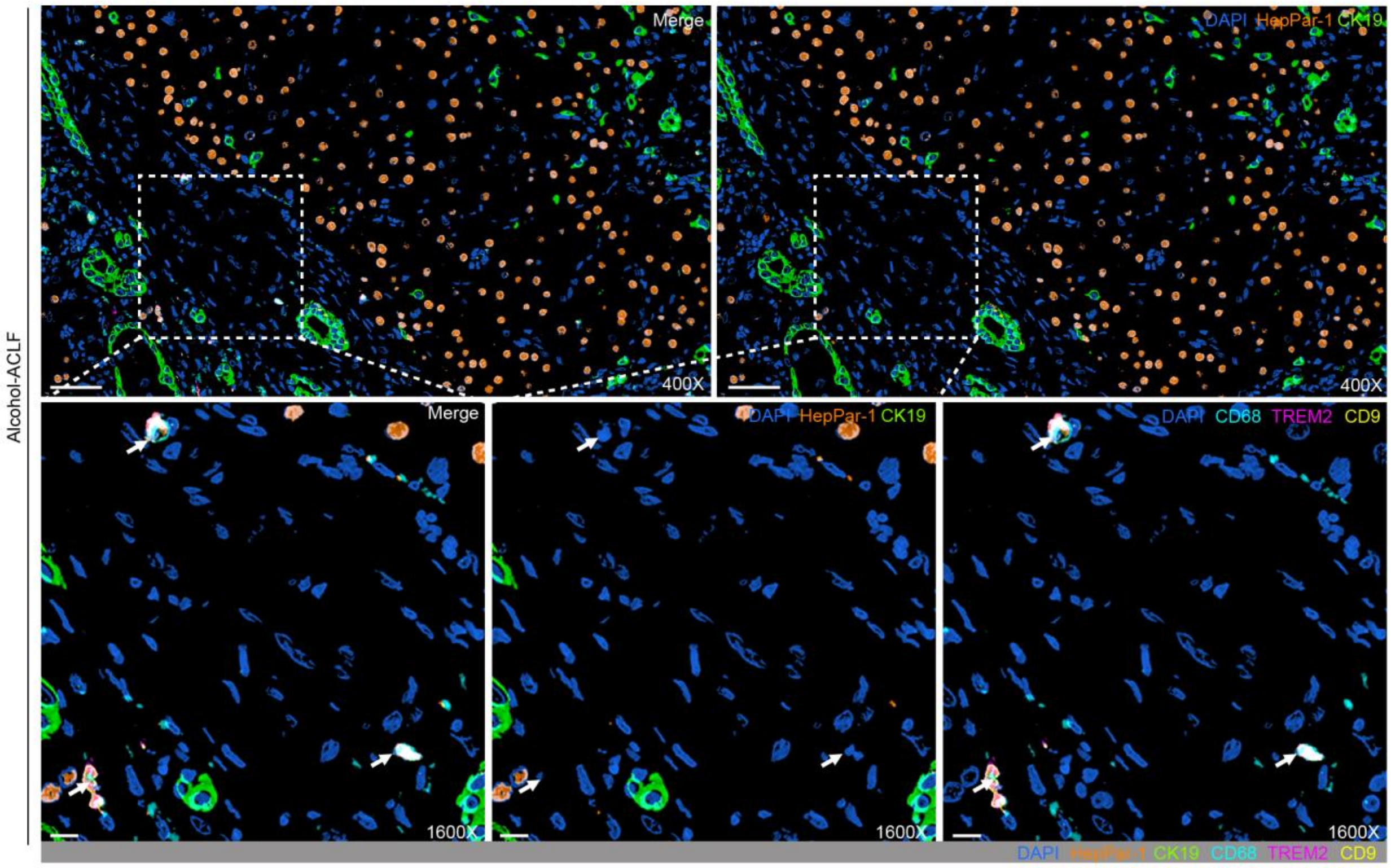

Figure R3. SAMs infiltration sites in alcohol-related ACLF patients. Related to Figure S5 (New data).
Representative mIHC images of the ACLF patients. Scale bars, 0.05 mm at 400x and 0.025 mm at 1600x. Hepatocyte marker (HNF4α) and cholangiocyte marker (CK19) were used to identify liver structures; CD68, TREM2, and CD9 were used to identify SAMs (White arrows).

3.Figure R5:
The overall staining shown in this figure appears even less distinct, with what looks like considerable nonspecific/background signal. Given this, I am not confident that the images allow firm conclusions about spatial organization of SAMs or zonal distribution.

Response: We thank the reviewer for this important comment. Based on the reviewer's suggestion, we replaced HNF4α with HepPar-1 as a hepatocyte marker and further optimized the TREM2 staining conditions to reduce background signal across all figures **(Fig. R4)**.

Across the revised datasets, SAMs' distribution is consistent with enrichment in portal regions surrounding collagen-positive scar areas **(Fig. R4**, in line with our observations in patient samples **(Fig. R1, R3)**. However, we acknowledge that these images alone do not allow definitive conclusions regarding precise spatial or zonal organization. Accordingly, we have added this point to the limitations section of the Discussion, noting that further validation using orthogonal spatial approaches will be required to more precisely define SAM localization (Revised manuscript, page 16, lines 505-508).

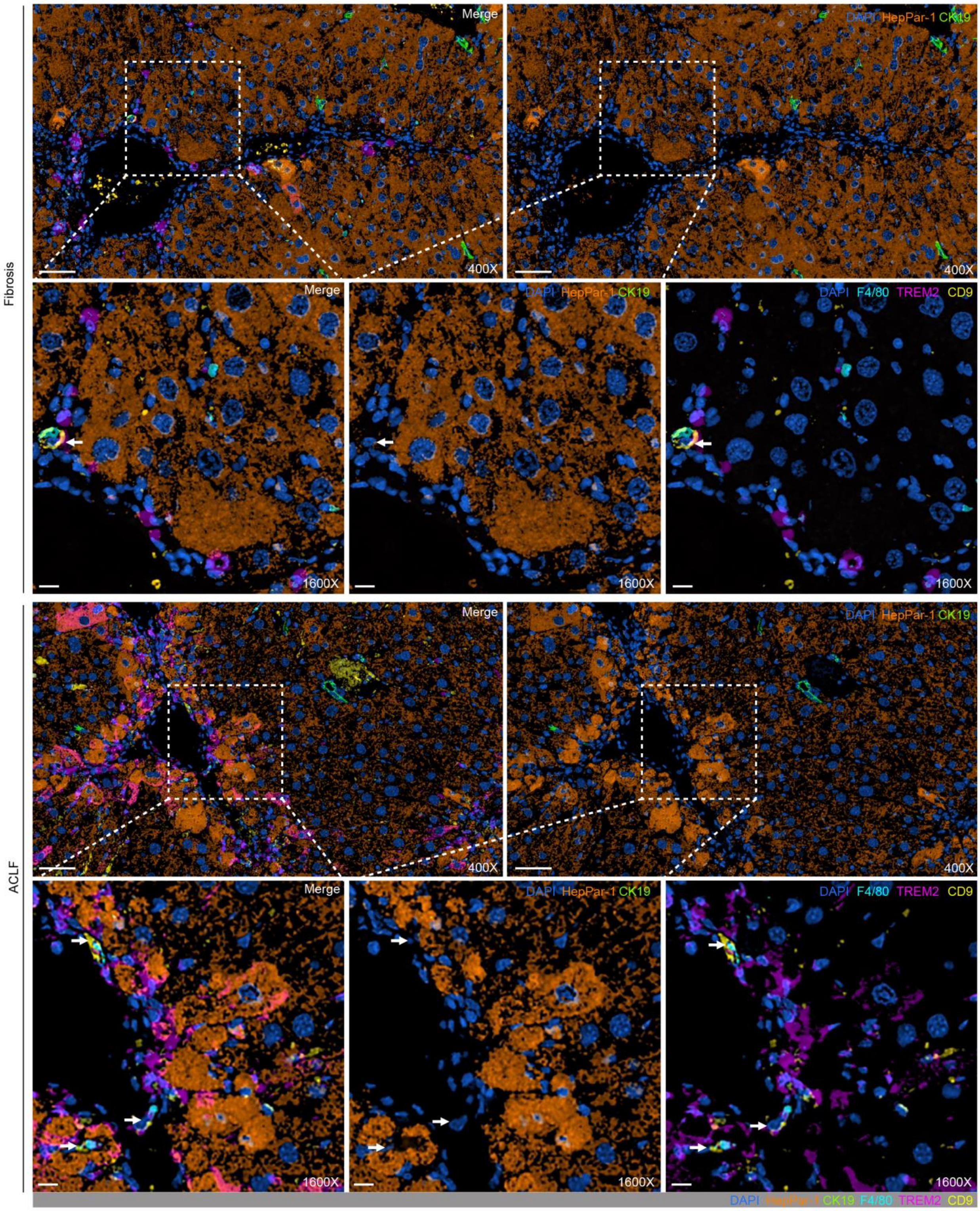

Figure R4. SAMs infiltration sites in fibrosis and ACLF mice. Related to Figure S8 (New data).

Representative mIHC images of the mouse livers. Hepatocyte marker (HepPar-1) and cholangiocyte marker (CK19) were used to identify liver structures, while F4/80, TREM2, and CD9 were used to identify SAMs (White arrows). Scale bars, 0.05 mm at 400x and 0.025 mm at 1600x.

4.Acod1Mye−/− Mouse Model in Chronic CCl_4 Injury:

The reported phenotype in the CCl4 chronic face is puzzling. The Acod1 knockout appears detrimental in the chronic CCl_4 model but protective in the acute injury model. What is the mechanistic explanation for this discrepancy?

Response: We thank the reviewer for this important comment. The chronic CCl_4 model and the ACLF mouse model with infection represent distinct biological contexts. Chronic CCl_4 injury is a sterile process dominated by fibrogenic remodeling. In contrast, ACLF involves infection and impaired antibacterial immunity.

Our data suggest that Acod1 has context-dependent effects. In sterile chronic injury (chronic CCl_4 model), Acod1 may contribute to inflammatory remodeling. In ACLF, Acod1 upregulation in SAMs is associated with impaired phagocytic function. Myeloid-specific *Acod1* deficiency improves bacterial clearance and attenuates disease progression.

Therefore, the different phenotypes likely reflect differences in disease context rather than contradictory functions of Acod1.

Similarly, the new in vitro stimulation data (page 9, lines 300–306, and page 10, lines 307–310) are difficult to reconcile. Do SAMs from Acod1Mye−/− mice exhibit reduced or increased phagocytic capacity compared to WT? The text suggests potentially conflicting findings. How do these results align with the increased injury observed in the chronic CCl_4 model? Additionally, in both Figure 4 and Figure S12, the combination of Acod1 KO and CCS appears not to enhance the beneficial effects of Acod1 deficiency, but instead to reduce phagocytosis. What is the authors' explanation for this unexpected result?

Response: We thank the reviewer for these comments. The apparent inconsistency arose from the previous figures, which contained excessive information and may have led to misinterpretation. We have now replaced them with independently repeated experiments and a clearer presentation. We have now included this point in the revised manuscript (page 9-10, lines308-314； Fig. S12C-S12D).

Our results indicate that upon LPS or PMA stimulation, SAMs from WT mice exhibit reduced phagocytic capacity compared with unstimulated controls, indicating impaired antibacterial function under ACLF-like inflammatory and oxidative stress conditions. In contrast, SAMs from $Acod1^{Mye-/-}$ mice display increased phagocytic capacity compared with WT SAMs under the same stimulation conditions **(Fig. R5)**. These findings indicate that *Acod1* deficiency enhances the antibacterial phagocytic function of SAMs.

Regarding the combined treatment, *Acod1* deficiency together with CCS1477 does not further enhance the beneficial effect observed in Acod1-deficient SAMs **(Fig. R5)**, which indicates that EP300 regulates SAM phagocytic function primarily through ACOD1.

Together, these findings demonstrate that under ACLF-like conditions, Acod1 deficiency restores impaired SAM phagocytosis, and support a model in which EP300 inhibitor CCS1477 restored SAM antibacterial phagocytic function in an ACOD1-dependent manner. The lack of further enhancement with combined treatment suggests that ACOD1 is a key mediator of this process.

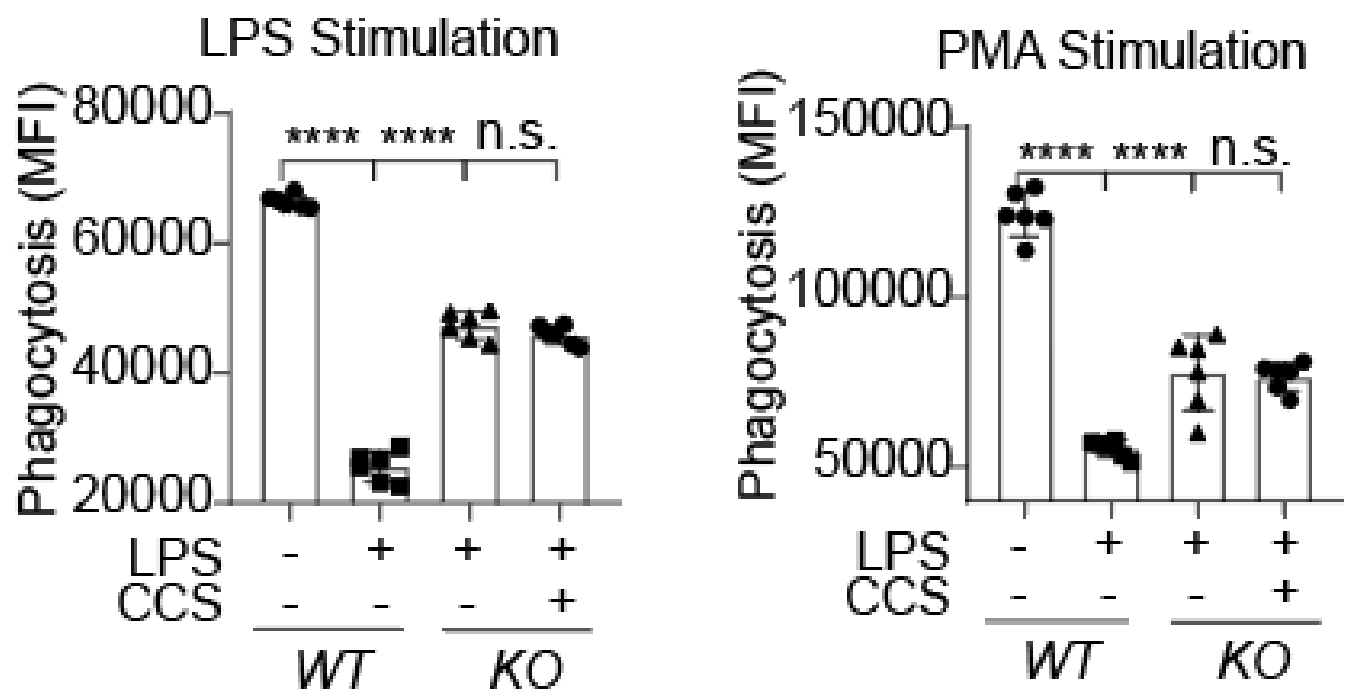

Figure R5. Induction of SAMs *in vitro* and assessment of their bacterial phagocytic capacity under ACLF-like conditions. Related to Figure S12 (New data).

Phagocytosis of *E. coli* by induced SAMs under indicated conditions (± LPS/PMA, ± CCS1477), accessed by flow cytometry (n = 6). Bars represent mean ± SD; *****P* < 0.0001, *n.s.*, no significance. CCS, CCS1477; MFI, Mean Fluorescence Intensity; WT *in vitro*-induced SAMs from WT mouse bone marrow; KO, *in vitro*-induced SAMs from $Acod1^{Mye-/-}$ mouse bone marrow.

Reviewer #3 (Remarks to the Author):

In this revised paper, in response to major issues raised in the previous round of review, such as whether SAMs (scar-associated macrophages) truly drive ACLF (acute-on-chronic liver failure), the authors replaced depletion experiments with functional enhancement experiments (adoptive transfer) to circumvent technical limitations, emphasizing functional restoration rather than cell quantity. Meanwhile, to determine whether the mechanism is confined to SAMs, the authors excluded the possibility of other cell types playing a dominant role through subgroup analysis, functional validation of Acod1, and EP300 co-localization studies. Regarding whether the mouse model represents human ACLF, in the new version, it is emphasized that bacterial infection-induced ACLF accounts for two-thirds of clinical cases, and the localization and function of SAMs are consistent across species, indicating the model's translational value. Furthermore, addressing concerns about whether the EP300-ACOD1 axis has a direct relationship, the authors employed multi-level evidence, including ChIP-seq, CUT&Tag, and adoptive transfer experiments, to establish direct regulation and functional causality. Overall, the authors have made substantial improvements to the article in response to the former comments from the previous round of review.

Response: We sincerely thank the reviewer for the positive evaluation and constructive comments on our work.

The following lists some new comments. First, in the causality validation, the authors have not yet utilized a genetic model specifically targeting the depletion of SAMs. Therefore, the authors need to discuss the necessity of SAMs in ACLF. At present, the article only employs the CCl4 plus bacterial infection model, failing to cover other common ACLF triggers such as alcohol and drugs.

Response: We thank the reviewer for this important comment. We agree that a genetic model enabling specific depletion of SAMs would provide stronger evidence for the necessity of SAMs in ACLF progression. However, currently there is no well-established genetic model that selectively targets SAM depletion. Therefore, we have included this point as a limitation in the Discussion (Revised manuscript, page 16, lines 502-503). As a functional alternative, we performed adoptive transfer experiments of SAMs.

Regarding ACLF triggers, we agree that ACLF can be induced by multiple factors, including alcohol and drug-induced liver injury. In this study, we focused on infection-associated ACLF, as up to two-thirds of patients develop infections at diagnosis or during follow-up, regardless of etiology, with bacterial infection representing a major determinant of prognosis. Accordingly, we will further clarify in the Discussion that our findings highlight that EP300 inhibition modulates SAM antibacterial phagocytic function in the context of ACLF with infection， and whether this regulatory mechanism is applicable to ACLF withou infection remains to be determined. In addition, ACLF models for other etiologies are not yet well established, and their development will be important to assess the generalizability of this mechanism across different triggers (Revised manuscript, page 16, lines 508-511).

Second, the theme of this study is the discovery that EP300 regulates the phagocytic function of SAMs through ACOD1, thereby influencing the progression of ACLF. The content lacks a mechanistic diagram. Although the authors have provided a schematic in the graphical abstract, it is recommended to include a pathway diagram in the main text as well. This diagram could also be used to summarize the main results of the article. The current article structure makes it difficult to discern an inherent logic easily.

Response: We thank the reviewer for this valuable suggestion. In the revised manuscript, we have included a mechanistic pathway diagram in the main text **(Fig. R6)** to summarize the conceptual framework and major findings of this study.

The diagram illustrates that EP300-mediated histone acetylation promotes *ACOD1* transcription in SAMs. This leads to impaired antibacterial phagocytic function and reduced bacterial clearance, thereby contributes to ACLF progression. Pharmacological inhibition of EP300 decreases *ACOD1* expression, restores phagocytic function, enhances bacterial clearance, and ultimately alleviates ACLF.

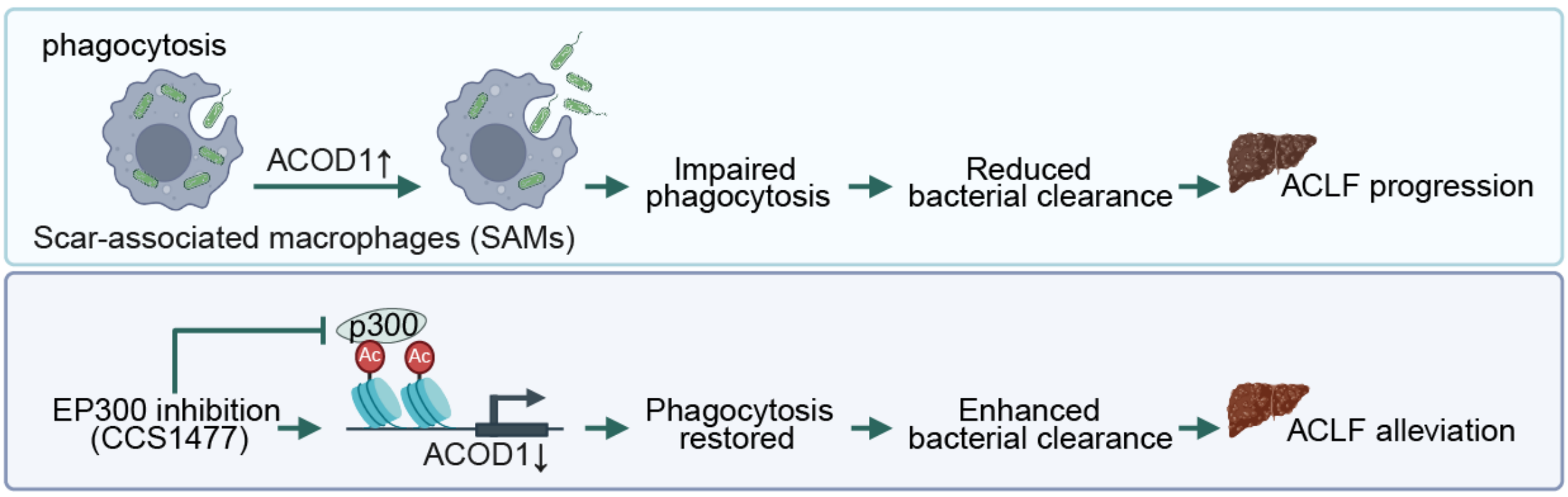

Figure R6. EP300–ACOD1 regulation of SAM phagocytosis in ACLF. Related to Figure 6F (New data).

This diagram provides a visual summary of the mechanistic pathway identified in this study and helps clarify the overall logical structure of the manuscript. We have also revised the Results and Discussion sections to better align with this mechanistic framework.

Third, the authors utilized data from different sources but did not rigorously examine batch effects or important information such as sample gender. This may lead to bias in the results.

Response: We thank the reviewer for raising this essential point. For the single-cell RNA-seq data, batch effects were corrected using the Harmony algorithm during data integration to minimize technical variation across samples. For bulk transcriptomic datasets from different sources, analyses were performed separately and standardized normalization procedures were applied to reduce potential batch effects. These points have now been clarified in the Methods section.

For some external datasets, complete clinical metadata were not available, and important variables such as sample sex could not be fully controlled. This may introduce potential bias and should be taken into consideration when interpreting the transcriptomic analyses. This limitation has now been added to the Discussion section (Revised manuscript, page 16, lines 512-514).
